# Bridging the human–AI knowledge gap through concept discovery and transfer in AlphaZero

**DOI:** 10.1073/pnas.2406675122

**Published:** 2025-03-26

**Authors:** Lisa Schut, Nenad Tomašev, Thomas McGrath, Demis Hassabis, Ulrich Paquet, Been Kim

**Affiliations:** ^a^Oxford Applied and Theoretical Machine Learning Group, Department of Computer Science, University of Oxford, Oxford OX1 3QG, United Kingdom; ^b^Google DeepMind, London N1C 4AG, United Kingdom; ^c^Goodfire AI, San Francisco, CA 94102; ^d^Google DeepMind, Mountain View, CA 94043

**Keywords:** machine learning, AI, reinforcement learning, concept discovery

## Abstract

AI systems have attained superhuman performance across various domains. If the hidden knowledge encoded in these highly capable systems can be leveraged, human knowledge and performance can be advanced. Yet, this internal knowledge is difficult to extract. Due to the vast space of possible internal representations, searching for meaningful new conceptual knowledge can be like finding a needle in a haystack. Here, we introduce a method that extracts new chess concepts from AlphaZero, an AI system that mastered chess via self-play without human supervision. Our method excavates vectors that represent concepts from AlphaZero’s internal representations using convex optimization, and filters the concepts based on teachability (whether the concept is transferable to another AI agent) and novelty (whether the concept contains information not present in human chess games). These steps ensure that the discovered concepts are useful and meaningful. For the resulting set of concepts, prototypes (chess puzzle–solution pairs) are presented to experts for final validation. In a preliminary human study, four top chess grandmasters (all former or current world chess champions) were evaluated on their ability to solve concept prototype positions. All grandmasters showed improvement after the learning phase, suggesting that the concepts are at the frontier of human understanding. Despite the small scale, our result is a proof of concept demonstrating the possibility of leveraging knowledge from a highly capable AI system to advance the frontier of human knowledge; a development that could bear profound implications and shape how we interact with AI systems across many applications.

## Significance

As AI systems become more capable, they may internally represent concepts outside the sphere of human knowledge. This work gives an end-to-end example of unearthing machine-unique knowledge in the domain of chess. We obtain machine-unique knowledge from an AI system (AlphaZero) by a method that finds novel yet teachable concepts and show that it can be transferred to human experts (grandmasters). In particular, the new knowledge is learned from internal mathematical representations without a priori knowing what it is or where to start. The produced knowledge from AlphaZero (new chess concepts) is then taught to four grandmasters in a setting where we can quantify their learning, showing that machine-guided discovery and teaching is possible at the highest human level.

Traditionally, AI systems are treated as problem-solving machines; they can carry out the jobs humans are capable of but more efficiently or with less effort, which brings clear benefits in several domains. In this paper, we pursue a different goal: treat AI systems as learning machines and demand them to teach us the fundamental principles behind their decisions to complement and extend upon our knowledge. There is a tremendous untapped opportunity across various domains where the capabilities of AI systems are reaching or exceeding those of human experts (superhuman AI systems). This work is one of the first steps toward the development of tools and methods that allow us to uncover new knowledge that is encoded in highly capable AI systems, and empower human experts by helping them further improve their skills and understanding.

The superhuman capabilities of AI systems may arise in a few different ways: pure computational power of machines, new ways of reasoning over existing knowledge, or superhuman knowledge we do not possess. We focus on the last two cases, and for simplicity, refer to both as superhuman knowledge from now on.

## 1.1. The Representation of Superhuman Knowledge.

**Fig. 1.**
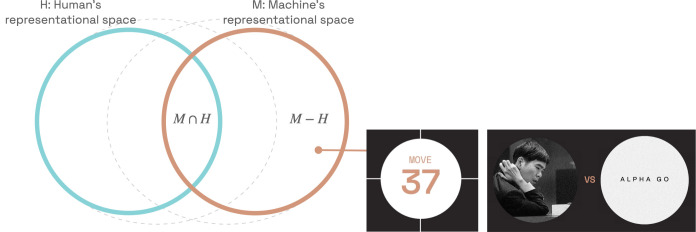
Learning from machine-unique knowledge. The pink circle represents what machines know (M) and the blue circle represents what humans know (H). Our work focuses on (M−H)—knowledge that is unique to machines. One prominent example of machine-unique knowledge is move 37 in the AlphaGo–Lee Sedol match.

Abstractly, we use H to denote the human’s vocabulary—concepts, knowledge, and the capabilities they enable—and M the equivalent for machines. There are things that both an advanced AI system and humans “know” (M∩H), things that only humans know (H−M), and things only machines know (M−H) as shown in Fig. 1.

Most existing research efforts only focus on (M∩H), e.g., interpretability research has tried to shoehorn M into (M∩H), with limited success (1–3). We believe that the knowledge gap represented by (M−H) is crucial to empowering humans, by identifying new concepts and discovering new connections between existing concepts within highly capable AI systems.

One prominent example of (M−H) in the history of AI is move 37—an exceptionally strong, novel idea—that AlphaGo made in the second game of its match against Lee Sedol (4). This move surprised both commentators and Lee Sedol and is still discussed to this day as an example of superhuman knowledge (Fig. 1). If there are more such examples, exemplifying new conceptual knowledge that enables new capabilities, we would like to discover them using a structured approach.

## 1.2. Conceptual Knowledge in Chess.

We focus on a domain that has inspired AI practitioners for decades, and captivated human imagination for centuries: the game of chess. Chess is an excellent playground to validate the existence and utility of (M−H) for several reasons.

First, chess knowledge has been developed over a long period of time, providing a well-scoped estimate of H. Second, evaluating the utility of concepts and capabilities is easier to validate compared to the frontiers of other fields, such as science or medicine. In chess, the Elo rating is a quantitative measure of the quality of the capabilities of, both human experts and machines (5).

Most importantly, chess engines have performed at a superhuman level for a long time, ever since DeepBlue’s match against Garry Kasparov in 1996, suggesting that (M−H) may exist in this domain. While early engines were based on human knowledge, the advent of AlphaZero (6) (AZ) showed that a self-taught deep learning model can achieve superhuman capabilities in chess without any human knowledge. However, humans have largely tapped into these engines’ knowledge by querying these systems to evaluate specific chess positions or moves.^*^ By studying AZ’s games, humans have manually distilled patterns. However, this approach still analyzes M through the lens of H, a bias that limits what we can discover from M∩H. In this work, we take a step toward discovering (M−H) in AlphaZero is an unsupervised way. While we focus on AlphaZero, the methods that we outline could form a framework for general concept discovery in other systems, although we do not explore this in this paper.

## 1.3. Learning from AlphaZero.

We hypothesize that (M−H) exists and can be taught to humans. In this work, we first find evidence that (M−H) exists by analyzing the rank of the span of the internal representations of AZ’s and the human’s games. We validate our hypothesis by showing that we can teach new chess concepts to experts in the form of four top human chess grandmasters. Due to their undeniable strength, and talent, (M−H) may fall into their ‘proximal zone of development’ in Vygotsky’s education theory: “the space between what a learner can do without assistance and what a learner can do with adult guidance or in collaboration with more capable peers” (7). While communicating (M−H) may require new language (8) in other domains, we bypass this need in this work by leveraging chess champions’ ability to identify and learn new concepts from patterns that arise in examples of optimal play in chess positions.

## 1.4. Method for Concept Discovery.

We define a concept as a unit of knowledge, that can be represented as a vector in AZ’s internal representation space. We develop a framework to search for concepts in (M−H), i.e., unearth AZ’s superhuman knowledge. In this framework, we:

- develop a method for finding unsupervised concept vectors. Using the complete AZ machinery, the policy value network and Monte Carlo Tree Search (MCTS), our method discovers dynamic concepts: concepts that motivate a sequence of actions in chess. We verify the performance of our method in the supervised setting, where we show that our method can find high-quality concept vectors in a data-efficient manner.
- ensure that the concepts are teachable, i.e., informative and learnable. We develop a metric that evaluates whether concepts are teachable to another AI agent with no prior knowledge of the concept. Through this metric, we select concepts based on their informativeness (i.e., usefulness for an AI agent in a downstream task).
- ensure concepts are novel, i.e. in (M−H). Through spectral analysis, our framework only selects concepts that contain information unique to the vector space of AZ’s games compared to that of human-played games.
- provide insight into the meaning of the discovered concepts via graph analysis to reveal new concepts’ relations to human-labeled concepts.

Mathematically, we represent a concept c as a sparse vector $v_{c}$, and describe concept characteristics through inequality constraints that use $v_{c}$, be it in combination of neural network activations for single board positions or sequences of moves (and hence board positions). A concept prototype is then another sequence of board positions for which all the inequality constraints that use $v_{c}$ also hold; they are chess positions that exemplify the use of a concept.

### 1.4.1. Example concept prototype.

**Fig. 2.**
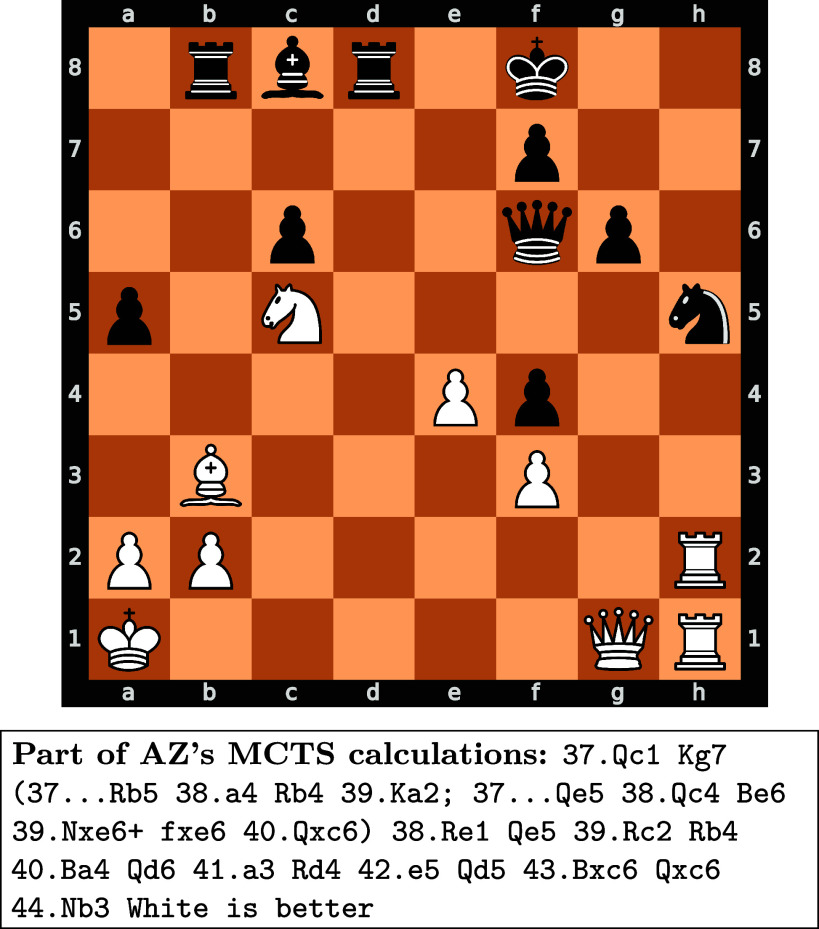
Example of a concept prototype. For each concept, we find prototypes: different positions whose solution sequences require applying the same underlying concept. It is difficult to label these concepts without oversimplifying them—however, the concept shown in this example is roughly dynamic piece maneuvers that span the chess board. Most chess players would opt for Rxh5, however, AZ plays Qc1, with the idea of regrouping the pieces to the queenside. Further details can be found in *SI Appendix*, Fig. S1.

Fig. 2 gives an example of a concept prototype: a plan (or sequence of moves) that satisfies all the “concept constraints” for some $v_{c}$. The concept is useful because it is teachable: Showing similar concept prototypes to an AI agent leads to a faster improvement in playing such positions (whereas a random v would lead to no improvement). Further, using a spectral-analysis-based novelty score, we show that the concept is likely in (M−H). In Fig. 2, most chess players would continue playing on the kingside^†^ with Rxh5, as the positioning of White’s most powerful pieces (the queen and the rooks) enables White to launch an attack against the weakened Black king on f8, hoping to break through. This is a natural way to try to win in chess as the goal of the game is to checkmate^‡^ the opposing king. However, AZ finds the only plan to maintain an advantage: Qc1 with the idea of remaneuvering the pieces to the queenside. This plan is counterintuitive as it reduces the immediate pressure against the Black’s king position, and takes a long time to execute, which gives Black the opportunity to take the time to counter the plan and improve its position. Nevertheless, this proves to be the correct approach.

### 1.4.2. Proof of concept: Teaching experts.

After finding novel and teachable unsupervised concepts in (M−H), we analyze whether we can expand the human representational space (H) to include these new concepts. We collaborate with four world top chess grandmasters and former and current World Champions^§^ to test whether they could learn and apply the concepts, by learning from prototypical examples like that of Fig. 2.

The results of our study suggest an improvement in the grandmasters’ ability to find concept-based moves aligned with AZ’s choices compared to their performance prior to teaching (i.e., observing AZ’s moves). Further, the grandmasters’ qualitative feedback indicated an understanding and appreciation of AZ’s plans. The discovered concepts often combine and apply chess concepts in a way that deviates from the traditional human principles of chess. We conjecture that the differences in humans’ and AZ’s play may stem from their differences in how position–concept relationships are built. While humans develop prior biases over which concepts may be relevant in given chess positions, AZ has developed its own concepts and chess positions, enabling flexibility and creativity in its strategies.

## 2. What Are Concepts?

### 2.1. Working Definition.

There are several possible definitions of a concept (9), varying from a human-understandable high-level feature (10) to an abstract idea (11). In this work, we define a concept as a unit of knowledge. This definition has two key properties we focus on. First, a concept contains knowledge, i.e. information that is useful. In the context of machine learning, we take this to mean that the knowledge can be used to solve a task. For example, consider the concept of a beak. We can teach the concept of a beak to another person or AI system. If the person grasps the beak concept, they can use the concept to identify beaks in birds. The second part of the definition is that a unit implies minimality; it is concise and irrelevant information has been removed.

There are many ways to operationalize this definition and we choose one of them: demonstrating that a concept can be transferred to another agent to help them solve a task. This setup entails that the concept is self-contained and useful for the task.

### 2.2. Mathematical Representation of a Concept.

How do we represent concepts? We leverage the rich literature that assumes concepts are linearly encoded in the latent space of a neural network (12–17). The latent space refers to the space spanned by postactivation features of a neural network. Although our assumption of linearity is strong, it has a surprising amount of empirical support: Linear probing and related techniques have successfully extracted a wide range of complex concepts from neural networks across multiple domains (12–17). Although we may overlook concepts with nonlinear representations, we show that we can find useful concepts for our goal using purely linear representations.

### 2.3. Concept in Reinforcement Learning.

What types of concepts do we aim to discover in the context of Reinforcement Learning (RL)? We aim to discover concepts that give rise to a plan, where a plan is a deliberate sequence of actions optimizing for one or more relevant concepts. We take deliberate to mean that there is an underlying reason. More concretely, we assume a plan is motivated by one or more concepts. Although the terminal goal of a plan is the same across states—maximizing the outcome (win or draw)—plans in a specific state will have more context-specific instrumental goals along the way, such as, capturing a particular piece in an advantageous position, or maximizing one’s board control. We assume that plans in similar contexts will share similar instrumental goals, and thus give rise to similar concepts.

## 3. Methods: Discovering Concepts

Our method can be summarized into the following steps: 1) excavating vectors that represent concepts in AZ’s latent space using convex optimization, 2) filtering the concepts based on teachability (whether the concept is transferable to another AI agent) and novelty (whether the concept contains information that is not present in human games). The resulting set of concept vectors is then used to generate concept prototypes (chess puzzle—solution pairs), which are presented to human experts (top chess grandmasters) for final validation.

### 3.1. Excavating Concept Vectors.

To find concepts, we develop a method as 1) we want an unsupervised approach to find new concepts, 2) AZ’s input is a mix of binary and real-valued inputs and many interpretability techniques require continuous inputs (e.g., saliency maps typically take as input continuous values and are generally not suitable for binary values), and 3) we want to develop an interpretability tool to analyze both parts of AZ’s machinery—the policy-value network and MCTS. Leveraging both the network and MCTS is crucial, since each component plays a different yet important role in deciding the optimal action (see *SI Appendix*, Fig. S2 for more detail).

We formulate concept discovery as a convex optimization problem. Using a convex optimization framework is not new; many existing methods for finding concept vectors, such as nonnegative matrix formulation, can often be approximated as a convex optimization problem (18). For each concept vector we want to find, we formulate a separate convex optimization problem. We define a concept as a unit of knowledge. Minimality is achieved by encouraging sparsity (19) through the $L_{1}$ norm

[1] $$\begin{array}{cc} & \min\|v_{c,l}{\|}_{1}\\ & \;\text{such that concept constraints hold}, \end{array}$$

where $v_{c,l}\in R^{d_{l}}$ is a vector that lives in latent space of layer l to represent concept c, and $d_{l}$ is the dimension of layer l.

We are free to choose the concept constraints depending on the concept we wish to recover. Denote the activations at layer l for a position by $z_{l}$. In a supervised setting, we may require $v_{c,l}^{\;⊤}z_{l}\geq 0$, for each of many positions where we know the concept is present. Each position then contributes its own inequality constraint to Eq. **1**. Such static concepts are defined to be found in a single state (or chess position). We use supervised static concepts only to validate our method, with more details in *SI Appendix*.

Different concept constraints are needed for *concept discovery without human labels*. Our framework only aims to discover dynamic concepts, which are found in a sequence of states: here, a sequence of positions from a chess game. Below, we outline how MCTS rollout statistics can be used to set up the constraints in Eq. **1** for unsupervised concept discovery.

#### 3.1.1. Concept constraints for dynamic concepts.

The statistics of AZ’s MCTS search tree are used to find candidates for meaningful sequences of states—sequences where we may expect to find dynamic concepts. MCTS generates a tree of possible moves and subsequent responses for a given start state, i.e. a chess position $x_{0}$; see ref. 20. The exact details are not essential to understand for our procedure; what matters is that AZ chooses a rollout (i.e., a sequence of potential moves and corresponding states) $X_{\leq T}^{+}=\left(x_{1}^{+},x_{2}^{+},x_{3}^{+},\ldots ,x_{T}^{+}\right)$, where T is the maximum depth of the rollout, which terminates in the most favorable state according to AZ. We contrast this optimal rollout $X_{\leq T}^{+}$ with a subpar rollout $X_{\leq T}^{-}$, which is defined as a path in the MCTS search tree that is suboptimal, according to the value estimate or visit count in MCTS. The preferential order of the rollouts gives us a mechanism to set up various inequality constraints for Eq. **1**.

The mathematical reason for AZ choosing $X_{\leq T}^{+}$ over $X_{\leq T}^{-}$ is that it is a continuation where at each state the player maximizes their action-value estimate at each move. There may be *many* conceptual explanations why AZ chooses $X_{\leq T}^{+}$ over $X_{\leq T}^{-}$. However, we can broadly classify these explanations into the following three scenarios:

1. Active planning $X_{\leq T}^{+}$ increases the presence of a concept c. For example, the rollout $X_{\leq T}^{+}$ may increase the concept of piece activity.
2. Prophylactic planning $X_{\leq T}^{+}$ avoids increasing the presence of a concept c. An example may be that the plan in $X_{\leq T}^{+}$ avoids losing a piece.
3. Random $X_{\leq T}^{+}$ is arbitrarily chosen above the $X_{\leq T}^{-}$, as all concepts are equally present in both rollouts and the value estimates of the final states are approximately equal.

We are interested in turning scenarios 1 and 2 into inequality constraints for Eq. **1**. Scenario 3 can be filtered out by ignoring two rollouts $X_{\leq T}^{+}$ and $X_{\leq T}^{-}$ when they have similar value estimates and visit counts in the MCTS statistics.

We derive our concept constraints on the vector $v_{c,l}$ by contrasting the chosen rollout $X_{\leq T}^{+}$ and the subpar rollout $X_{\leq T}^{-}$. We denote the activations at layer l at depth t by $z_{t,l}^{+}$ and $z_{t,l}^{-}$ for positive and negative examples, respectively. Details on extracting the activations can be found in *SI Appendix*, Fig. S2. The convex optimization goal is to learn a sparse vector $v_{c,l}$ that represents a concept c. We hypothesize that the inner product $v_{c,l}^{\;⊤}\;z_{t,l}^{+}$ is higher^¶^ for activations from optimal rollout (the set where the concept is present) than for activations from the suboptimal rollout (the set where the concept is absent). Therefore, a pair of positive and negative rollouts gives rise to the following convex optimization problem

[2] $$\begin{array}{cc} \min & \;\|v_{c,l}{\|}_{1}\\ \;\text{such that} & \;v_{c,l}^{\;⊤}\;z_{t,l}^{+}\geq v_{c,l}^{\;⊤}\;z_{t,l}^{-}\;\;\text{for all}\;t\leq T, \end{array}$$

for scenario 1, with the inequality reversed for scenario 2. If the concept is relevant only for the playing side, we define Eq. **2** to add inequality constraints for every second t.

**Fig. 3.**
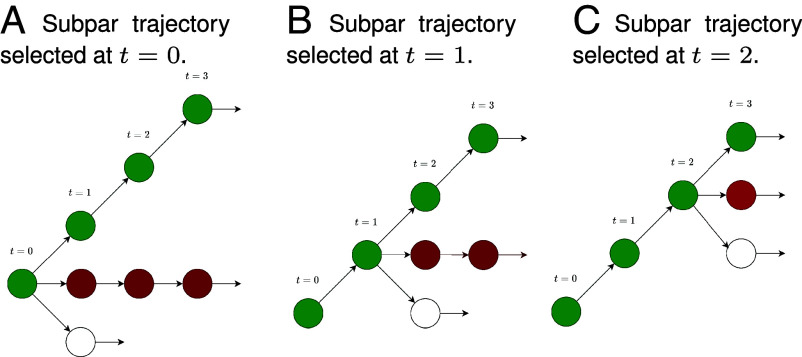
Contrasting the optimal rollout with subpar MCTS rollouts at different time steps. The green rollout shows the optimal rollout, and the red rollouts depict subpar trajectories. Each subfigure shows the subpar trajectory selected at different timesteps – (*A*) shows t=0, (*B*) shows t=1, and (*C*) shows t=2. We include each of these pairs in the concept constraints.

We extend this idea by contrasting the optimal trajectory with multiple subpar trajectories across different MCTS depths, as illustrated in Fig. 3. On the *Left* side of Fig. 3, we find the optimal and subpar trajectories at the initial chess position, t=0. However, we can also use the MCTS statistics (value estimate and visit count) to find subpar trajectories at t=1 (shown in the *Middle*) and t=2 (shown on the *Right*). The idea behind using multiple subpar trajectories is to further reduce the solution space with the goal of reducing noise in $v_{c,l}$ (thereby increasing the likelihood that we find a meaningful concept) and decreasing the likelihood of learning a polysemantic concept vector.

Let j denote the time step at which the suboptimal trajectory starts. We extend Eq. **2** to find the dynamic concept as follows:

[3] $$\begin{array}{cc} \min & \;\|v_{c,l}{\|}_{1}\\ \;\text{such that} & \;v_{c,l}^{\;⊤}\;z_{t,l}^{+}\geq v_{c,l}^{\;⊤}\;z_{t,l,j}^{-}\;\;\text{for all}\;t\leq T,j\leq \tilde{T}, \end{array}$$

where $\tilde{T}$ denotes the maximum depth at which we find suboptimal rollouts. $\tilde{T}=3$ in Fig. 3. In general, we set $\tilde{T}=T-5$ to ensure the rollout is sufficiently deep. Details on how T is set can be found in *SI Appendix*, Fig. S6.

#### 3.1.2. Choice of layers.

In the field of interpretability, deciding which layers to analyze is an open discussion. In this work, we want to determine which layers are most likely to contain new concepts. Taking a step back, the most information-rich latent spaces are likely those where the information contained in AZ games exceeds the information contained in human games. To this end, we contrast the span of AZ games to that of human games, through the lens of the AZ network, to determine which activation spaces may contain new concepts in AZ’s games compared to human games.

Let $Z_{l}^{a}$ denote a matrix where we stack the latent representations in layer l of chess positions sampled from AZ’s games. Each row represents a chess position, and each column represents a dimension in latent space in layer l. Similarly, we define $Z_{l}^{h}$ as a matrix of chess positions sampled from grandmaster games. The number of the basis vectors (i.e., the ranks of $Z_{l}^{h}$ and $Z_{l}^{a}$) estimates the size of the span of the latent representations of the games—informally, we can think of it as a proxy for the number of concepts.

Table 1 shows the ranks for $Z_{l}^{h}$ and $Z_{l}^{a}$ for different layers. We focus on the final layers in the architecture as these tend to be more complex concepts (13, 21) and are more likely to influence the policy.

We include the rank of the input as a reference. Differences in input rank may not be meaningful, e.g. AZ has longer games than humans, which may not contain informative concepts. However, the higher variance of position types may lead to a larger input rank, and therefore a larger rank in other layers. However, we observe that this is not the case. The rank of AZ’s games is smaller, and therefore we can assume that the differences in rank are due to other factors driving the observed difference.

In Table 1, Max. shows the maximum possible rank for each layer. In our analysis, we used 17,184 positions to ensure that the number of samples does not impact the rank estimation, as 17,184 is larger than the number of dimensions in each layer. Therefore, the maximum is equal to the number of neurons in each layer.

AZ games’ rank is higher than the human games’ rank at layers 19 (final layer in main bottleneck) and 23 (policy layer), suggesting that there may be concepts present in AZ games that are not in human games. Therefore, we focus on finding new concepts in these layers.

**Table 1.** Rank of latent representation of Human Games and AZ’s Games

	Input	Layer 19	Layer 20	Layer 21	Layer 23
Human	730	7,857	64	86	6,544
AZ	728	8,269	64	88	6,771
Max.	7,616	16,384	64	256	16,384

Max denotes the maximum theoretical rank.

### 3.2. Filtering Concepts.

Our approach provides many concept vectors, some or many of which represent known concepts or nongeneralizable concepts (i.e., only applicable to a single chess position). In this section, we describe how we further filter concepts to ensure that they are useful (transferable) and novel.

#### 3.2.1. Teachability.

Recall that we defined a concept as a unit of knowledge. A key aspect is that a concept is teachable to another AI agent or person, who can apply the concept to solve an unseen task. To ensure our concepts are teachable, we use teachability as a selection criterion for the final concepts. The idea is simple:

**Step 1: Prototypes of a concept**. Given a concept **v**_*c,l*_, find positions that epitomize the concept.
**Step 2: Baseline agent**. Find an agent that does not know the concept, using prototypical positions.
**Step 3: Teach**. Teach the agent the concept using concept prototypes.
**Step 4: Evaluate**. Evaluate the agent’s performance on unseen concept prototypes.

If a concept is teachable, we expect the agent’s performance to improve between the first and third steps. We use a similar process to evaluate when we evaluate our approach with humans.

##### 3.2.1.1. Selecting prototypes for teaching baseline agents.

In Step 2, we use AZ as a teacher to supervise a student network on a set of chess positions called “prototypes.” Prototypes are chess positions that exemplify the use of a concept.

For each candidate concept c, we have a concept vector $v_{c,l}$. We want chess positions x from a dataset X that epitomizes the concept c. We find these prototypes by finding chess positions for which the convex optimization constraints for $v_{c,l}$ hold.

For dynamic concepts, we find chess positions using the MCTS statistics. For each chess position x∈X, we ran MCTS. Next, we found the chosen rollout $X^{+}$ (and the corresponding latent representations $Z^{+}$) and a subpar rollout $X^{-}$ (and the corresponding latent representations $Z^{-}$) as in the original concept discovery convex optimization formulation. For a prototype $x_{i}\in X^{\mathrm{proto}}$, we require that $v_{c,l}^{\;⊤}\;z_{i,t}^{+}\geq v_{c,l}^{\;⊤}z_{i,t}^{-}$ for all t≤T. All concept prototypes are selected because the same concept vector motivates the plan in the optimal rollout. Furthermore, $v_{c,l}$ ties together a unifying plan because it was learned from AZ games where similar plans (encoded in the latent representations of the sequence of positions) were executed.

##### 3.2.1.2. Selecting a baseline agent.

For the student network, we want to find an agent that does not know the concept but does understand chess. As chess is a complex game, we cannot train an agent from scratch (using only the curriculum). Instead, we select a training “checkpoint” of AZ (i.e., the model parameters at a specific point during training) and estimate the model’s knowledge of the concept using the empirical overlap of the neural network policies:

[4] $$\begin{array}{cc} & \;\text{overlap}(\pi^{s},\pi^{t})\\ & \;=\frac{1}{|X_{\mathrm{test}}|}\sum_{x_{i}\in X_{\mathrm{test}}}\;\text{Ubbmmn1}[\;\text{argmax}\left(\pi^{s}\left(x_{i}\right)\right)=\;\text{argmax}\left(\pi^{t}\left(x_{i}\right)\right)]\;. \end{array}$$

Here, $\pi^{s}$ and $\pi^{t}$ are the student and teacher neural network policy heads, which output a probability distribution over the possible actions The overlap measures how often the teacher and student agree on the best move. We select the student as the latest checkpoint for which the top-1 policy overlap is less than 0.2.

##### 3.2.1.3. Teaching and measuring learning.

**Fig. 4.**
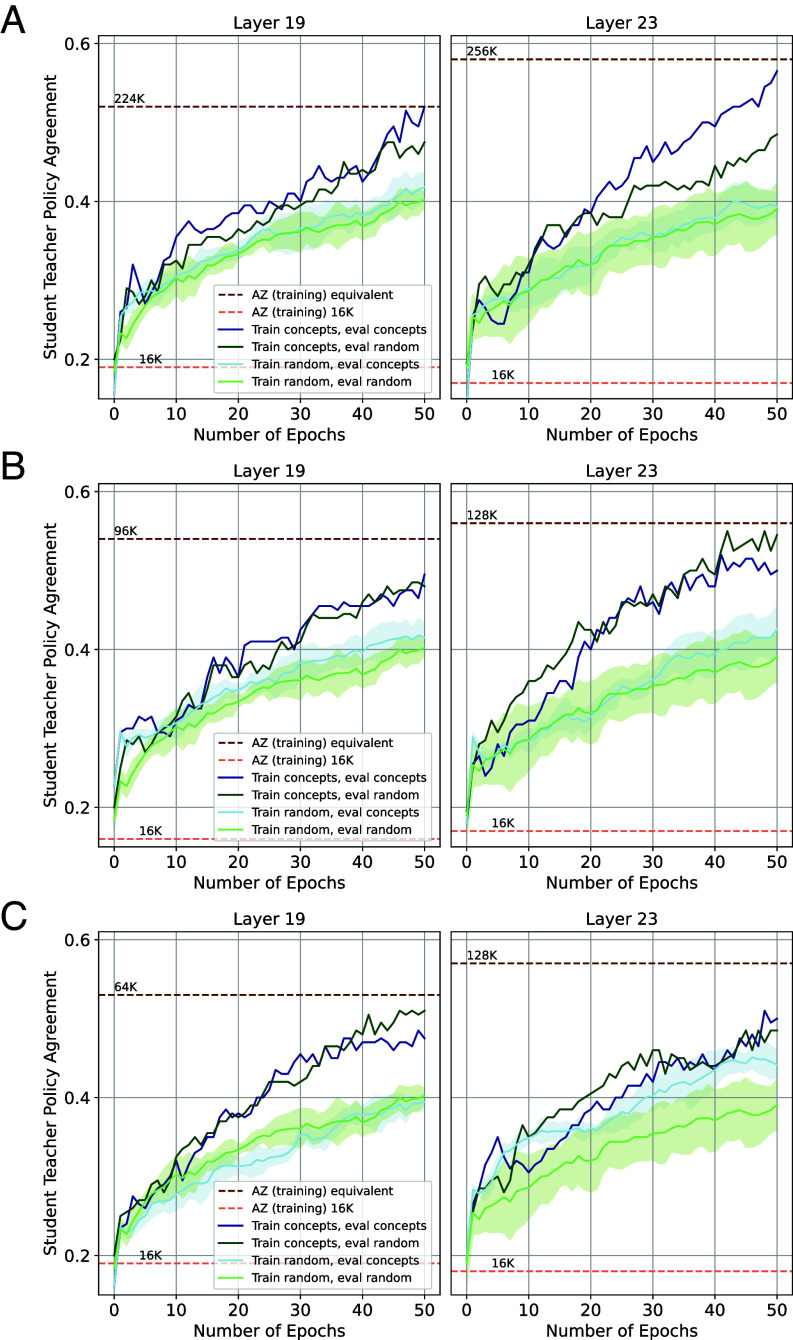
Teachability: AZ Concepts. Each row shows the training curves of a different concept. The y-axis shows how often the student and teacher select the same move (normalized version of Eq. **4**), and the x-axis shows a training epoch for Eq. **5**. For each line, the 95% CI is shown. The dark dotted lines show the level of a training checkpoint at which AZ obtains the same level on the concept set as our student. Each row originates from a different concept found in layer 19 (*Left*) and in layer 23 (*Right*).

Not all concepts are useful to improve the level of play of an agent, and therefore, we measure the effect of teaching an agent a concept. We can split $X_{\mathrm{proto}}$ into a train set $X_{\mathrm{train}}$ and a test set $X_{\mathrm{test}}$ – the allocation of prototypes to each set is random. We adapt the student using the loss function:

[5] $$\mathrm{loss}\left(\pi^{s}\right)=\sum_{x_{i}\in X_{\mathrm{train}}}\mathrm{KL}(\pi^{t}\left(x_{i}\right),\pi^{s}\left(x_{i}\right))$$

which encourages the student’s policy to match the teacher’s policy on the training prototypes. The loss in Eq. **5** is minimized with gradient descent over a number of epochs (corresponding to Fig. 4). To determine whether the student has acquired new knowledge, we evaluate the student’s performance on the test set prototypes ($X_{\mathrm{test}}$) by estimating how often the student and teacher select the same top-1 move following Eq. **4**.

##### 3.2.1.4. Baselines.

Teaching using any curriculum may improve students’ performance on the task due to the difference in strength between the teacher and student. To differentiate general learning from concept-specific learning, we compare the student’s performance when taught using concept prototypes versus chess positions randomly sampled from AZ’s games (but with meaningful plans). We sample the chess positions from AZ’s games instead of human games for two reasons: 1) AZ’s games tend to be of a higher quality than human games (as AZ has a higher Elo rating), and 2) the data is closer to AZ’s natural training data (avoiding any confounding effects due out of distribution data).

##### 3.2.1.5. Results.

Fig. 4 takes the AZ checkpoint after 16 K training iterations and shows the student’s performance in four settings:

1. student trained on concept c and evaluated on concept c (dark blue line);
2. student trained on concept c and evaluated on random data from AZ’s games (dark green line);
3. student trained on random data and evaluated on concept c (light blue line); and
4. student trained on random data and evaluated on random data (light green line).

When teaching a student with concept-specific prototypes, the student improves its performance on a random set of prototypes (dark green line) and on concept-specific prototypes (dark blue line), compared to a curriculum of randomly sampled positions. When a student was taught with randomly sampled chess positions, labeled with optimal play, it improved their performance significantly less (light lines) than when it was taught with concept-specific prototypes (dark lines). Naturally, the student learns quicker when taught with concept-specific prototypes (dark blue line) than random prototypes (light blue line). We also observe that concepts can be taught efficiently. The student’s performance after training for 50 epochs on a small set of prototypes would have taken 10 K to 250 K training iterations using self-play (dark dotted line). Recall that the student is evaluated on a held-out test set, ruling out the possibility that the student network memorized the chess positions.

We select the student to be the latest checkpoint for which the top-1 policy overlap is less than 0.2, resulting in 97.6% of the concepts being filtered out.

#### 3.2.2. Novelty.

There are different ways to ensure the novelty of concepts. We take two simple approaches: 1) ensure concepts are learned during the later stages of AZ’s training and 2) filter concepts based on a novelty metric.

##### 3.2.2.1. Require concepts to be learned during a late stage in AZ’s training.

To find complex and potentially novel chess concepts, we leverage AZ’s training history. We use two versions of AZ that differ in strength by 75 Elo points.^#^ To find interesting chess positions, we run-through AlphaZero’s games and select chess positions where the two versions of AZ disagree on the best move according to their policies, which could be seen as a form of contrasting decoding (22). We focus on finding chess concepts in the resulting set of positions.

##### 3.2.2.2. Setup to measure novelty.

While the previous section ensures that the concepts that emerge in the final stages of training in AZ are complex by construction, it does not ensure that the concepts are novel to humans. There is a level of subjectivity involved when evaluating the novelty of chess concepts from a human point of view, as any potentially newly discovered complex concept may be closely related to a number of known, more general chess concepts (e.g. material sacrifice, dynamic compensation, long-term structural weakness). However, even if related in this way, these concepts may still be novel insofar as they are contextualized and instantiated within certain types of piece configurations and patterns on the chess board, which come with a unique set of considerations that influence how a position is conventionally to be approached. Moreover, any description of a concept in natural language will inevitably resemble existing concepts, as words are inherently tied to preexisting ideas; instead, a truly novel concept may require a new corresponding term. Last, even if descriptions could correctly encompass a concept, our method does not provide a written description. It is difficult to qualitatively establish novelty in the absence of a detailed language description.

We aim to sidestep the more subjective pitfalls above by focusing on a more quantitative and objective measure, aiming to determine whether the concepts arise in AZ’s games but not in human games. Leveraging the fact that concepts are represented in the latent space as a vector, we can compare the vector space of AZ games to that of human games. Recall from A.2 that $Z_{l}^{a}$ and $Z_{l}^{h}$ are matrices containing the stacked latent representations of AZ’s and human games. Using the latent representations, 1) we first get evidence that AZ’s game is likely to contain new concepts using a rank experiment, and then 2) measure the novelty of a concept by regressing concepts onto AZ’s games vector space and human games vector space, based on which we filter concepts.

##### 3.2.2.3. Novelty scores for filtering.

We define the novelty score based on how well a concept vector can be reconstructed using a set of basis vectors derived from AZ’s games. A lower reconstruction loss means that the concept is better represented by the given set of basis vectors. In other words, we look for concepts that are better explained using AZ’s language (basis vectors) than humans’ language. We define the novelty score as the difference between a concept’s reconstruction loss (Eq. **7**) when using basis vectors from humans’ game and AZ’s games. A higher score means a closer alignment with the basis vectors arising from AZ’s games.

Specifically, for $Z_{l}^{h}$ and $Z_{l}^{a}$, we find the singular value decomposition to find the basis of the space spanned by AZ’s and human games

[6] $$\begin{array}{cccc} Z_{l}^{h} & =U_{l}^{h}\;\Sigma_{l}^{h}\;V_{l}^{h⊤}, & Z_{l}^{a} & =U_{l}^{a}\;\Sigma_{l}^{a}\;V_{l}^{a⊤}, \end{array}$$

where the columns of $U_{l}^{h}$ and $U_{l}^{a}$ form an orthonormal basis for the rows of $Z_{l}^{h}$ and $Z_{l}^{a}$, respectively; $\Sigma_{l}^{h}$ and $\Sigma_{l}^{a}$ are the singular value matrices; and the columns of $V_{l}^{h⊤}$ and $V_{l}^{a⊤}$ form the orthonormal basis for the columns of $Z_{l}^{h}$ and $Z_{l}^{a}$ respectively.

The novelty score of concept vector $v_{c,l}$ is defined as

[7] $$\min_{\beta_{l}}{∥v_{c,l}-\sum_{i=1}^{k}\beta_{i,l}u_{i,l}^{h}∥}^{2}-\min_{\gamma_{l}}{∥v_{c,l}-\sum_{i=1}^{k}\gamma_{i,l}u_{i,l}^{a}∥}^{2},$$

where $\beta_{i,l}$, $\gamma_{i,l}$ are coefficients estimated using linear regression, $u_{i,l}^{a}$ and $u_{i,l}^{h}$ are columns of $U_{l}^{a}$ and $U_{l}^{h}$, respectively, and k is the number of basis vectors used. We do not set k as the rank of the matrix because the rank differs for $Z_{l}^{a}$ and $Z_{l}^{h}$, and doing so would favor the matrix with the largest rank. Instead, we estimate Eq. **7** for various values for k.

**Fig. 5.**
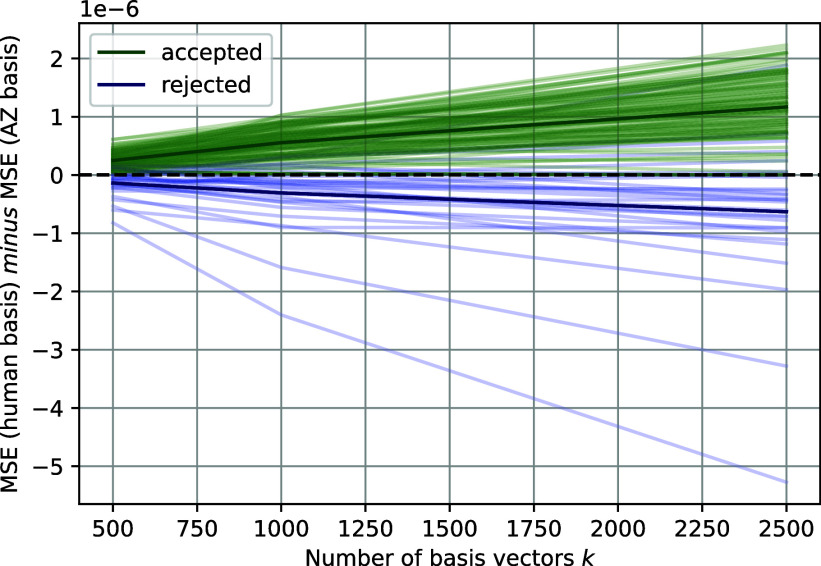
Filtering concepts based on novelty scores. Concepts for which the reconstruction error using AZ’s basis vectors is less than the reconstruction error using the human game’s basis vectors for every k are accepted (not filtered). The darker green and blue lines show the average over the accepted and rejected concepts.

Fig. 5 shows the novelty scores for 120 concepts found in layer 19. We accept concepts for which the reconstruction error using AZ’s basis vectors is less than that of the human game’s basis vectors for every k. The light green lines denote the novelty scores for the concepts we accept, and the light blue lines denote the novelty scores for the concepts we reject.

Of the concepts remaining after teachability-based filtering, we remove a further 27.1% using the novelty metric.

## 4. Human Evaluation

We investigate whether top chess grandmasters can successfully learn and apply the new concepts after being exposed to a small set of concept prototypes by expanding H. Learning from prototypes is similar to the established chess teaching practice where students are presented with puzzles corresponding to a specific theme (e.g., opening, piece sacrifices, etc.), and solving the puzzles (i.e., finding the correct next moves) allows the student to learn a new idea and improve their ability (23). Henceforth, we use puzzles to denote prototypes and their “solutions” (AZ’s selected move).

The human evaluation follows three phases:

- Phase 1: Establishing a baseline performance. Each grandmaster is asked to solve a set of puzzles corresponding to concepts. This determines the baseline performance: the number of puzzles in which the chess grandmaster identifies the continuation correct before the learning phase.
- Phase 2: Learning from AZ’s calculations. The same puzzles from Phase 1 are shown to chess grandmasters, alongside AZ’s optimal continuations (rollouts) based on MCTS calculations for each puzzle. This approach is the simplest way of teaching while bypassing potential failure modes of explanation/description generation methods.
- Phase 3: Assessing the final performance. Grandmasters are tasked with providing solutions for a test set of unseen puzzles sampled from the same concepts as in Phase 1. We measure their accuracy on the test set and compare it to their baseline performance in Phase 1 to determine whether their performance has improved.

The train-test split of puzzles (i.e, allocation of puzzles to Phase 1 and Phase 3) is random. At no point during any phase are the grandmasters shown labels identifying the underlying concepts behind the prototypes in the batch of positions they are given. At each stage, we further asked the grandmasters to provide a summary of their thought process in free form.

We worked with four experts—grandmasters who are ranked in the top 0.004% of internationally ranked players worldwide, all with former and the current world chess championship titles. Given the complexity of each puzzle, the participants were expected to spend a considerable amount of time analyzing the chess positions in great depth. Therefore, to avoid overburdening the study participants, they were presented with four puzzles per concept, focusing on 3 to 4 discovered concepts during the study. In total, each grandmaster saw 36 to 48 chess puzzles. Due to that the participants limited time and the significant time investment per puzzle, we gave each grandmaster a different set of concepts. Our experiment design prioritizes exploring a more extensive concept set, as determining which concepts are teachable to humans beforehand is difficult.

**Table 2.** Improvements in grandmasters’ performance, showing the numbers of puzzles that the grandmasters solved correctly (according to AZ’s solution)

	Puzzles solved		
Grandmaster	Phase 1	Phase 3	↑
1	0/12	5/12	+42%
2	4/12	7/12	+25%
3	3/12	5/12	+16%
4	6/16	7/15	+6%

### 4.1. Grandmaster Performance.

Overall, we find that all study participants improve between phases 1 and 3, as shown in Table 2, suggesting that the chess grandmasters were able to learn and apply their understanding of the represented AZ chess concepts.

Breaking down performance at the concept level, we found an average improvement of 0.85 more puzzles solved correctly between phases 1 and 3, with a SE of 0.12, which is statistically significant. Of the 13 concepts tested, participants improved their performance on 8 concepts, showed no change on 3, and showed a decrease in performance on 2 concepts. This suggests that the majority of concepts were learnable by humans.

#### 4.1.1. Sample size.

However, the number of participants in the sample is small, as we focus on world experts. Therefore, while our results suggest that the grandmasters may acquire the concepts, this should only be seen as a proof of concept.

#### 4.1.2. Confounders.

Further, there are other factors that may have influenced performance, such as the variability in the puzzle difficulty, teachability of a concept and external factors, such as overthinking; we elaborate on this in *SI Appendix*, Fig. S9. It is also a possible that the improved performance can be partially attributed to priming the participants to search for more complex and counterintuitive patterns (when contrasted with human play) in general, rather than their improved understanding of the specifically selected concepts in particular. However, the extent to which this may influence the results is unclear. The participants are all experienced, top-level players, who routinely search for nonobvious ideas when analyzing chess positions. As such, they are arguably less susceptible to be primed in this way compared to less experienced or proficient players.

### 4.2. Qualitative Evaluation.

#### 4.2.1. Expert comments on concepts.

**Fig. 6.**
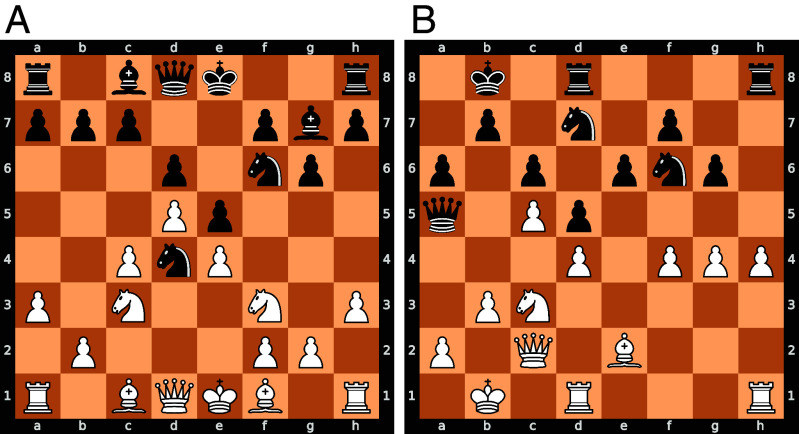
Concept Puzzles. In both puzzles, White plays a quiet move that improves the piece positioning, and prepares an unconventional long-term sacrifice. (*A*) In this puzzle, the idea is to play Bg5 to provoke the weakness h6. This weakness allows White to sacrifice the queen in subsequent continuations. (*B*) In this puzzle, the idea is to play Qd2. It one continuation, White plays b4, an unconventional pawn sacrifice that leads to an advantage.

We provide the grandmaster commentary in *SI Appendix*, Fig. S1. We refrain from providing player’s names to preserve anonymity. In general, the grandmasters appreciated the concepts, describing them as “clever” (Fig. 6), “very interesting” (*SI Appendix*, Fig. S9), and “very nice” (*SI Appendix*, Fig. S11).

Further, they found that the ideas often contained novel elements, commenting that the moves were “something new” and even “not natural” (Fig. 6 in the main text and *SI Appendix*, Fig. S9). The grandmasters found the prototypes complex—remarking that they were “very complicated—not easy to understand what to do.” Even after seeing AZ’s solutions, they remarked that it was a “very nice idea which is hard to spot” (*SI Appendix*, Fig. S15).

#### 4.2.2. Human-teachable concept.

In this section, we provide two examples of puzzles corresponding to a concept we discovered using the method described in the previous sections. Further examples of concept puzzles can be found in *SI Appendix*, Fig. S1. The descriptions of the concept and prototypes are provided by the authors. While we have made our best effort to capture the essence of these concepts, they are highly complex, and our explanations may not fully encapsulate their depth.

The concept seems to exploit tactical motifs that leverage the opponent’s weaknesses to achieve strategic and prophylactic goals.^‖^ The plan often focuses on improving the player’s piece placement. Although the concept incorporates elements of strategies that are individually known in other contexts, their combined application in these positions has been novel and therefore surprising. This was remarked by the grandmaster, who described the idea as “useful” but “not natural.” We speculate that the concept is learnable by humans, as evidenced by the grandmaster who improved their performance from 0/4 in Phase 1 to 2/4 in Phase 3, after training on this concept.

#### 4.2.3. Examples of concept prototypes.

Fig. 6 shows two puzzles provided to the participant in Phase 1. In the following text, we use this font to distinguish chess moves from normal text. The grandmaster chose 9.Be3, a natural move that follows the general principles humans use to play chess—developing pieces and controlling the center (24). Here, AZ plays the move 9.Bg5. At the surface level, the idea is to provoke the structural weakness 9...h6 before retreating to the same square suggested by the grandmaster e3. While the general idea of provoking a weakness is not new to humans, AZ’s calculations reveal an interesting and potentially novel idea:

9.Bg5 h6 10.Be3 O-O 11.Nxd4 exd4 12.Qxd4 Ng4
13.hxg4! Bxd4 14.Bxd4

AZ sacrifices the queen – a beautiful and rare tactical (i.e., for an immediate gain) motif in chess, as it contradicts established chess principles. In chess, each piece has a value, and a fundamental principles is to avoid trading more valuable pieces (i.e., the queen valued at 9 points) for less valuable pieces (a knight and bishop, together valued at 6 points). However, AZ’s queen sacrifice is strategic (i.e., for long term gain)—White continues developing their pieces after sacrificing the queen.

For the queen sacrifice, playing 9.Bg5, instead of 9.Be3 is important as it provokes a critical weakness. Due to the pawn on h6, Black’s king is vulnerable, and White is better. Without the pawn on h6, White is lost, highlighting this is the critical positional element.

Another puzzle for the same concept is shown in Fig. 6. Again, AZ has a complex yet beautiful plan:

21.Qd2 Rh7 22.h5 Rdh8 23.Rh3 gxh5
24.g5 Ng4 25.Rf1!? Nf8 26.b4!! Qxb4+
27.Ka1 Qa5 28.f5! exf5 29.Qb2 Ne6
30.Nxd5!! cxd5 31.Ra3 Qc7 32.Bxa6 White is better.

The grandmaster eloquently summarized the complexity of this move:

“21.Qd2 is a useful move as it stops Ne4 and protects f4 and can be better placed in case of b4 in the future. One curious line [given by AZ] is 21...Rh7 [22.h5 Rdh8] 23.Rh3 gxh5 24.g5 Ng4 White can just play 25.Rf1 and then focus on getting the b4 [pawn] break, which is not natural.”

Due to the king on b1, the move b4 is an unconventional idea. However, it is powerful as it allows White to gain space and open up the position under unfavorable circumstances for Black, thereby securing an advantage. This idea goes against common principles, which prioritize material and king safety.

The ideas in both positions were missed by the grandmaster. AZ’s ideas require unconventional continuations that go against common human chess principles. These observations hint at the existence of superhuman knowledge (M−H). It is perhaps not immediately clear what the exact commonality between these examples is, and what specifically makes these applications of advanced (yet more generally known and recognizable) chess principles form a distinct concept, from the perspective of AlphaZero. While we recognize that interpretability remains an open challenge, especially for intricate and complex concepts, here we outline one possible approach that can act as a starting point for improving our understanding of the discovered concepts—graph analysis.

#### 4.2.4. Understanding AZ’s concept using graph analysis and human-labeled concepts.

**Fig. 7.**
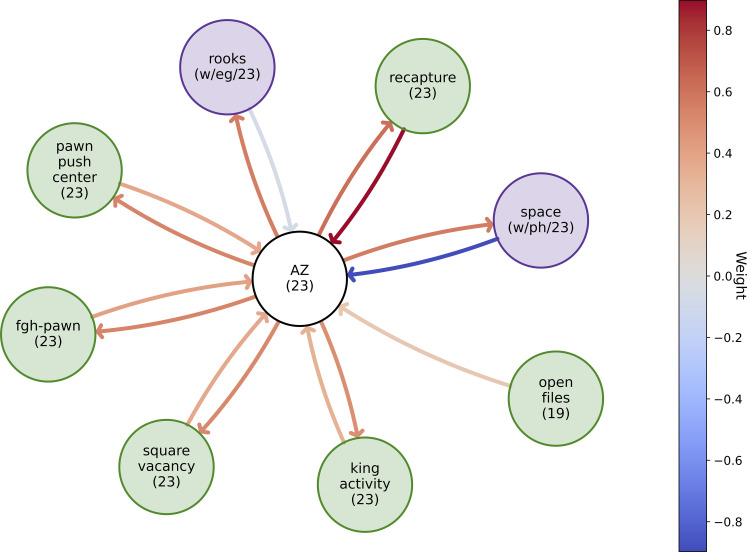
Graph of AZ’s concept in Fig. 6 and *SI Appendix*, Figs. S2, S4, and S5 between AZ’s (white), strategic (green), and Stockfish concepts (purple). The information in the parentheses means the layer in which the concept is found, and w = white, b = black, eg = endgame, mg = middlegame, ph = phased. The edge color denotes the edge weight.

We fit a graph (see *SI Appendix*, Fig. S8 for details) between concept vectors to discover relationships between existing and discovered concepts to gain further insight into the concept meaning (shown in Fig. 7). The edge weight in the graph is influenced by 1) the strength of the relation between two concepts and 2) the frequencies at which concepts co-occur.

##### 4.2.4.1. Space.

AZ’s concept has a positive outgoing edge weight with the (White-side) space concept—which is in line with Fig. 6 in the main text, *SI Appendix*, Figs. S4 and S5. In a similar vein, given that the idea is to increase space, which is “easier”/more likely if the initial value is lower, AZ has a negatively weighted incoming edge with the concept space.

##### 4.2.4.2. Recapture.

We observe positive incoming and outgoing edge weights with the recapture concept. Recall that we have dynamic concepts, which refer to a sequence of states. As such, we postulate that this connection is because the plan may be to recapture/gain material in the subsequent chess positions, as in the puzzles in Fig. 6 and *SI Appendix*, Fig. S5.

## 5. Related Work

Here, we provide a brief overview of the relevant prior work on concept-based explanations, interpretability of reinforcement learning systems, and AI for chess.

### 5.1. Concept-Based Explanations.

Concept-based methods use human interpretable high-level abstractions to provide model explanations (13, 25–30). These methods are particularly prominent in scientific and biomedical applications (31–39), and have also been studied in board game playing agents, for example in Hex (40) and Go (41).

Concepts can be derived with or without supervision. In supervised approaches, models are often probed using a labeled dataset of concept exemplars (13). When going beyond supervised concepts derived from human labels (21, 42, 43), concepts may be expressed via example sets of data points (42, 43) or by generating new data (21).

However, concept-based explanations have limitations. In supervised concept-based methods, different probe datasets may lead to inconsistent results (44). Further, more generally, there are intrinsic limitations to the linearity assumption (45, 46), establishing causal links with model predictions (10, 47, 48), and challenges in aligning with the human mental models of a concept (49).

### 5.2. Generating Explanations in Reinforcement Learning.

Explanations for RL methods (50–58) have different requirements compared to traditional supervised learning settings. These differences arise due to the temporal dependency between states, actions, and subsequent states, where an agent’s past, present, and future state-action sequences may relate to a long-term goal (56). Explainability methods in RL can help identify issues such as overfitting to training data, reliance on spurious correlations (59), poor out-of-distribution performance (60) and challenges in interagent dynamics (57). Moreover, these methods in RL may help provide useful counterfactuals (61, 62).

There is a need for post hoc RL interpretability methods. Currently, input saliency maps are commonly used (63–66). However, saliency maps can suffer from unfalsifiability and cognitive bias (1, 67) and lead to incorrect results (3). Moreover, while saliency maps may help give insights into individual actions in terms of the immediate inputs, they do not allow us to easily identify generalizable higher-level concepts across sets of inputs. Other extensively used methods include tree-based methods (68–74), and various techniques for analyzing agent behavior—e.g., visualizing the agent memory over trajectories (75), extracting finite-state models (76), and leveraging Markov decision processes (77, 78) for detecting subgoals or emerging structures (79).

While most prior work focuses on understanding agent policies without search, in our work, we consider discovering concepts for explaining agent decisions based on MCTS. Given that different explainability methods offer different insights into model predictions, future work should move toward more holistic, composite approaches, that bridge and combine existing techniques to give a more comprehensive and reliable set of explanations (80, 81).

### 5.3. Chess and AI.

Chess has been a test bed for AI ideas for decades. Early engines were based on human knowledge, and their superhuman strength came from their computational capacity, which allowed them to evaluate orders of magnitude more variations thanhuman chess players. The introduction of neural networks and RL-based approaches aimed to revitalize the field, which led to a surge of improvements in computer chess engines. These advances were in part inspired by the prominent results of AZ in chess and its variants (82–85), and Lc0 (86), an open-source reimplementation of the original model, is still competing at the highest level of computer chess.

As interactions with chess engines play a key role in chess players’ preparation and training, interpretability can play an important role in helping players improve their abilities. To this end, prior work has looked at piece saliency (87), tree-based explanations (88) and natural language (89, 90). Recently, natural language explanations for chess were explored in ChessGPT (91).

Using probing-based techniques, researchers found that AZ and Stockfish encode human-like concepts in their networks (12, 92). This prior investigation of concepts in AZ focused on known human concepts in the policy-value network and did not consider search, move sequences, or new concepts. However, preliminary questions have been raised regarding whether human players have been adopting AZ’s ideas (93), as some prominent motifs had been analyzed in detail in the book Game Changer (94).

## 6. Conclusion

Our research represents a step toward understanding the potential of human learning from AI. In this work, we focused on AZ—an AI model that attained a superhuman chess level through self-play, without prior knowledge or human bias. We provide evidence for the existence of superhuman knowledge in AZ using spectral analysis to show that AZ’s games encode features that are not present in human games. To extract this knowledge from AZ’s representational space, we developed a framework to uncover new chess concepts. To mitigate human bias, we use an unsupervised approach that leverages AZ’s training history to curate a set of complex chess positions. We ensured each concept is useful, by verifying that the concept can be taught to another AI agent who can apply the concept to solve a downstream task, and novel, by measuring the alignment with the basis vectors of human and AZ games. Communicating novel concepts requires the challenge of developing a common language between humans and AI. We bypass the need for this language by leveraging puzzles (i.e., prototypes) for each concept.

We collaborated with four world-top grandmasters to 1) validate the human capacity to understand and apply these concepts on an unseen test set by studying AZ’s concept prototypes and 2) improve our understanding of the differences between AZ’s and humans’ chess representation space. In our small-scale study, all grandmasters improved their performance after learning concepts compared to baseline performance. We speculate that the differences in the representation space between AZ and humans may stem from: 1) prior biases over concepts, including their perceived applicability, importance, and how they can be combined with other concepts. For example, AZ shows a reduced emphasis on factors such as material value and is more agile in switching between playing on different sides of the board. 2) a difference in the motivation and objectives when playing chess; AZ is trained to play optimally rather than competitively. Human players may leverage their opponents’ weaknesses, such as limited strength, time, or energy.

There are several aspects of the work that could be further explored. In our work, we found a subset of all possible concepts. For example, we limited our investigation to linear sparse concept vectors. However, other concepts may be discovered in the form of nonlinear vectors. Additionally, the current work focuses on finding a single concept to explain a plan. However, a plan may contain multiple concepts. As such, an interesting aspect to further explore is how these concepts relate to each other and influence the plan.

Further work could expand on the human experiment study. While our work provides a proof of concept, a larger scale study is important to draw statistical conclusions. We limited our subjects to top-ranked grandmasters as we suspected that acquiring knowledge in (M−H) requires experts at the frontier of human knowledge. A different pool may require different ways of teaching rather than using prototypes to aid learning.

Moreover, it would be interesting to further explore the optimal conditions for humans to learn novel concepts. We allotted a fixed time budget for grandmasters to assimilate the concepts due to time availability of the world class players. However, it is plausible that an unlimited time budget could yield more profound and more intricate insights. In our research, we provided grandmasters with part of AZ’s MCTS, in which the rollouts are motivated by the concept, as an explanation for the concept. We used this approach to keep the explanations as familiar and simple as possible. Nonetheless, it would be interesting to augment this phase with an interactive component: e.g., for each puzzle, humans can actively engage with AZ by playing moves and asking AZ what its response is. This interactive element would allow humans to investigate counterfactual scenarios, allowing for a deeper understanding of why AZ did not select their solutions or approaches.

## Supplementary Material

Appendix 01 (PDF)

Dataset S01 (RTF)

## Data Availability

Some study data are available. All prototypes of concepts used in the human experiment are provided in the supporting information. Further, for specific concepts, we have provided further details in the main text (Figs. 2 and 6) and supplementary document (*SI Appendix*, section 9) in detail as well as anonymized averaged statistics of grandmaster’s evaluations (Table 2). We regret that we are not able to share AlphaZero codebase and games as this is proprietary. Further, we cannot provide further details (e.g., the solutions provided by each individual) from the human experiment due to data privacy.

## Footnotes

^*^Chess positions and moves can be interpreted as states and actions in RL.

^†^The kingside refers to the side of the board marked with a-d at the bottom.

^‡^A checkmate is when you attack the opponent’s king and there is no way for the king to escape.

^§^HY is a former women’s world champion, MVL is the former world blitz champion (blitz is a shorter time control), VK is a former world champion, GD is the current world champion.

^¶^A larger inner product corresponds to a higher cosine similarly.

^#^For reference, the expected score of a player rated 75 Elo points higher is 0.61, where 1 point is given for a win, 0.5 for a draw, and 0 for a loss. 75 Elo points is a large Elo difference, particularly at AZ’s playing strength.

^‖^In chess, tactics refer to a sequence of moves that creates immediate threats and typically result in an immediate impact on the evaluation of the position. This contrasts with strategy, which is more concerned with long-term plans and improvements, often without posing immediate threats.
