# Supplementary material for: Bridging the human–AI knowledge gap through concept discovery and transfer in AlphaZero
Source: Proc Natl Acad Sci U S A. 2025 Mar 26;122(13):e2406675122. doi: 10.1073/pnas.2406675122 (PMC12002201; doi:10.1073/pnas.2406675122)
Supplement: Supplementary file 1 — Appendix 01 (PDF) [file pnas.2406675122.sapp.pdf]

PNAS
www.pnas.org

Supplementary Information for

Bridging the Human–AI Knowledge Gap through Concept Discovery and Transfer in AlphaZero

Lisa Schut, Nenad Tomašev, Tom McGrath, Demis Hassabis, Ulrich Paquet and Been Kim

Corresponding Author: Lisa Schut
E-mail: schut@robots.ox.ac.uk

This PDF file includes:

Figs. S1 to S24
Tables S1 to S10
SI References

Contents

1 Human experiments: more concept puzzle examples **2**
A Concept example: positive knowledge transfer . . . 2
B Concept example: unsuccessful learning . . . 5
C Differences between humans and AZ . . . 13

2 Background: Chess in AZ and Humans **14**
A AZ policy value network . . . 14
B Background: how humans and AZ play chess . . . 14

3 Concept constraints for static concepts **15**

4 Method evaluation **16**
A Evaluation of the proposed convex optimisation framework for finding concept vectors . . . 16
B Do the concept constraints hold for a test dataset? . . . 17
C How many data points do we need to learn a concept? . . . 17
C.1 Does amplifying concept vectors increase concept-related behavior? . . . 18

5 Extra results **19**
A Concept constraint satisfaction . . . 19

6 Further details: convex optimisation formulation for concepts **22**
A Dynamic concept hyperparameters . . . 22
B Datasets . . . 23
C Convex formulation for different datasets . . . 23
D Beta hyperparameter tuning . . . 25

7 Teachability implementation **25**

8 Graph analysis **25**

9 Human experiments **26**

1. Human experiments: more concept puzzle examples

In this section, we provide more examples of concept puzzles, along with the grandmasters' analysis of the puzzles and the provided AZ suggestions. The puzzles are plotted using the `chess` python package (1).

A. Concept example: positive knowledge transfer. Here, we delve further into the concept provided in the main text. The concept possesses strategic and prophylactic characteristics, involving plans that improve the player's piece placement while restricting the opponent's activity. This concept contains an additional element of exploiting tactical motifs and weaknesses, combining strategic and tactical play. We speculate that the concept is learnable to humans, as the grandmaster who trained on the concept puzzles improved their performance between Phases 1 and 3. In Phase 1, they did not identify AZ's plan in any concept puzzle (0/4), whereas they successfully identified AZ's plan in 2/4 of the concept puzzles in Phase 3.

Figures S1 and S3 show two of the puzzles provided to a grandmaster in Phase 1. In Figure S1, AZ plays the move `9.Bg5`; the idea is to provoke `9...h6` before retreating to the square `e3`, thereby inducing a structural weakness. Instead, the grandmaster chose `9.Be3`, a natural move to develop the bishop. After seeing AZ's calculations, the grandmaster acknowledged the strength of provoking this weakness:

> "`9.Be3` allows Black the clever option of playing `[9...Nxf3 10.Qxf3] Nh5` [as provided by AZ, followed by] f5 ... but `9.Bg5` is clever as it provokes h6 after which f5 is not great and also [the pawn on] h6 serves as a hook for the pawn advance `g4-g5`. Blacks' plan of stopping `c5` with `b6` and playing `h4-h5` is interesting too but with `g4` anyways White manages to open lines so I would prefer White there."

The idea of playing provocative bishop moves to induce pawn weaknesses is not new and arises in human play. However, the interesting and potentially novel element here lies in the planned strategic queen sacrifice that emerges in one of the critical lines in the MCTS calculations. Consider one such critical continuation:

`9.Bg5 h6 10.Be3 O-O 11.Nxd4 exd4 12.Qxd4 Ng4 13.hxg4!`

Queen sacrifices are among the most beautiful (and rare) tactical motifs in chess, as they go against the established chess principles – do not trade more valuable pieces (i.e., the queen) for less valuable pieces (a knight and bishop). However, here AZ's queen sacrifice is strategic – after sacrificing the queen, White continues developing their pieces. The line continues `13...Bxd4 14.Bxd4`, as shown in the left of Figure S2. Here, due to the pawn on `h6`, Black's king is vulnerable, and White

Fig. S1. Puzzle of shown in Phase 1. White is to play.

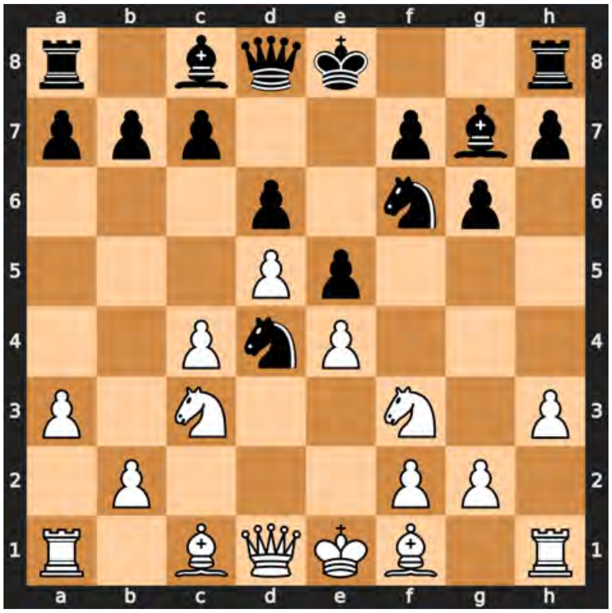

AZ's calculations: `9.Bg5 (9. Be3 Nxf3+ (9...O-O 10.Nxd4 exd4 11.Bxd4 Nxe4 12.Nxe4 Qh4 13.Bxg7 Qxe4+ 14.Qe2 Qxe2+ 15.Bxe2 Kxg7 16.Kd2 Bd7) 10.Qxf3 Nh5 11.g3 O-O 12.Be2 f5 13.exf5 Bxf5 14.Qg2 Bd7 15.O-O-O a6 16.c5) 9...Nxf3+ 10.Qxf3 h6 11.Be3 b6 12.Bd3 Qe7 13.Qe2 a6 14.Qd2 Nd7 15.Bc2 h5 16.O-O-O h4 17.g4 White is slightly better`

Fig. S2. Further analysis of the puzzle in Figure S1. In both puzzles, Black is to move.

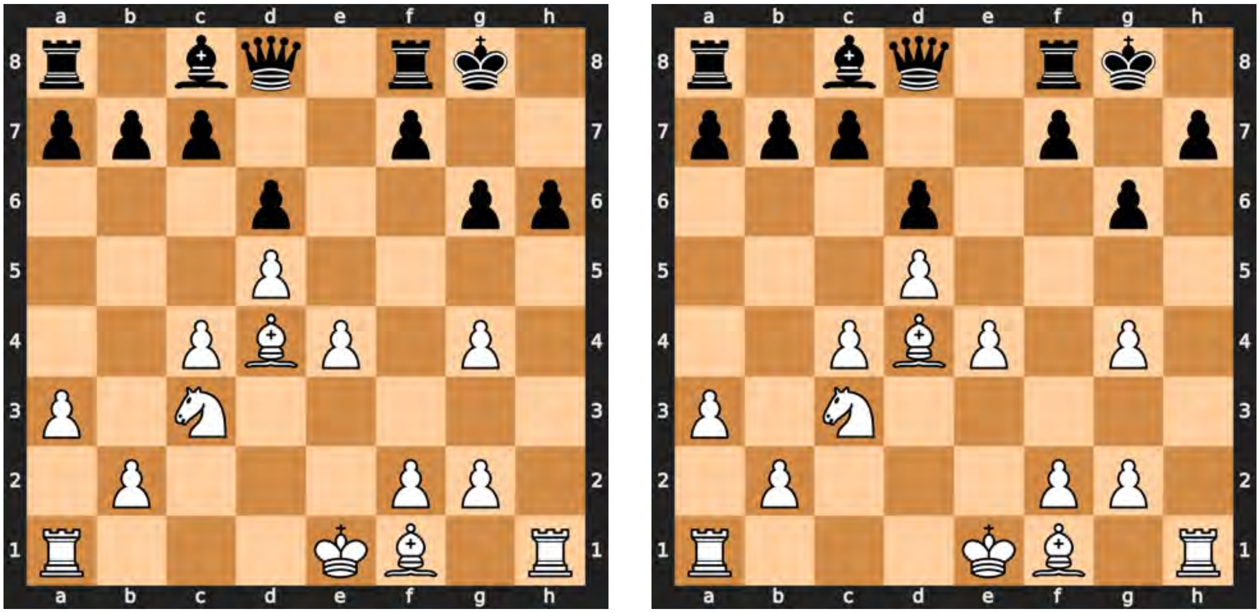

is better. Therefore, it was critical to play `9.Bg5`, rather than `9.Be3`, to make the queen sacrifice feasible by creating this weakness. The puzzle on the right of Figure S2 arises in the same line if one opts for immediate `9.Be3` instead, failing to induce `h6` first. Without the pawn on `h6`, White is lost, highlighting this is the critical positional element.

In general, some moves in the AZ's calculations are more important for the concept than others. In our convex optimisation formulation, we do not require the concept to be equally important for every chess position (see Equation 3-4). This is illustrated in the previous example, where `Bg5` is more important for the concept.

The next puzzle from the same concept, also given in Phase 1, is shown in Figure S3. This puzzle is of particular interest due to the unconventional plan of AZ – it increases space on both sides of the board, expanding with the pawn move `b4` while the king is still on `b1`. To this end, AZ initiates this plan with the prophylactic move `21.Qd2`, preventing the immediate `21...Ne4`

Fig. S3. Second puzzle shown in Phase 1. White is to move.

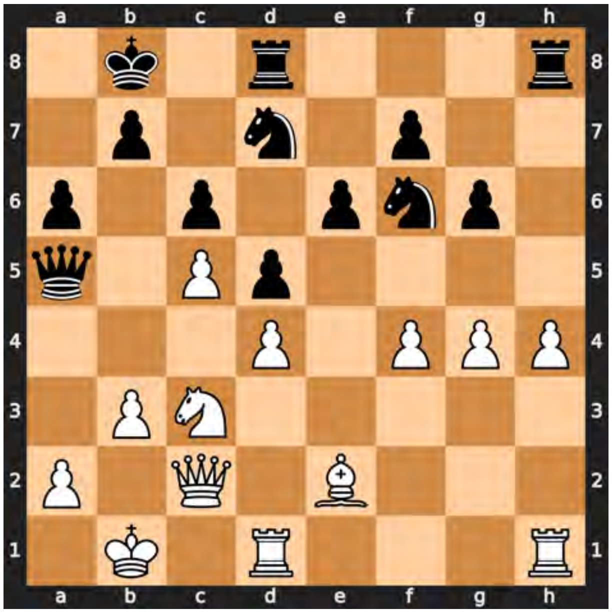

AZ's calculations: 21.Qd2 Qc7 (21...Rh7 22.h5 Rdh8 23.Rh3 Qc7 24.b4 Ne4 25.Nxe4 dxe4 26.g5 gxh5 27.a4) 22.Bf3 Rh7 23.Rh3 Rdh8 24.Rdh1 Ne8 25.Bd1 (25.b4 f6 26.Bd1 e5) White is slightly better

by Black.

Unlike AZ, the grandmaster's suggestion in this puzzle was 21.g5, with the intention of following up with 21..Nh5 22.Bxh5 Rxh5 23.Rh3 Rdh8 24.Rdh1. Upon seeing the suggested AZ line starting with 21.Qd2, however, the grandmaster study participant remarked

> "21.Qd2 is a useful move as it stops Ne4 and protects f4 and can be better placed in case of b4 in the future. One curious line [given by AZ] is 21...Rh7 [22.h5 Rdh8] 23.Rh3 gxh5 24.g5 Ng4 White can just play 25.Rf1 and then focus on getting the b4 [pawn] break, which is not natural."

Fig. S4. Expanding on the critical line in the puzzle shown in Figure S3. White has just played b4.

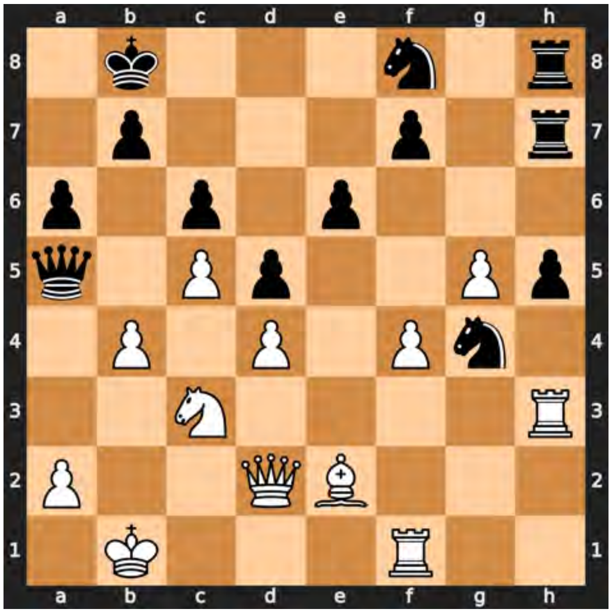

The unconventional plan of pushing the pawn to b4 with the king potentially exposed on b1 is particularly strong in this chess position (shown in Figure S4) as it allows White to gain space and open up the chess position under unfavourable

circumstances for Black, and claim an advantage. Therefore, the more general rules here are discarded based on concrete analysis. For example

```
21.Qd2 Rh7 22.h5 Rdh8 23.Rh3 gxh5 24.g5 Ng4 25.Rf1!? Nf8 26.b4!! Qxb4+ 27.Ka1 Qa5 28.f5! exf5 29.Qb2
Ne6 30.Nxd5!! cxd5 31.Ra3 Qc7 32.Bxa6 White is better.
```

In this line, we see the dynamic play of AZ: the rooks from `f1` and `h3`, switch over to the b-file to attack Black's king.

So far, the ideas in both positions were missed by the grandmaster. AZ's ideas require unconventional continuations that go against common human chess principles. Both of these observations hint at the existence of super-human knowledge $(M - H)$.

Fig. S5. In the left puzzle, White is to move. In the right puzzle, Black is to move.

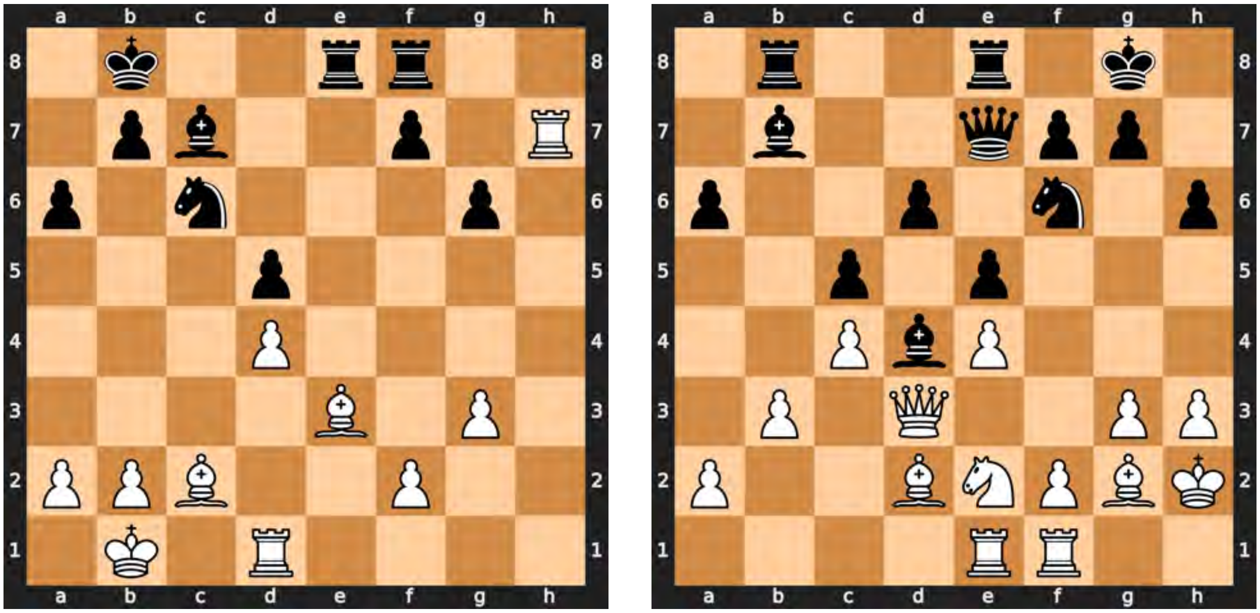

Figure S5 shows the puzzle from the same concept provided to the same grandmaster in Phase 3; Phase 3 tested whether the grandmasters had learnt the concept. As before, the puzzles underscore the concept's multifaceted attributes, encompassing its prophylactic characteristics and its integration of tactical and strategic elements.

In the puzzle (from Phase 3) shown on the left of Figure S5, the grandmaster correctly found the move suggested by AZ: `24.Bb3`, with the idea of forcing the Black rook on `e8` into a more passive position (`d8` to defend the pawn) prior to commencing activity on the other side of the board. The idea can be seen in a possible continuation:

```
24...Rd8 25.Ba4 Na5 26.Rdh1 Nc4? 27.Bh6 and White picks up the pawn on f7.
```

As in the previous puzzles, AZ uses both sides of the board to optimise piece activity.

In the puzzle, shown on the right in Figure S5, the grandmaster again found the correct idea: `22...Bc6`, with the idea of preventing the critical pawn advance `23.f4` because of:

```
23.f4 Qb7 24.Nc3 exf4 25.Rxf4, resulting in a weakened pawn structure,
```

where White cannot recapture with `25.gxf4` as `e4` is hanging. The move `22...Bc6` is both prophylactic and tactical; it prevents White from executing their plan to advance the kingside pawns while improving Black's position by activating the bishop and rook.

The overall improvement of the grandmaster on this concept suggests that they may have learnt AZ's concept, thereby expanding H with $(M - H)$.

B. Concept example: unsuccessful learning. This concept is related to gaining and playing with a space advantage with positional advantage despite less material. In this section, we provide an example of when a grandmaster found the correct move in Phase 1, but provided an incorrect (i.e., not AZ's choice) move in Phase 3. The puzzle on the left side in Figure S7 was provided to the grandmaster in Phase 1, and they correctly chose the same move as AZ: `g4`. However, while the grandmaster found the correct move, there were further finesses in AZ's calculations that the grandmaster missed during their time-constrained analysis:

> White is a pawn down, [and AZ plays the move I suggested] `g4` ... The computer plays with `h4-h5` [and] `g4` and the queen on `f2` or `g2`. White is playing on the kingside.* Black has no active moves, is playing `Rd8` or `[R]f8`, `Kh8`.

*For readers - the queenside refers to the left side marked a-d and the kingside refers to the right side of the board marked e-h.

Fig. S6. Graph of AZ's concept in Figures S1, S3 S2 and S5 between AZ's (white), strategic (green) and Stockfish concepts (purple). The information in the parentheses means the layer in which the concept is found, and w = white, b=black, eg= endgame, mg = middlegame, ph=phased. The edge color denotes the edge weight.

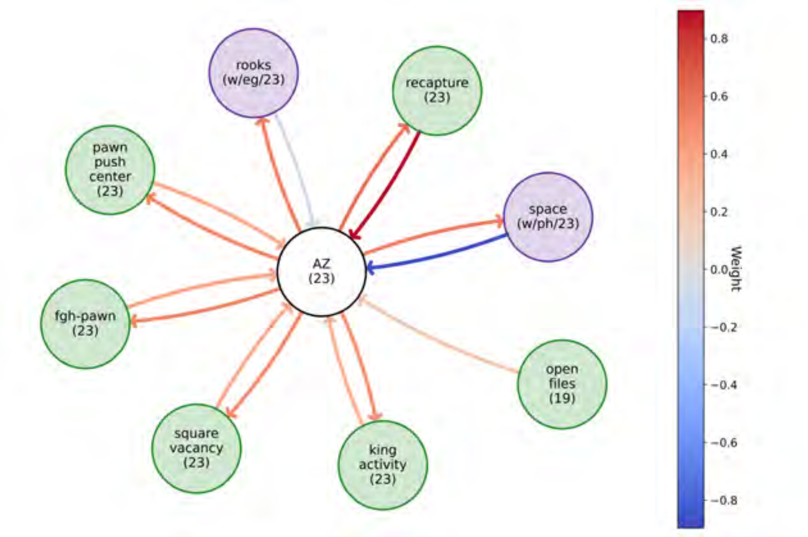

Seems convincing to me. On g6 [AZ] goes [Q]g2 which is nice, it didn't occur to me. I was mainly focused on making h4 work for White. White is not in a hurry, will at some point play g5. Compensation, zero counterplay, and AZ is acting on these premises.

Fig. S7. In both puzzles, White is to move.

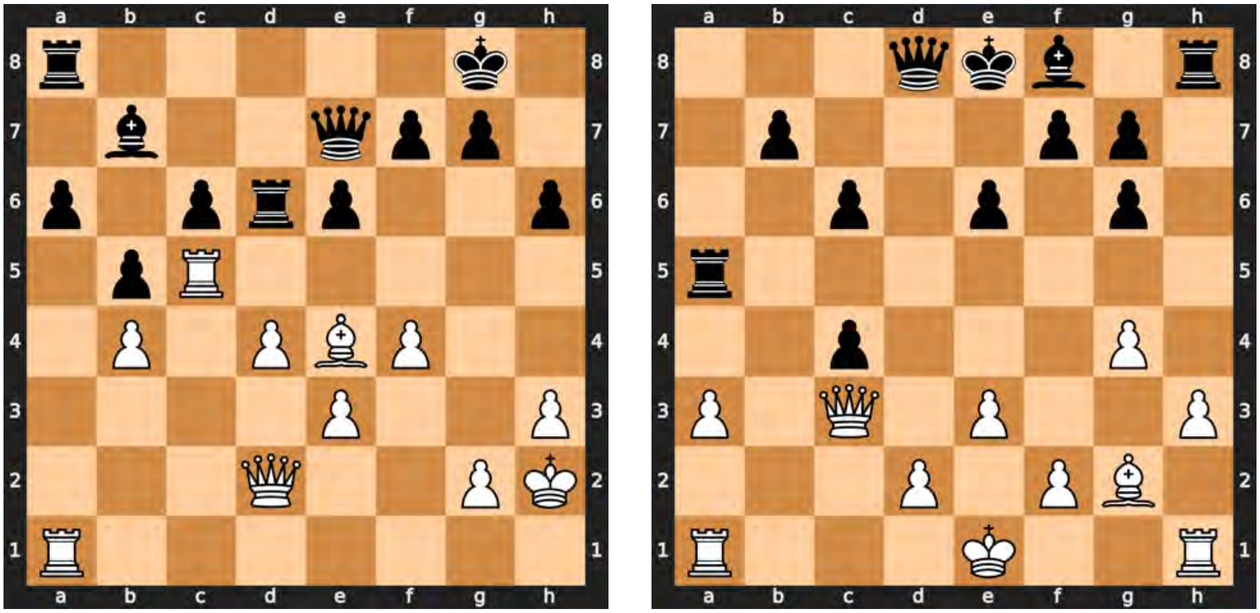

AZ's calculations: `33.g4 g6 (33...f6 34.Qg2 Rf8 35.Rf1; 33...Rad8 34.Qf2 f6 35.Rg1) 34.Qf2 Kh8 35.h4 White is better`

As remarked by the grandmaster, AZ is a pawn down, however, Black's pieces, particularly the Bishop on b7, are placed passively. Instead of prioritising regaining material, AZ focuses on improving the kingside position. While the grandmaster understood the general plan, they missed the intricate idea Qg2, and slowly advancing the g and h pawns.

The puzzle on the right in Figure S7 was provided to the grandmaster in Phase 3. Similar to the previous example, AZ focuses on space rather than recapturing material. It continues with the move 18.a4, which was rejected by the grandmaster on account of 18.Rc5, where Black tries to maintain the material advantage. However, AZ finds the rook on c5 misplaced and continues

`18.a4 Rc5 19.a5 Be7 20.a6 where after Bf6? White has 21.d4`

The idea behind the pawn advance is to weaken Black's pawn structure.

Overall, the evidence suggests that the grandmaster did not learn this concept. Here, AZ selects stronger moves due to its prioritisation of concepts (e.g., focusing on space and activity). Humans tend to prioritise these concepts differently (e.g., prioritising bringing the king to a safe location as soon as possible). This concept may be inherently difficult and require further examples to learn.

Fig. S8. Graph of AZ Concept in Figure S7 between AZ's (white), strategic (green) and Stockfish concepts (purple). The information in the parentheses is the layer number in which the concept is found. w = white, b=black, eg= endgame, mg = middlegame, ph=phased. The edge color denotes the edge weight.

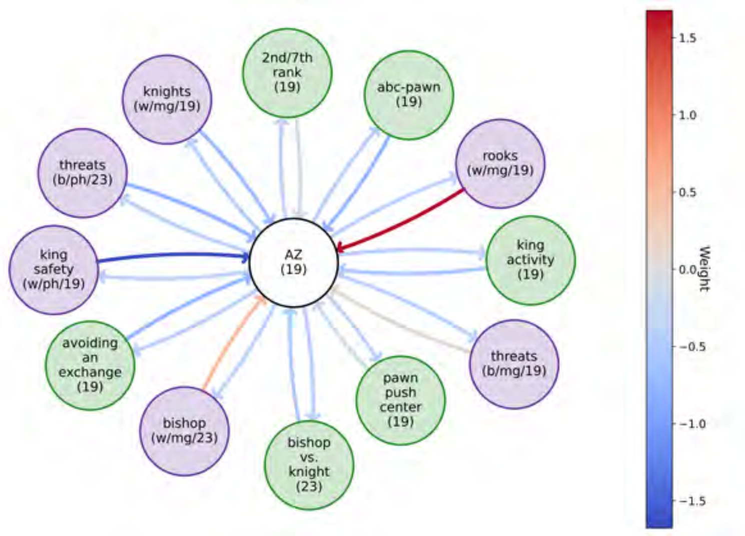

Understanding AZ's concept using graph analysis and human-labelled concepts. Figure S8 shows the relationship between AZ's concept and high-quality human-labelled concepts. The graph is dense, and we elaborate on the two concepts with the largest edge weight.

Rook. AZ's concept has an incoming positive edge with the rook (activity) concept. In the puzzles in Figure S7, we observe that white White has active rooks (the rooks on `a1` and `c5`, in the left chess position) or plans to activate the rook (the rook on `a1`, in the chess position on the right).

King. AZ's concept has an outgoing negative edge with king safety. In the chess positions in Figure S7, the king is less safe than usual. In the left chess position in Figure S7, AZ pushes forward the pawn to `g4` (and later the pawn to `h4`) around the king – thereby removing some of the king's defenders. In the right position, White does not castle to improve the king's safety but instead leaves the king in the centre.

Concept example 2: positive knowledge transfer. On a high level, this concept appears to be intrinsically related to centre control and improving piece activity. However, a more detailed analysis unveils a nuanced dimension to this concept, as AZ leverages unconventional manoeuvres to achieve these goals. The grandmaster improved performance by +2/4 between Phases 1 and 3, suggesting that this concept was not part of their existing knowledge and is human-learnable.

Figure S9 shows a concept puzzle that was shown in Phase 1. Here, AZ plays the move `5...Bf5` to control the square e4. The grandmaster chose `5...Bh5` while also considering the moves `5...Bxf3` and `5...Qf6`. After seeing the solution, the grandmaster commented

> "`5...Bh5` line looks quite natural ... [however, AZ's move] `5... Bf5` with the concept in mind **is very interesting** as after `8.d4` [the continuation for Black of] `Nbd7-Bd6` is more natural but `Bb4` **is something new**. I was curious about the idea after `11.Bb2 Nd7 12. Bd3` where `h5!?` was probably the point."

We explore the question posed by the grandmaster – what happens after `11.Bb2`? The ideas is

`5...Bf5 6.Nc3 h6 7.Bb2 Nf6 8.d4 Bb4 9.a3 Bxc3+ 10.Bxc3 Ne4 11.Bb2 Nbd7 12.Bd3 h5 13.0-0 g5!`

This resulting chess position is shown on the left of Figure S10. If White tries to castle kingside with `13.Qd2` instead of `13.0-0`, Black can pursue a king-side pawn advance:

`13.Qc2 Rh6 14.0-0-0 b5!?.`

The resulting chess position is shown in the right chess position in Figure S10. Both continuations are unorthodox; conventional human-designed chess principles emphasise completing piece development, securing the king's safety and maintaining the bishop pair over trading it for a knight, as outlined in (2). However, AZ deviates from these principles favouring a continuation that prioritises a strong control of the centre, space, and piece activity.

Fig. S9. Concept Puzzle 1: Black is to move.

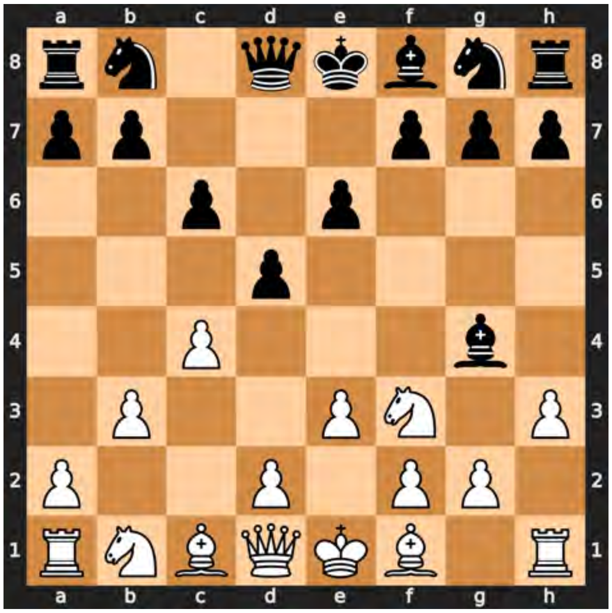

AZ's calculations: `5...Bf5 (5...Bh5 6.Bb2 Nd7 7.cxd5 cxd5 8.Nc3 a6 9.Rc1 Ngf6 10.g4 Bg6 11.Nh4 Be4 12.Nxe4 Nxe4 13.Nf3) 6.Nc3 h6 7.Bb2 Nf6 8.d4 Bb4 9.a3 Bxc3+ 10.Bxc3 Ne4 11.Rc1 Nxc3 12.Rxc3 Qe7 13.Qc1 O-O 14.Be2 Nd7 Approximately equal`

Fig. S10. Digging Deeper into the Concept in Figure S9. In both positions, White is to play.

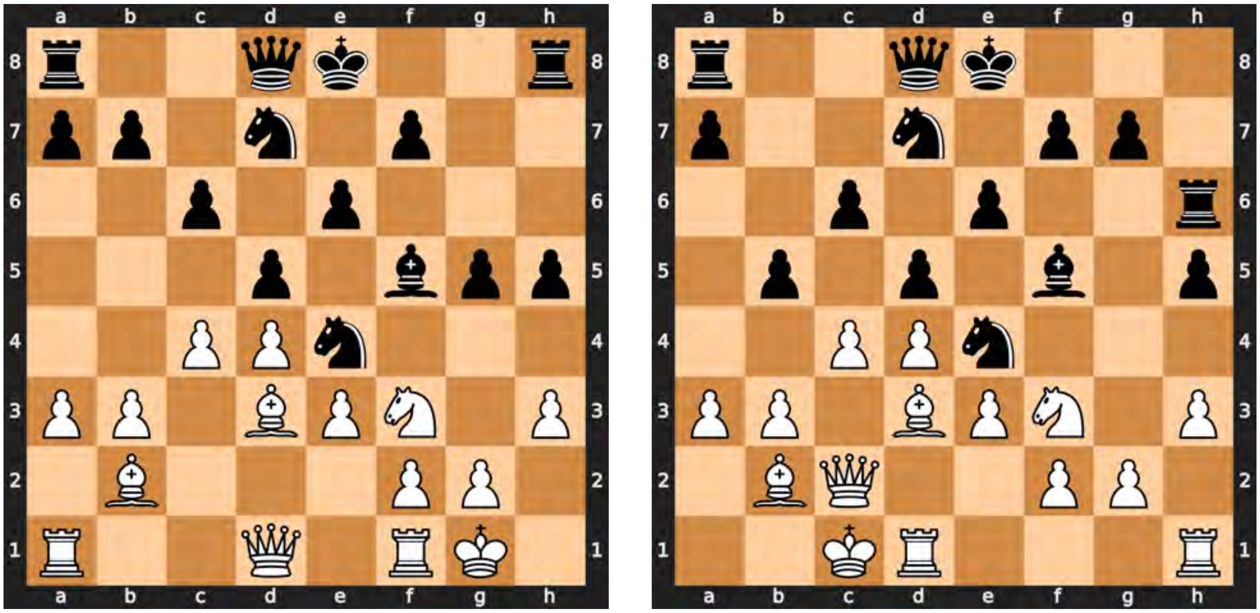

Another puzzle from the same concept is shown in Figure S11, given in Phase 3. Here, the grandmaster found the best continuation according to AZ: `10.Ndf4` threatening `d5`. The ideas are

```
10...a6 11.Qa4
10...Bd7 11.Bc4 gaining control over the square e6
10...d5? 11.Qb3 and the pawn on d5 is lost
10...Bf6 Black's best option 11.Bxf6 exf6 (12.d5? a6 13.Qa4 then Black has the intermediate move 13...Re4!) 12.Ng1 a6 13.Ba4 Bd7 14.Nge2
```

The knight manoeuvres `10.Ndf4` and `12.Ng1` are against the common rules which advocate for finishing piece development and bringing the king to safety, above further improving a developed piece (2–4). As in the previous puzzle, AZ prioritises

Fig. S11. Concept Puzzle 2: White is to play.

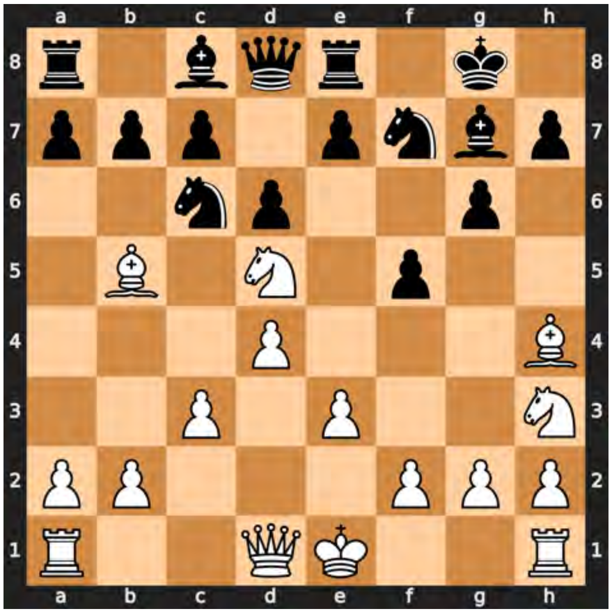

controlling the centre and piece activity.

The grandmaster missed the idea 12.Ng1, although did appreciate it remarking that "`Ng1` [is] quite nice actually, [knight] on `h3` is gone, and then we probably go for `h4` at some point."

Informative puzzles. In some puzzles, we observe manoeuvres from AZ. We provide a few examples here.

Fig. S12. Queen Manoeuvre. White is to move.

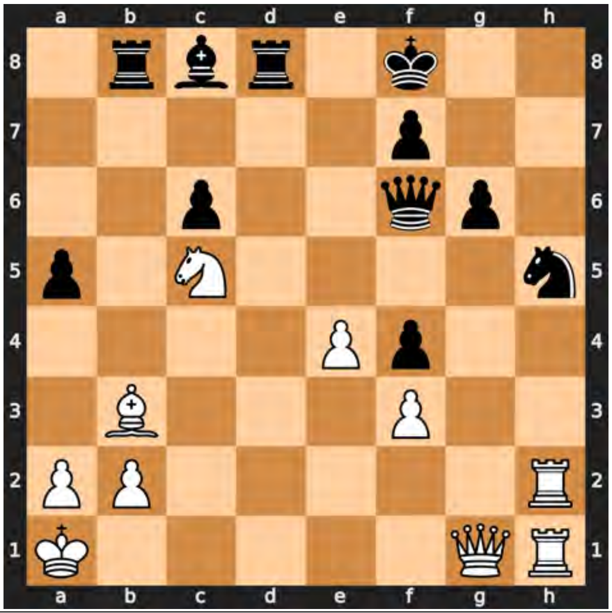

AZ's calculations: `37.Qc1 Kg7 (37...Rb5 38.a4 Rb4 39.Ka2; 37...Qe5 38.Qc4 Be6 39.Nxe6+ fxe6 40.Qxc6) 38.Re1 Qe5 39.Rc2 Rb4 40.Ba4 Qd6 41.a3 Rd4 42.e5 Qd5 43.Bxc6 Qxc6 44.Nb3 White is better`

In the chess position in Figure S12, AZ plays `Qc1` with the idea of manoeuvring it to `c4`. Most human chess players would find this idea unconventional, as White's pieces seem to be active on the kingside. However, there is no way to break through Black's position. AZ's idea is the only way to maintain an advantage. The plan is to re-position the pieces to the queenside, with ideas like `Re1, Ba4 and e5`.

When analysing this position (and only spending a fixed amount of time), the grandmaster misted the idea and opted for `Rxh5`, which was the only way for White to equalise according to the grandmaster. We speculate that the difference between

AZ and humans is because AZ is more flexible in changing its plan. In this position, humans are likely primed to continue playing on the kingside.

Fig. S13. Positional Tactics. White is to move in both positions.

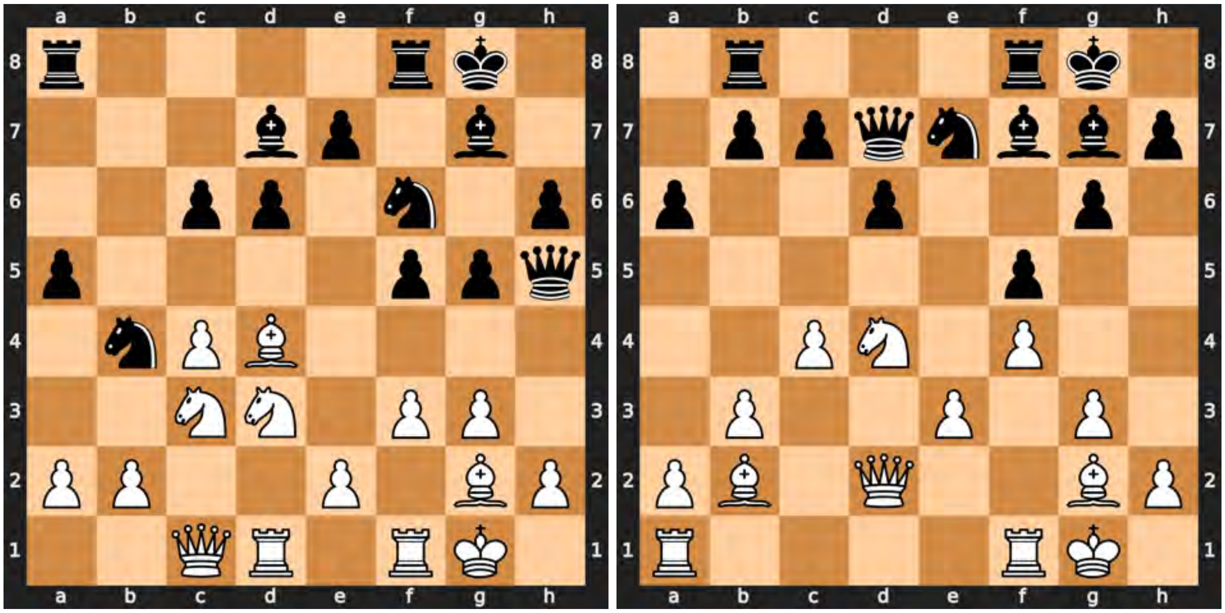

AZ's calculations (left): `21.Nc5 dxc5 (21...Bc8 22.N5a4 Be6 (22...c5 23.Bxf6 Bxf6 24.a3 Ra6 25.f4) (22...f4 23.Nb6 Rb8 24.Nxc8 Rbxc8 25.a3 Na6 26.e3) 23.a3 Na6 24.c5 dxc5 25.Nxc5 Nxc5 26.Bxc5) 22.Bxf6 Bxf6 23.Rxd7 Bd4+ 24.Kh1 f4 (24...Qe8 25.Rb7 Rf7 26.f4 Qc8 27.Rb6 Qd8 28.Na4) 25.Ne4 White is slightly better`

AZ's calculations (right): `18.Rad1 Rbe8 (18...b5 19.c5 Rbe8 20.Ba3 Nd5 21.Rfe1 b4 22.Bxb4 Nxb4 23.Qxb4) 19.Nf3 Bxb2 20.Qxb2 Nc6 21.Rfe1 h6 22.Qc3 Re7 23.b4 Rfe8 24.a4 White is better`

The puzzles in Figure S13 correspond to the same concept. In both positions, AZ uses tactics to obtain a positional advantage by maintaining a space advantage. In the left puzzle, the best move is `21.Nc5`, which stops Black from advancing their *c*5 pawn to control the center. The tactic behind the idea is `21.Nc5 dxc5 22.Bxf6 Bxf6 23.Rxd7`.

In the puzzle on the right of Figure S13, Black plays `18.Rad1` which is prophylactic against `18...b5` as White has tactic `19.c5 dxc5 20.Nc6`. Both of these continuations were found by the grandmaster, who appreciated the importance of `Nc5`. The grandmaster explained that they took a long time to analyse this position as they found it complicated. While recognising Black's threat of c5 (followed by `Bc6` or `Nc6`), they first explored several other options, including moves such as `a3`, `c5`, `Bf2` or `Na4`. However, after exploring other moves, they found `Nc5`. The grandmaster commented that the idea was very strong and 'by far the best move'.

In this puzzle, the grandmaster opted for the tactical move `Bxh7` but also considered quieter moves such as `Re3`, `Be4` or `Be1`. When analysing this position, they commented

> "This is tricky, if White decides to protect the pawn, it's clearly better due to the weakness in Black's structure, but somehow getting addicted to more forced attacking lines. `Bxh7` is hard to figure out, maybe `Kxh7` [followed by] `Qh5-Qf7` then `Rd3`, `Bxg2` ... [I] didn't calculate until clear much better position, but thought even with some play, h-file the long problem, maybe certain chances [to win]."

However, this sequence ends in a draw, and AZ instead opts for `f3`, maintaining the advantage for White. When reading AZ's analysis, the grandmaster commented:

> "Wow, it's a completely positional play. Well, my decision to [sacrifice] is too emotional. To be honest, this choice `f3` ... [makes] sense as Black does not have any breakthrough idea, so if White successful [in] controlling both `c5` and `e5` square then its clearly much better. [My conclusion is] technically strong but again within [the] human perspective."

Here, we see a difference in style between humans and AZ. AZ opts for a slower, longer-dominance play in chess positions where grandmasters tend to consider more forcing sequences.

Human vs AI Play: AZ opts for less forced lines than humans.

Fig. S14. White is to move.

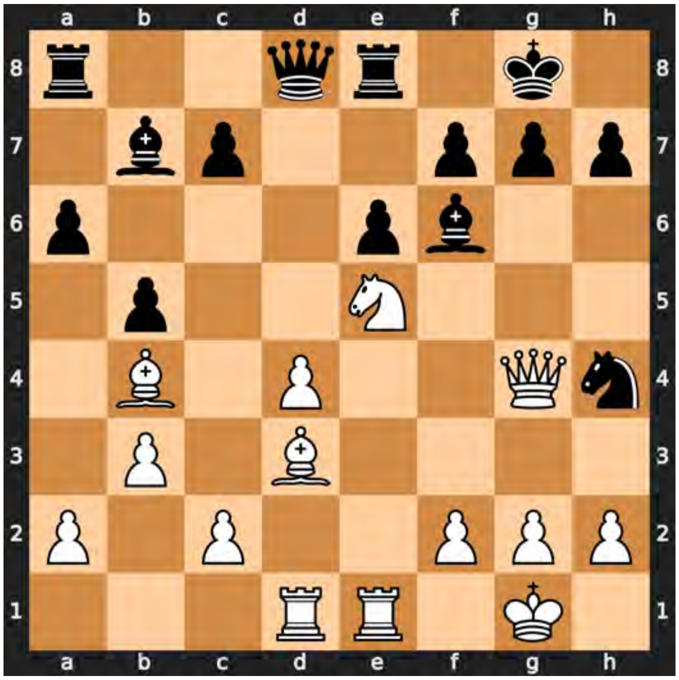

**AZ's calculations:** `18.f3 (18.Bf1 Ng6 (18...Nf5 19.c4 g6 20.cxb5 axb5 21.Bxb5 h5 22.Qh3 Rxa2 23.Bxe8 Qxe8) 19.Bd3 Nh4 (19...Bh4 20.Bxg6 hxg6 21.Rd3) 20.f3) 18...Ng6 19.Bc5 Bh4 20.Bxg6 hxg6 21.g3 White is better`

Fig. S15. AZ takes on more risk than humans due to computational capacity. White is to move.

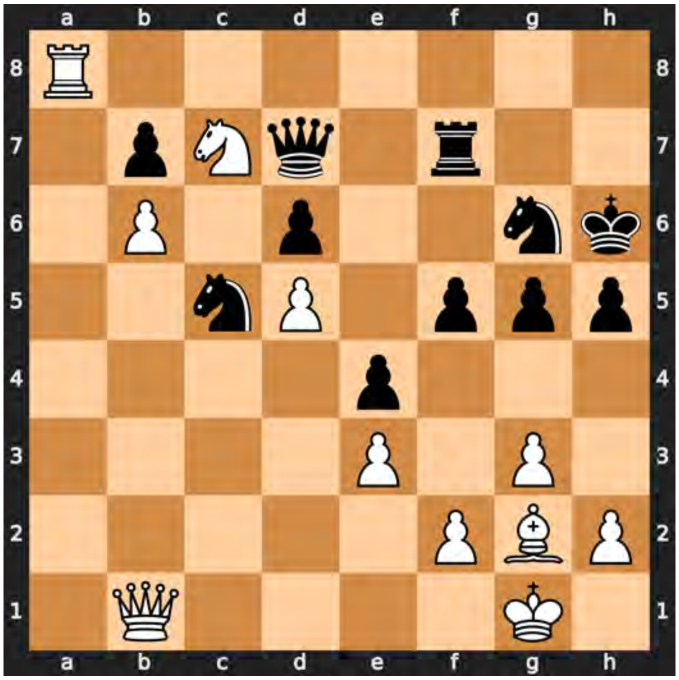

**AZ's calculations:** `31.Qa1 f4 (31...Qe7 32.Re8 Qf6 33.Qxf6 Rxf6 34.Rd8 Ne5 35.Ne8) (31...h4 32.Re8 f4 33.exf4 gxf4 34.Bxe4 Nxe4 35.Rxe4 Qf5 36.Qe1 Ne5 37.Qe2 hxg3 38.hxg3 fxg3 39.fxg3) 32.exf4 gxf4 33.Ne6 Nxe6 34.Bh3 Qb5 35.dxe6 Rg7 36.Ra5 White is slightly better`

Difficult or non-instructive puzzles. There may exist concepts that are intrinsically hard to understand and learn for human chess players due to differences in ways of abstract thinking, overall capabilities, and their computational budgets. The example in

Figure S15 highlights that humans and AI have different computational capacity, allowing AZ to make moves that appear risky to humans.

In the puzzle in Figure S15, AZ plays `Qa1` to activate the queen. This move requires calculating carefully to ensure that Black has no counterplay due to an attack on the kingside. As such, humans may perceive this move as risky, and it was not chosen by the grandmaster. When seeing AZ's calculations, they remarked

> "I would be really worrie[d] to keep the queens on the board because of the threat with `f4` but AZ has a tactical solution. `33.Ne6 Nxe6 [34.]Bh3` is a very nice idea which is quite hard to spot. Black should probably stay still and try to hold with `31...h4 [32].Re8 Re7`."

Here, we see that humans are more risk-averse than AZ. This is logical, given that AZ has a much larger computational capacity and can calculate more/deeper than humans can to more accurately assess the chess position (and thereby take on less risk).

Fig. S16. AZ simplifies the chess position for the draw whereas humans would continue to try to win. Black is to move.

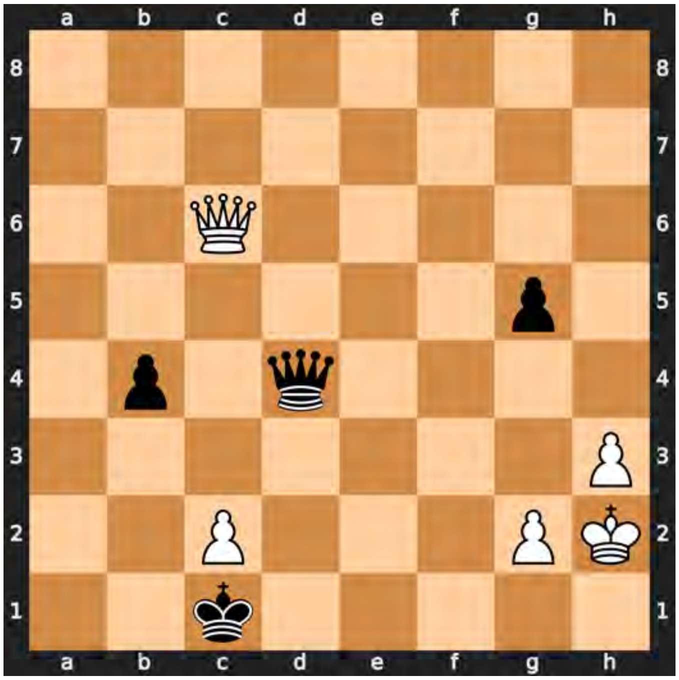

AZ's calculations: `58...g4 59.hxg4 (59.Qc7 gxh3 60.Kxh3 Qc3+) 59...Qxg4 60.Qc5 Qh4+ 61.Kg1 Qe1+ 62.Kh2 Qc3 (62...Qh4+) Draw`

Differences in motivation in human and AI play. The next example shows that AZ and humans play chess with different motivations. AZ forces the draw with `g4`. Upon seeing AZ's calculations, the grandmaster commented:

> "... this is a very clear and important theme to understand. So, g4, the move it proposes, in a practical sense it's a very big move, because you see, in such situations, the engine already knows the final result. For engine it doesn't matter which move it plays because it calculated it's a draw, but g4 is basically forcing it. After `g4` Black has no winning chances, but otherwise I have a feeling that after Black plays let's say `Qd2`, it's not ... easy practically for White to make the draw. For an engine it's ok, but practically no one would play it because `g4` is basically offering a draw - and with other moves Black is running zero risk, yet has practical chances to win the game if White makes a mistake. An engine doesn't understand the concept of practical play - while this is a draw, it's not an easy draw for White. `g4` is one of many moves leading to a draw, but in a practical sense the worst one as it gives Black zero chances to win. So that is my understanding, that it's not the objective best move. Practically definitely a wrong move."

This underscores a fundamental difference between AZ's playing style and human's playing style. AZ was trained to obtain the *expected outcome* without an explicit term in the loss function, encouraging it to win. The incentive to find the best move comes from the exploration and move selection criteria in MCTS. Further, AZ assumes an equally strong opponent. For AZ, there is no difference between different equalising moves, even if one move requires a much more precise sequence of moves to equalise. In contrast, humans assume that their opponent may make suboptimal moves. As human chess players play competitively (i.e., their goal is to maximise the outcome), they will try to leverage these chances. This example highlights how the difference in objectives and assumptions may lead to different behaviour of AZ and humans when playing chess.

Shortcomings of method for generating prototypes. In this puzzle, AZ chooses `Rd1` whereas the grandmaster wanted to play `Re1` or `Ke1`. When seeing AZ's calculations, the grandmaster commented:

> "Drawing position? At first trying to find winning moves for White, but really didn't see any plan to make improvements. In the meantime, considering the possibility for Black to push h pawn to h3, maybe tiny chances, its better to plan `Ke1-Qf1-Qf3` at the beginning, or moving the rook to e1 with the idea `Re7`, forcing ... `Ra1` check then White rook retreat to `e1`, ... [draw by] repetition."

Fig. S17. White is to move

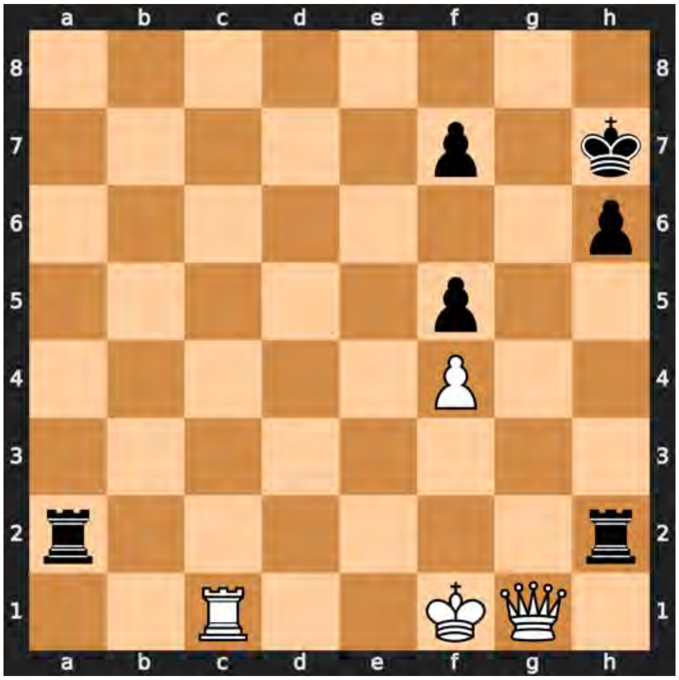

AZ's calculations:

`65.Rd1 Rh4 (65...Rac2 66.Rb1 Rh4 67.Ra1 Rxf4+ 68.Ke1 Rg4 69.Qa7 Kg7) 66.Ke1 (66.Rb1 Rxf4+ 67.Ke1 Re4+ 68.Kd1 Rf4 69.Qh1 Rff2) 66...Rhh2 67.Rc1 Rag2 68.Qf1 h5 69.Rc7 Kg7 Draw`

This puzzle can be seen as a shortcoming of our method for finding prototypes. We only filter positions based on the criteria described in §9; however, this position does not fall under one of our categories. This puzzle is not informative for humans as there are many other viable options. As such, it is more difficult to understand the concept from the sequence of moves.

C. Differences between humans and AZ. In this section, we share a few observed differences between the grandmasters and AZ and speculate where they come from. While we do not have definitive answers, the discussion may lead to further research.

The qualitative examples suggest that AZ has different priors over the relevance of concepts in a chess position than humans. Human chess players formulate and adopt heuristic chess principles to inform their analysis, predisposing them to biases that influence which concepts they deem relevant for specific chess positions. An example is the three 'golden rules' of the opening: control the centre, develop your pieces, and bring your king to safety (2–4). Consequentially, in opening, humans may focus on moves that align with these guidelines. Instead, AZ is self-taught and does not seem to have the same priors over chess concepts as humans. We believe this lack of prior allows AZ to be more flexible – it can apply concepts to various different chess positions and change plans quickly. In essence, AZ formulates its own priors over the relevance of chess concepts for a given chess position. Examples of this behavior are that AZ plays over the entire board, as opposed to focusing on a specific side (see, e.g., Figures S9, S10, S5, and S12); places less importance on the material value of pieces, and prioritises space and piece activity (see, e.g., Figures S2 or S7). This may result in the super-human application of concepts, and new concepts.

One may ask where do the differences between AZ's and humans' play come from? We conjecture that they may arise from differences in objectives and capabilities. AZ learnt to play chess against itself. As such, AZ assumes optimal play and information symmetry.[†] On the other hand, humans play chess against other humans and, therefore, may assume information asymmetry and imperfect play. This leads to a difference in behaviour: while AZ focuses on finding the best move, human chess players often make *practical* choices. Humans' choices do not always increase the expected outcome against an optimal opponent (their choices may even slightly decrease the expected outcome) but may increase their odds against another human.

[†] With information symmetry, we mean that Black and White have the same general knowledge and perform the same calculations in a given chess position.

For example, in drawn chess positions, humans may try to complicate the chess position or opt for continuations where the best moves are less clear-cut in hopes that their opponent makes a mistake (see Figure S16). However, AZ will try to find the optimal plan, disregarding aspects such as complexity. Therefore, AZ's play may be fundamentally different and better reflect conceptually relevant plans in a chess position.

Another difference between humans and AZ's play is the role of time. Humans have limited energy[‡] and time allocation for a game. In chess positions where humans are better, they often simplify the chess position to try to secure the win as quickly as possible and minimise risk (see, e.g., the grandmasters' chosen moves in Figures S15 and S14). However, AZ does not care about how quickly the game finishes. The training loss function does not have a penalty term to encourage winning as quickly as possible. As a result, it has a different treatment of time. This results in sometimes choosing slow strategic wins (as can be seen in the chess positions in Figure S14). While the lack of time constraint may lead to super-human concepts, it also may result in complex concepts that are difficult for humans to learn.

Naturally, AZ and humans have different computational capacities. As a result, AZ can opt for more computationally expensive moves and, therefore, defend complicated chess positions where humans might be more hesitant. In terms of playing style, AZ will often opt for what it believes is the *optimal* move, while humans have a more limited computing budget and may opt for a safer move. In chess, *safer* is used to describe continuation where there is less probability of making an incorrect move. Sometimes, humans may even play a slightly suboptimal move to minimise the risk. We see this phenomenon in Figures S15 and S16. While computational capacity cannot be transferred, it may still lead to super-human concepts, as AZ can find new ideas that can still be taught to humans.

2. Background: Chess in AZ and Humans

A. AZ policy value network. For a complete description of AZ, see (6) or (7). Here, we provide a brief description. AZ has two main components: a policy value network and MCTS. Below, we describe the policy-value network.

For a given input (consisting of a chess position and metadata), the network outputs a policy and value estimate. As described in (7), the main 'body' of the network has a ResNet backbone. The body consists of 19 residual blocks. Each block contains two convolutional layers, followed by a skip connection. Let $\mathbf{z}_l$ denote the post-activation latent representation corresponding to block l:

$$\mathbf{z}_l = \mathrm{ReLU}(\mathbf{z}_{l-1} + g_l(\mathbf{z}_{l-1})), \quad [1]$$

where $g_l(\cdot)$ is the composition of two convolution layers.

In the main body of the text, we refer to layers using integers. For the main body of the network, these integers denote the ResNet block. In the value head, we have three layers, which we will refer to as layers 20, 21 and 22 (from the body to value output). In the policy head, we have two layers, which we refer to as layers 23 and 24 (from body to policy output).

B. Background: how humans and AZ play chess. In this section, we provide further intuition on how humans play chess, and how this relates to AZ's system. This section provides context as to why concepts should explain the policy value network and MCTS to provide a holistic view of chess.

Figure S19 shows a simplified summary of how human chess players analyse a chess position. They generally ask the following questions:

1. What are the critical aspects of this chess position? E.g., on which side of the chess board do I have more space? How do I want to develop my pieces? What are my opponent's weaknesses?

2. Based on step 1, a chess player will find a *couple candidate moves* – actions they could play in the chess position.

3. For each move, they may calculate a likely continuation – i.e., what is the likely sequence of moves to follow?

This process loops until the player has considered all candidate moves, calculated the relevant move sequences, and determined the optimal trajectory.[§] To play well, chess players must understand the important features of a chess position and calculate move sequences to understand the correct evaluation of the chess position. However, there are several different types of chess positions (e.g., endgames or attacking chess positions) where principles alone are insufficient to determine the optimal continuation, and calculation is necessary.

AZ uses a similar approach. In a given chess position, AZ extracts features using the layers in the policy-value network and outputs a policy and value estimate. The policy weighs the different possible actions, and the moves with the most probability mass can be interpreted as the *candidate moves*. Next, the policy is passed on as an input to MCTS, a search algorithm that calculates the optimal move. By drawing parallels between AZ's system and how humans play chess, we highlight the importance of each component of AZ. This motivates our method's design, which incorporates all components of AZ.

[‡] Classical time control (see match time controls in 5) chess games take hours.

[§] This is a simplified model - in practice, other factors such as time are important.

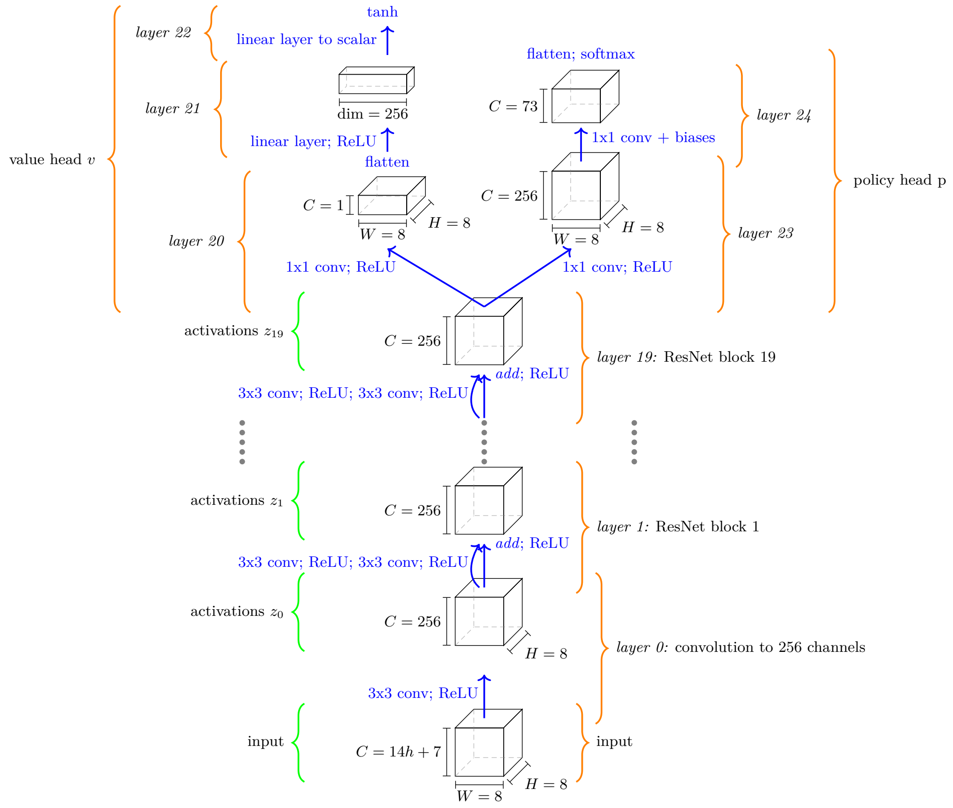

Fig. S18. Policy Value Network in AlphaZero. Adapted from ref. (7), which is licensed under CC BY 4.0.

3. Concept constraints for static concepts

Static concepts are defined as concepts that only involve a single state. We use *supervised data* (labels indicate whether a state contains a concept c) to learn static concept vectors. These concepts encode human knowledge, and therefore, we can use them to validate our approach. One example of a static concept is the concept of 'space', which we can infer from a single state. For now, assume we have binary concepts[¶] and denote the presence of concept c (concept score) in chess position $\mathbf{x}$ by $c(\mathbf{x}) = 1$, and $c(\mathbf{x}) = 0$ otherwise. For each concept c, we can split a general set of chess positions $\mathbb{X}$ into positive examples $\mathbb{X}^+$, where the concept is present, and $\mathbb{X}^-$, where it is absent

$$\mathbb{X}^+ = \{\mathbf{x} \in \mathbb{X} : c(\mathbf{x}) = 1\}$$
$$\mathbb{X}^- = \{\mathbf{x} \in \mathbb{X} : c(\mathbf{x}) = 0\}.$$

These positive and negative examples allow us to generate corresponding positive and negative examples of latent representations (intermediate post-activation representations in the network). The function $f_l(\mathbf{x})$ generates an activation for layer l given an input $\mathbf{x}$:

$$\mathbb{Z}_l^+ = \{f_l(\mathbf{x}) : \mathbf{x} \in \mathbb{X}^+\}$$
$$\mathbb{Z}_l^- = \{f_l(\mathbf{x}) : \mathbf{x} \in \mathbb{X}^-\},$$

where $z_l = f_l(\mathbf{x})$ denotes the latent representation obtained at layer l by passing input $\mathbf{x}$ through the network. See §A for further details on how z_l is extracted.

The convex optimisation goal is to learn a sparse vector $\mathbf{v}_{c,l}$ that represents a concept c. We hypothesise that the inner product $\mathbf{v}_{c,l}^\top \mathbf{z}^l$ is higher[‖] for activations from $\mathbb{Z}_l^+$ (the set where the concept is present) than for activations from $\mathbb{Z}_l^-$ (the set where the concept is absent). Thus, the formulation becomes

$$\begin{aligned} \min \quad & \|\mathbf{v}_{c,l}\|_1 \\ \text{such that} \quad & \mathbf{v}_{c,l}^\top \mathbf{z}_l^+ \geq \mathbf{v}_{c,l}^\top \mathbf{z}_l^- \quad \text{for all } \mathbf{z}_l^+ \in \mathbb{Z}_l^+ ,\ \mathbf{z}_l^- \in \mathbb{Z}_l^- \end{aligned} \qquad [2]$$

We can evaluate how well a concept is represented by $\mathbf{v}_{c,l}$ in the supervised setting by splitting $\mathbb{X}$ into two sets: $\mathbb{X}_{\text{train}}$ and $\mathbb{X}_{\text{test}}$ and then $\mathbf{v}_{c,l}$ only using $\mathbb{X}_{\text{train}}$. We then measure the fraction of elements in $\mathbb{X}_{\text{test}}$ on which the concept constraints hold.

[¶] We show how to handle non-binary concepts in §C.

[‖] A larger inner product corresponds to a higher cosine similarly.

Fig. S19. Simplified Summary of How Humans Play Chess

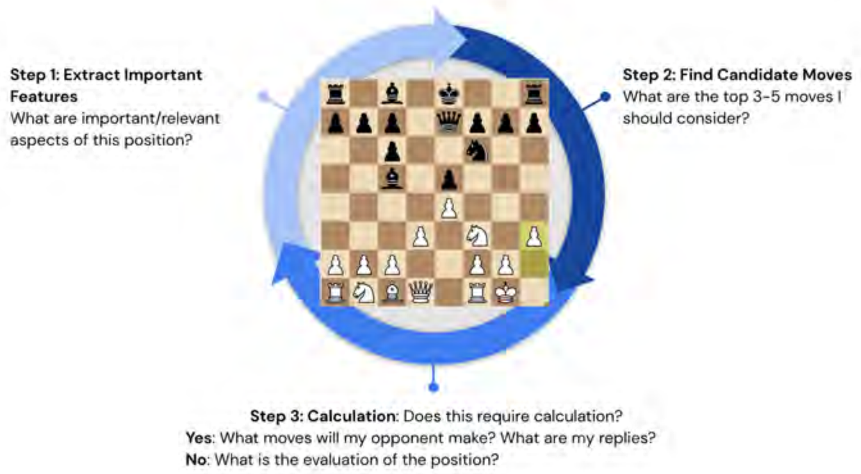

Fig. S20. Simplified Summary of How AZ Plays Chess

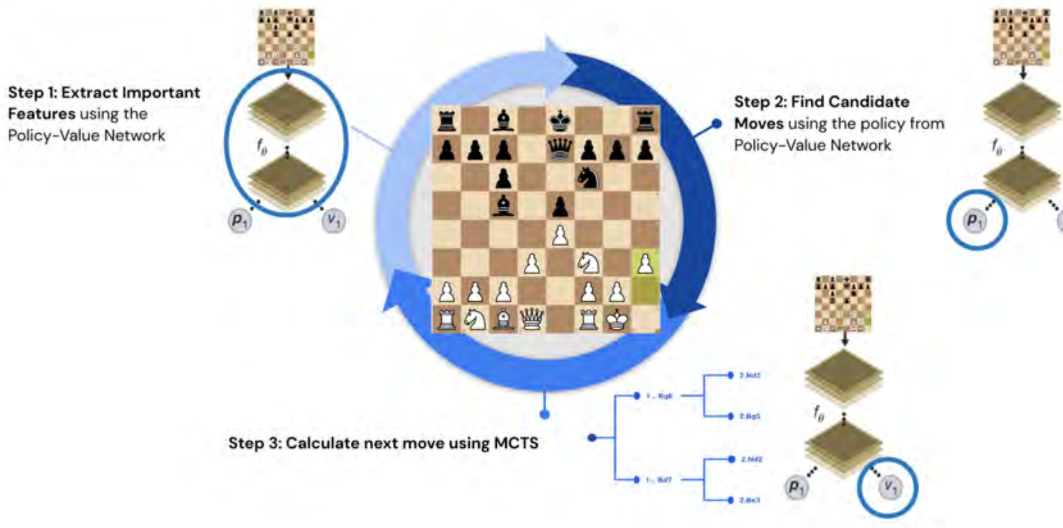

If $\mathbf{v}_{c,l}$ represents the concept c well, we expect the concept constraint to hold on previously unseen activations derived from $\mathbb{X}_{\text{test}}$.

Prototypes For **static concepts**, we find prototype positions for c by computing a concept score $c(\mathbf{x}) = \mathbf{v}_{c,l}^{\top} f_l(\mathbf{x})$ for every $\mathbf{x} \in \mathbb{X}$, where $f_l(\mathbf{x})$ indicates layer l of AZ neural network f. We then selecting the top 2.5% of $\mathbb{X}$ according to the concept score $c(\mathbf{x})$. We use 2.5%, as we found the concept score $c(\mathbf{x})$ to be comparable to $\mathbf{v}_{c,l}^{\top}\mathbf{z}_l^{+}$, where $z_l^{+} \in \mathbb{Z}_l^{+}$ is the training set used to find $\mathbf{v}_{c,l}$. This procedure gives us a prototype set $\mathbb{X}^{\text{proto}} = \{\mathbf{x} \in \mathbb{X} : c(\mathbf{x}) \text{ in 2.5th percentile of } c(\mathbf{x})\}$.

4. Method evaluation

This section includes algorithmic evaluations of our proposed concept discovery method. Table S1 summarises the datasets used; further details on each dataset can be found in §B, and implementation details for each dataset can be found in §C.

A. Evaluation of the proposed convex optimisation framework for finding concept vectors. Using the datasets mentioned above, we find concept vectors using the approaches described in §3 and §2. While we use layer 19 for all other sections due to its potential novelty according to spectral analysis in §2, we conduct our evaluation on a few additional layers here: the first latent representation in the policy head (layer 23); and the latent representations in the value head (layer 20 and 21) (See §A). These layers are selected due to their proximity to the network outputs – the policy and value estimate.

We first validate our approach by showing the convex optimisation formulation can be used to find the vector representations of a concept using labelled data (§B) and show that this can be done efficiently with a small number of labels (§C). Next, we

Table S1. Datasets Summaries: from concepts more known to humans (top rows) to AZ (bottom rows). S denotes strategic and T denotes tactical.

Name	Concept Type S vs. T	Concept Type Game Phase	Type of Knowledge	Complexity
Piece	N/A	All	Human	Low
Stockfish	Both	All	Human	Varies
Strategic Test Suite (STS)	S	Middle/End	Human	Medium
Opening	S	Begin	Human/AZ	Varies
AlphaZero (games)	Both	All	AZ	High

further validate our approach by showing that amplifying the concept vector in the latent representation makes AZ's moves to be more similar to the concept (§C.1). This section focuses on validating the convex optimisation framework.

B. Do the concept constraints hold for a test dataset? . To evaluate our framework and thus the quality of the vector representations of the supervised concept, we measure the percentage of times the concept constraints hold on the test set (80/20 train/test split), as shown in Table S2. We find that most datasets led to a high accuracy, which is an indication of quality concept vectors. A concept-level breakdown of the accuracy for each dataset can be found in §A.

Table S2. Evaluation: % the concept constraints hold on test data. The standard error is shown in parentheses.

Concept	Layer 19	Layer 20	Layer 21	Layer 23
Pieces	0.99 (0.00)	0.98 (0.00)	0.95 (0.00)	0.99 (0.00)
Stockfish	0.76 (0.03)	0.75 (0.03)	0.73 (0.02)	0.77 (0.02)
STS	0.92 (0.09)	0.92 (0.09)	0.92 (0.06)	0.90 (0.06)
Opening (general)	1.00 (0.00)	1.00 (0.00)	1.00 (0.00)	1.00 (0.00)
Openings (per line)	0.99 (0.13)	0.99 (0.14)	0.99 (0.14)	0.99 (0.13)

C. How many data points do we need to learn a concept?. We can use labelled examples to evaluate the concept vector quality (as in the previous section), but they can be hard to come by in practice. This section shows that our formulation can find concept vectors efficiently using a few examples (for the concept constraints). We measure the test set accuracy (as in §B) while varying sizes of the training set for two datasets: pieces and the strategic test suite (STS).

Fig. S21. Sample efficiency of convex optimisation framework, averaged across 10 seeds, for the bottleneck layer (19), value head (20) and policy head (23).

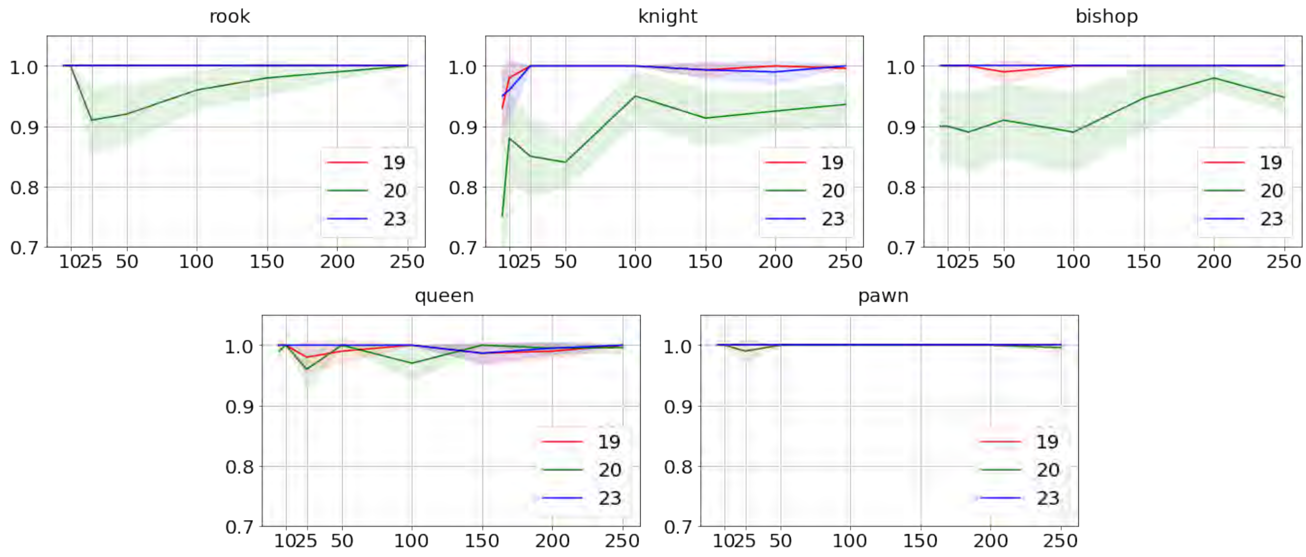

As shown in Figure S21, we find that the method reaches close to full-set accuracy with only a few samples – often as little as 10 data points on the pieces dataset. Interestingly, we observe relatively lower performance in the value head (layer 20) than the policy head (layer 23). One potential explanation is that the concept of a specific piece no longer has to exist when estimating the value, which is a scalar – a highly compressed representation of the state of the game. We speculate that it is

possible that simple concepts are combined with other concepts. For example, the network may encode the presence of light pieces, i.e., bishops and knights, rather than the presence of bishops. This may explain the relatively low, but not significantly lower performance in layer 20.

Figure S22 shows the result for sample efficiency experiment (§C) on the STS dataset. As with the piece dataset, we find that the convex optimisation method reaches close to full-set accuracy with as little as 10 or 25 data points. However, contrary to the piece dataset, we find that the performance is relatively similar across all layers, and slightly lower for the policy head (layer 23). One reason may be that these concepts are more important for the value than the policy.

Fig. S22. Sample efficiency of convex optimisation framework across 10 seeds for the bottleneck layer (19), value head (20) and policy head (21, 23).

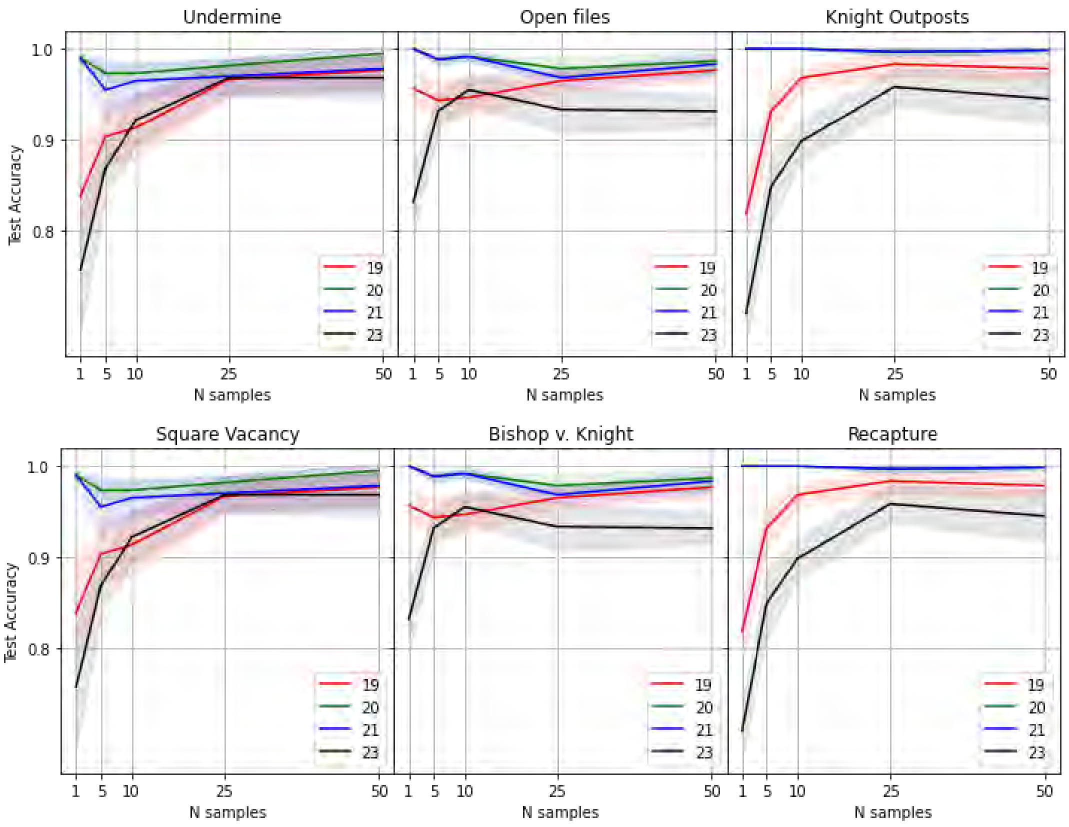

C.1. Does amplifying concept vectors increase concept-related behavior?. We want to (1) determine whether the concept vector captures the intended concept and (2) understand whether the concept influences AZ's output (policy). (2) is important as a concept may exist in a latent representation but not be used by the network for its predictions. To investigate these properties, we use concept amplification. Let $\mathbf{z}_l$ denote the latent representation in layer l of a chess position $\mathbf{x}$. To amplify the presence of a concept, we nudge the latent representation in the direction of the concept vector $\mathbf{v}_{c,l}$

$$\tilde{\mathbf{z}}_l = (1 - \alpha)\mathbf{z}_l + \alpha\beta \frac{||\mathbf{z}_l||}{||\mathbf{v}_{c,l}||}\mathbf{v}_{c,l}, \quad [3]$$

where $\alpha \in [0, 1]$ and β are hyper-parameters for the size of the perturbation; and $|| \cdot ||$ is the ℓ_2 norm. We use cross-validation to determine β, and find that $\beta = 0.01$ is the best overall (see §D). We report our results for various α in Figure S23 using the STS dataset. The STS dataset includes different types of puzzles grouped according to a strategic theme (such as 'square vacancy'). For each concept, there are 100 chess positions ($\mathbb{X}$) and a solution set $\mathbb{S}_i$ for each chess position $\mathbf{x}_i \in \mathbb{X}$. The solution moves require applying the concept given a chess position. As a baseline, we first evaluate AZ's performance by recording the percentage of times the move selected by AZ under the policy is in the solution set:

$$A = \sum_i \mathbb{1}[\text{argmax } \pi_l(\mathbf{z}_{i,l}) \in \mathbb{S}_i], \quad [4]$$

where $\pi_l(\mathbf{z}_{i,l})$ is AZ's policy on the latent representation $z_{i,l}$, and $\mathbb{1}[\cdot]$ is an indicator function that is equal to 1 if the move selected by AZ is in the solution set $\mathbb{S}_i$. We compare the performance difference between with and without concept amplification and report the normalised values $(\tilde{A} - A)/A$ in Figure S23.

As this experiment focuses on the impact of the concept on the predicted move (only the policy output, not the value), we analyse the performance of concepts found in layers 18 and 19 (layers before policy and value head split, see §A), and layer 23 (policy head).

Fig. S23. Performance improvement in solving puzzles using STS dataset across different α values (x-axis). Layers 18 (left) and 19 (centre) are before value/policy head split, and the policy head (layer 23) (right). Each line indicates a set of concepts with different quality (measured by test accuracy as done in §B). Higher quality (high threshold, orange line) achieves the highest improvement.

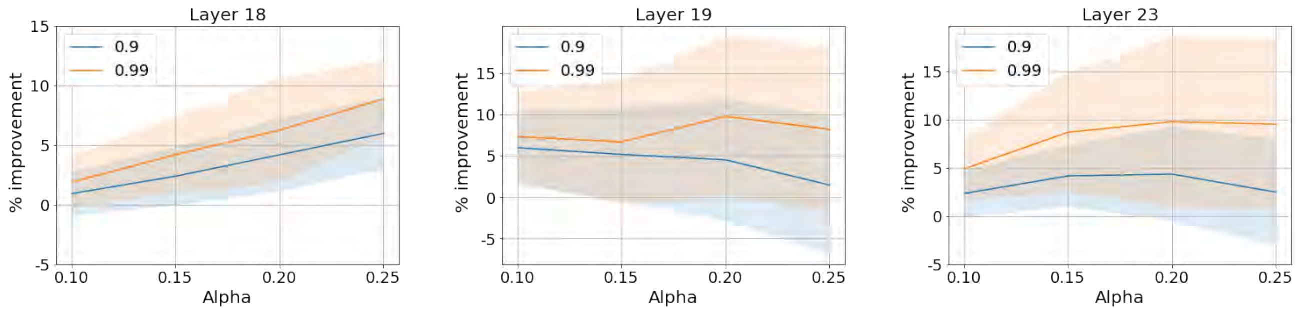

Each line in Figure S23 represents the results for a different concept quality, where concept quality is measured by test accuracy as in §B. We observe that amplifying the concept can improve AZ's performance on the puzzles of the concept. Naturally, the quality of concepts influences this; concepts with higher test accuracy lead to a larger performance improvement, suggesting a higher test score is a good proxy for how well the concept vector captures the semantic meaning of the concept.

5. Extra results

Table S3. Constraint Satisfaction Results for Piece Dataset

Concept	layer 19	layer 20	layer 21	layer 23
rook	1.0	1.0	1.0	1.0
knight	1.0	1.0	1.0	1.0
bishop	1.0	1.0	1.0	1.0
queen	1.0	1.0	1.0	1.0
pawn	1.0	1.0	1.0	1.0

A. Concept constraint satisfaction. In the table below, the concepts are extracted from Stockfish 8's public API. Further details can be found in Appendix A of (7).

Table S4. Concept constraint Satisfaction Results for Stockfish Dataset. w/b/t denotes White/Black/total difference; mg/eg/ph denotes middle game/ endgame/phased value, where the phased value is the weighted sum between the middle and endgame values, depending on the phase of the game.

Concept		layer 19	layer 20	layer 21	layer 23
bishop	[b,eg]	1.0	1.0	1.0	1.0
bishop	[b,mg]	1.0	0.9	0.9	1.0
bishop	[b,ph]	0.9	0.9	0.9	1.0
bishop	[t,eg]	0.7	0.7	0.4	0.5
bishop	[t,mg]	0.6	0.7	0.8	0.6
bishop	[t,ph]	0.7	0.6	0.7	0.4
bishop	[w,eg]	1.0	1.0	0.9	1.0
bishop	[w,mg]	1.0	1.0	1.0	1.0
bishop	[w,ph]	1.0	1.0	0.5	1.0
imbalance	[t,eg]	0.6	0.2	0.6	0.4
imbalance	[t,mg]	0.6	0.2	0.6	0.4
imbalance	[t,ph]	0.5	0.4	0.5	0.8
king safety	[b,eg]	0.9	0.9	0.9	0.8
king safety	[b,mg]	1.0	1.0	1.0	1.0
king safety	[b,ph]	1.0	1.0	1.0	1.0
king safety	[t,eg]	0.7	0.9	0.6	0.7
king safety	[t,mg]	0.8	0.8	0.7	0.7
king safety	[t,ph]	0.6	0.6	0.7	0.6
king safety	[w,eg]	0.6	0.7	0.6	0.6
king safety	[w,mg]	1.0	1.0	1.0	1.0
king safety	[w,ph]	0.0	1.0	1.0	1.0
knights	[b,eg]	0.0	0.0	0.9	0.0
knights	[b,mg]	0.9	0.9	0.5	0.9
knights	[b,ph]	0.0	0.0	1.0	0.8
knights	[t,eg]	0.6	0.6	0.7	0.7
knights	[t,mg]	0.9	0.5	0.9	0.9
knights	[t,ph]	0.8	0.5	0.4	0.8
knights	[w,eg]	0.9	0.8	0.8	0.9
knights	[w,mg]	1.0	0.8	0.6	0.8
knights	[w,ph]	0.8	0.6	0.3	0.8
material	[t,eg]	0.8	0.7	0.3	0.7
material	[t,mg]	0.6	0.8	0.5	0.6
material	[t,ph]	0.6	0.6	0.6	0.7
mobility	[b,eg]	1.0	1.0	1.0	1.0
mobility	[b,mg]	1.0	1.0	1.0	1.0
mobility	[b,ph]	1.0	1.0	1.0	1.0
mobility	[t,eg]	0.8	0.9	0.8	0.7
mobility	[t,mg]	0.7	0.4	0.6	0.6
mobility	[t,ph]	0.8	0.7	0.7	0.8
mobility	[w,eg]	1.0	1.0	1.0	1.0
mobility	[w,mg]	1.0	1.0	1.0	1.0
mobility	[w,ph]	1.0	1.0	1.0	1.0

Table S5. Concept constraint Satisfaction Results for Stockfish Dataset. w/b/t denotes White/Black/total difference; mg/eg/ph denotes middle game/ endgame/phased value, where the phased value is the weighted sum between the middle and endgame values, depending on the phase of the game.

Concept		layer 19	layer 20	layer 21	layer 23
passed pawns	[b,eg]	0.8	0.8	0.9	0.8
passed pawns	[b,mg]	0.8	0.9	0.7	0.7
passed pawns	[b,ph]	0.9	1.0	0.9	0.9
passed pawns	[t,eg]	0.8	0.9	0.7	0.7
passed pawns	[t,mg]	0.6	0.7	0.3	0.8
passed pawns	[t,ph]	0.6	0.5	0.6	0.7
passed pawns	[w,eg]	1.0	1.0	1.0	1.0
passed pawns	[w,mg]	0.6	0.8	0.6	0.7
passed pawns	[w,ph]	1.0	1.0	0.9	0.9
pawns	[t,eg]	0.6	0.5	0.4	0.5
pawns	[t,mg]	0.3	0.7	0.5	0.4
pawns	[t,ph]	0.8	0.7	0.7	0.7
phase		1.0	1.0	1.0	1.0
queens	[b,eg]	0.3	0.3	0.4	0.3
queens	[b,mg]	0.9	0.9	0.8	0.8
queens	[b,ph]	0.9	0.9	0.7	0.9
queens	[t,eg]	0.3	0.3	0.4	0.3
queens	[t,mg]	0.6	0.4	0.2	0.7
queens	[t,ph]	0.6	0.5	0.6	0.6
queens	[w,eg]	0.3	0.3	0.4	0.3
queens	[w,mg]	0.8	0.7	0.6	0.9
queens	[w,ph]	0.9	0.8	0.7	0.7
rooks	[b,eg]	1.0	0.9	1.0	0.9
rooks	[b,mg]	1.0	1.0	0.9	1.0
rooks	[b,ph]	0.9	1.0	1.0	0.9
rooks	[t,eg]	0.6	0.5	0.3	0.7
rooks	[t,mg]	0.7	0.8	0.7	0.7
rooks	[t,ph]	0.7	0.5	0.5	0.8
rooks	[w,eg]	1.0	1.0	0.9	1.0
rooks	[w,mg]	1.0	1.0	1.0	1.0
rooks	[w,ph]	0.9	0.9	0.8	0.9
scale factor		0.7	0.8	0.8	0.6
space	[b,eg]	0.3	0.3	0.4	0.3
space	[b,mg]	1.0	1.0	1.0	1.0
space	[b,ph]	1.0	1.0	0.9	1.0
space	[t,eg]	0.3	0.3	0.4	0.3
space	[t,mg]	0.8	0.8	1.0	0.9
space	[t,ph]	0.9	0.9	0.7	0.9
space	[w,eg]	0.3	0.3	0.4	0.3
space	[w,mg]	1.0	1.0	0.9	1.0
space	[w,ph]	1.0	1.0	1.0	1.0
threats	[b,eg]	1.0	1.0	0.9	0.9
threats	[b,mg]	1.0	0.7	0.8	1.0
threats	[b,ph]	1.0	0.8	0.9	1.0
threats	[t,eg]	0.7	0.9	0.7	0.7
threats	[t,mg]	0.4	0.7	0.4	0.3
threats	[t,ph]	0.4	0.6	0.7	0.4
threats	[w,eg]	0.9	0.9	0.8	0.9
threats	[w,mg]	1.0	0.8	0.9	0.9
threats	[w,ph]	1.0	0.9	1.0	1.0
total score		0.7	0.8	0.4	0.6
total	[t,eg]	0.5	0.7	0.5	0.6
total	[t,mg]	0.8	0.6	0.6	0.8
total	[t,ph]	0.8	0.7	0.9	0.8

375 Further descriptions of the STS concepts can be found at this website.

Table S6. Concept constraint Satisfaction Results for STS Dataset

Concept	layer 19	layer 20	layer 21	layer 23
Undermine	1.0	1.0	1.0	0.9
Open files	1.0	1.0	1.0	1.0
Knight outposts	1.0	1.0	1.0	0.9
Square vacancy	1.0	1.0	1.0	1.0
Bishop vs. knight	1.0	0.9	0.9	1.0
Recapture	1.0	1.0	1.0	1.0
Offer of simplification	0.9	1.0	1.0	0.8
fgh-pawn	1.0	1.0	1.0	1.0
abc-pawn	1.0	1.0	1.0	1.0
Simplification	0.9	1.0	1.0	0.9
King activity	1.0	1.0	1.0	1.0
Pawn push center	1.0	1.0	1.0	1.0
7th rank	1.0	1.0	1.0	1.0
Avoid exchange	1.0	1.0	1.0	1.0

Table S7. Concept constraint Satisfaction Results for Opening Concept Configurations

Hyperparamters	layer 19	layer 20	layer 21	layer 23
single	1.00 (0.00)	1.00 (0.00)	1.00 (0.00)	1.00 (0.00)
both (k=10)	1.00 (0.00)	0.99 (0.00)	0.99 (0.01)	1.00 (0.00)
both (k=5)	1.00 (0.00)	1.00 (0.00)	1.00 (0.00)	1.00 (0.00)

Table S8. Concept constraint Satisfaction Results for Opening Concepts

Concept	layer 19	layer 20	layer 21	layer 23
English	1.0	1.0	1.0	1.0
Dutch	1.0	1.0	1.0	1.0
Scandinavian	1.0	1.0	1.0	1.0
Sicilian	1.0	1.0	1.0	1.0
Najdorf	1.0	1.0	1.0	1.0
French	1.0	1.0	1.0	1.0
Tarrasch	1.0	1.0	1.0	1.0
Winawer	1.0	1.0	1.0	1.0
Grünfeld	1.0	1.0	1.0	1.0

6. Further details: convex optimisation formulation for concepts

This section provides further details on our convex optimisation framework to find concepts. Subsection A describes how we set the dynamic concept hyperparameters. §C explains how the convex optimisation formulations were implemented for the different datasets.

A. Dynamic concept hyperparameters. As AZ learnt to play chess through self-play, the latent representations alternates between the player's and the opponent's perspective within a rollout. For concepts, we may want to find a concept that influences a single player or both players. Therefore, we consider two different ways of using rollouts.

1. For a rollout $\{\mathbf{z}_t\}_{t=0}^{T}$, we use every other latent representation, i.e., $\{\mathbf{z}_{2t}\}_{t=0}^{\text{floor}(T/2)}$, to find concepts for a single player (i.e., for the player to move, or their opponent). These concepts are referred to as 'single'.

2. We use every latent representation to find concepts for both players, i.e. $\{\mathbf{z}_t\}_{t=0}^{T}$. These concepts will be referred to as 'both'.

For the rollout depth used in our dynamic concept formulations, we consider $T = 5$ and $T = 10$. For the subpar variations, we required AZ to estimate a minimum value difference of 0.20 and/or a visit count difference of 10% (of the most visited move).

B. Datasets. We use labelled and unlabelled datasets to construct concept constraints for the convex optimisation formulation. We use a different convex optimisation formulation for each concept we want to discover. Therefore, for each concept, we need a set of chess positions $\mathbb{X}^+$ that contains a concept. We leverage educational resources designed to teach humans chess, which contains themed chess puzzles: positions designed to encapsulate single important concepts and test the degree to which a chess player can deploy them in realistic situations. We go beyond chess puzzles, delving into chess positions arising from different openings and searching for AZ-specific concepts to find new ones.

Factors in datasets. The datasets we use vary in the following ways:

- **Concept type** e.g., the concepts can be strategic or tactical (see (8) and (9) for a further explanation on strategy and tactics), or correspond to different periods of the game (opening/middle game/endgame).
- **Degree of human knowledge** some datasets contain human games while others contain AZ's games. The degree of human knowledge (or style of play) may vary across the datasets.
- **Complexity** some concepts are elementary (i.e., can be learned by beginner-level chess players), whereas others are highly complex (i.e., can only be understood by top-level grandmasters).

Below, we briefly describe the different data sets used

1. **Piece dataset** We construct a labelled dataset that contains paired chess positions that either contain or do not contain a chess piece. These simple concepts indicate the presence of a piece - queen, rook, bishop, knight, or pawn. We exclude the concept of 'king' as this piece is always present in a chess game.
2. **Stockfish dataset** We construct a labelled dataset for each concept in the Stockfish engine (10). Each dataset contains two sets of chess positions - with and without the concept. Humans use Stockfish concepts in chess position evaluation (such as piece placement, open files, etc.)
3. **STS puzzles dataset** This is a labelled dataset containing 15 different categories (see (11) for further details). These puzzles capture different types of strategic themes.
4. **Chess openings** (e.g., concepts in Grünfeld vs. Najdorf vs. Queens Gambit) Openings (i.e., the first couple of moves) determine the pawn structure that acts as the backbone of a chess position – it determines crucial aspects of the game, such as optimal piece placement, square weaknesses and strengths, and more generally, plans. We use the Encyclopedia of Chess Openings (ECO) to create a set of labelled chess opening positions (12). For each chess position, we use AZ's MCTS rollouts to construct sequences of the opening moves.
5. **AZ self-play games** We construct an unlabelled dataset. We sample $30,000$ chess positions from AZ's games. To ensure that the chess positions we analyze contain complex concepts, we only select chess positions where two versions of AZ at different points in training select a different move. These versions differ by 75 Elo points.

Table S9 briefly summarises the different datasets.

Table S9. Datasets Summaries: from concepts more known to humans (top rows) to AZ (bottom rows)

Name	Concept Type		Type of Knowledge	Complexity
	Strategic vs. Tactical	Game Phase		
Piece	N/A	All	Human	Low
Stockfish	Both	All	Human	Varies
STS	S	Middle/End	Human	Medium
Opening	S	Begin	Human/AZ	Varies
AZ	Both	All	AZ	High

The human crafted datasets are used to (1) validate the convex optimisation framework and (2) explain the novel concepts by relating discovered concepts to something humans know (by learning a graph).

C. Convex formulation for different datasets. In this section, we provide further details on the convex optimisation formulations for each dataset. Unless otherwise stated, we use a 90-10 train-test split for the supervised datasets. In our analysis, we generally consider layers 19, 20, 21 and 23. However, as layers 19 and 23 showed the largest difference in rank compared to human data in our analysis §2, we use layer these layers to discover the concepts shown to grandmasters.

Piece. We artificially created a piece dataset by sampling chess positions from grandmaster games sampled from (13). For each concept, we sampled 100 chess positions with the concept to create the set $\mathbb{X}^+$ and then created 100 chess positions without the concept to create the set $\mathbb{X}^-$(by removing the piece).

We formulated the convex optimisation problem as follows. For pair of chess positions, $\mathbf{x}_i^+ \in \mathbb{X}^+$ and $\mathbf{x}_i^- \in \mathbb{X}^-$, we find the corresponding latent representations in layer l to create $\mathbb{Z}_l^+$ and $\mathbb{Z}_l^-$, respectively. Using these latent representations, we can search for the piece presence concept using the following formulation

$$\min \quad \|\mathbf{v}_{c,l}\|_1 \tag{5}$$

$$\text{such that} \quad \mathbf{v}_{c,l}^\top \mathbf{z}_{i,l}^+ \geq \mathbf{v}_{c,l}^\top \mathbf{z}_{i,l}^- \quad \text{for all} \quad \mathbf{z}_{i,l}^+ \in \mathbb{Z}_l^+, \mathbf{z}_{i,l}^- \in \mathbb{Z}_l^- . \tag{6}$$

Note that this is the same formulation as for static concepts more generally (see §3).

Stockfish. Following (7), we extract the concepts encoded in the Stockfish engine. We use the Stockfish engine code to extract a concept value for each position. We sampled $30,000$ chess positions from (13). For each of these chess positions, we found the chess positions that were in the top 5th percentile (assumed to contain the concept) to construct $\mathbb{X}^+$ and the bottom 95th percentile (assumed not to contain the concept) for the concept score to construct $\mathbb{X}^-$. We randomly paired the chess positions that contained the concept, $\mathbf{x}_i^+$, with chess positions that scored low for the concept $\mathbf{x}_j^-$. Similarly to before, we extracted the latent representations for each concept. Then, using the formulation for static concepts, we found the concept vector using the following formulation

$$\min \quad \|\mathbf{v}_{c,l}\|_1 \tag{7}$$

$$\text{such that} \quad \mathbf{v}_{c,l}^\top \mathbf{z}_{i,l}^+ \geq \mathbf{v}_{c,l}^\top \mathbf{z}_{j,l}^- \quad \text{for all} \quad i : \mathbf{x}_i \in \mathbb{X}^+, \; j : \mathbf{x}_j \in \mathbb{X}^- . \tag{8}$$

Strategic. We use the strategic test suite to extract strategic concepts (11). In this dataset, there are 15 different concepts. We omit the 12th concept due to irregular data formatting. The remaining concepts are undermine, open files, knight outposts, square vacancy, bishop vs. knight, recapture, offer of simplification, fgh-pawn, abc-pawn, simplification, king activity, pawn push center, 7th rank, avoiding an exchange (see 11, for further details).

Each concept has a set of 100 chess positions, $\mathbb{X}$, and the solution (move) requires applying a strategic concept in each chess position. In our analysis, we run MCTS on the chess position and store the search statistics. We store the optimal trajectory for each chess position $\mathbf{x}_i$, denoted as $\mathbb{X}_{i,\leq T}^+$, where T is the maximum rollout depth. Similarly we select a subpar rollout $\mathbb{X}_{i,\leq T}^-$. To find the subpar rollout, we find a rollout in the MCTS tree with the most visits where (1) the estimated difference in value is at least 0.2, and (2) the visit difference is at least 10%. As in the main text, for $\mathbb{X}_{i,\leq T}^+$ and $\mathbb{X}_{i,\leq T}^-$, we find the corresponding latent representations in layer l: $\mathbb{Z}_{i,\leq T}^+$ and $\mathbb{Z}_{i,\leq T}^-$, respectively. Then, we can find the concept vector using the dynamic concept formulations (see §2) as follows

$$\min \quad \|\mathbf{v}_{c,l}\|_1 \tag{9}$$

$$\text{such that} \quad \mathbf{v}_{c,l}^\top \mathbf{z}_{i,t}^+ \geq \mathbf{v}_{c,l}^\top \mathbf{z}_{i,t}^- \quad \text{for all} \quad t \leq T, \; i : \mathbf{x}_i \in \mathbb{X}. \tag{10}$$

In our analysis, we use a maximum depth of $T = 5$.

Openings. For the openings, we focus on the English, Dutch, Scandinavian, Najdorf, French, Tarrasch, Winawer, Ruy Lopez, Grünfeld, King's Indian, Queen's Gambit Declined and Queen's Gambit Accepted. We consider a subset of all openings due to computational costs. For each opening, we use the encyclopedia of chess openings to find relevant starting chess positions to construct $\mathbb{X}^+$ (see the encyclopedia of chess openings). For each chess position, we ran MCTS to obtain the search statistics and used the formula in Equation 3-4to find a concept for each opening. Further, we create a concept set $\mathbb{X}^+$ for each ECO index belonging to one of the aforementioned openings.

AZ Games. We simulated $1,308$ games. Conditional on hardware, AZ's play is deterministic (after training). To create diverse games, we sample different starting chess positions. We use the ECO to find starting chess positions (see the encyclopedia of chess openings) and we simulate games from these initial chess positions. For each chess position, we ran MCTS to obtain the search statistics.

We leverage AZ's training history to find interesting chess positions. We select a version of AZ that is 75 Elo points weaker than the final model. To construct $\mathbb{X}^+$, we run through each game and select chess positions where the two AZ versions choose a different move. Using this approach, we constructed a dataset with $3,974$ chess positions and used the formulation provided in Equation 3-4 to find concept vectors.

Table S10. Hyperparameter selection for β

	0.05	0.01	0.025	0.05	0.1	0.25	0.5	1.0	2.0
Layer 18	58.42	58.58	58.50	58.42	58.17	58.17	57.42	56.75	55.00
Layer 19	60.17	60.42	60.42	60.17	60.08	60.17	59.75	58.00	55.83
Layer 23	58.83	59.42	59.33	58.83	58.58	56.25	53.92	47.75	40.33

Other Implementation Details. We solve the convex optimisation problem using a standard solver in the package `cvxpy` (14, 15).

D. Beta hyperparameter tuning. In this section, we provide the validation values for the concept amplification experiments in §C.1. For the values in Table S10, we randomly chose 2 concept sets from the STS dataset and estimated the amplification results for different values of β. Overall, we observe that the results are not very sensitive to the value of β.

7. Teachability implementation

In this section, we provide further details on the implementation of teachability. We assume that we have a concept vector $\mathbf{v}_{c,l}$ that was found in layer l.

Prototypes To construct prototypes, we sample 30,000 chess positions from AZ games. For each concept, we find chess positions for which $\mathbf{v}_{c,l}^{\top}\mathbf{z}_{i,l}$ is in the top 2.5%, and store these as prototypes $\{\mathbf{x}_{1,l}, \ldots, \mathbf{x}_{n,l}\}$. For dynamic concepts, we found prototypes using MCTS statistics. For each chess position, we ran MCTS. Next, we found the optimal and subpar line (similar to the convex optimisation formulation constraint). For a prototype x_i, we required that $\mathbf{v}_{c,l}\mathbf{z}^{+}_{i,t,l} \geq \mathbf{v}_{c,l}\mathbf{z}^{-}_{i,t,l}$ for all t.

Student and Teacher We use AZ as the teacher network. For the student network, we want to find an agent that does not know the concept but does understand chess. As chess is a complex game, we cannot train an agent from scratch (using only the curriculum). Instead, we take a training checkpoint of AZ and estimate its knowledge of the concept using

$$T = \sum_{\mathbf{x}_i \in \mathbb{X}} \mathbb{1}[\mathrm{argmax}(\pi^{\mathrm{s}}(\mathbf{x}_i)), \mathrm{argmax}(\pi^{\mathrm{t}}(\mathbf{x}_i))], \quad [11]$$

where $\pi^{\mathrm{s}}()$ is the student policy and $\pi^{\mathrm{t}}()$ is the teacher policy. This measures how often the teacher and student agree on the best move. We select the student as the latest checkpoint for which the top-1 policy overlap is less than 0.2.

Curriculum We use the prototypes as a curriculum. We train the student network by minimizing the KL divergence between the policies, $\sum_{\mathbf{x}_i \in \mathbb{X}_{train}} \mathrm{KL}[\pi^{\mathrm{t}}(\mathbf{x}_i), \pi^{\mathrm{s}}(\mathbf{x}_i)]$. We use the Adam optimiser (16) with learning rate 1×10^{-4}. As we have several concepts, we train each student for 50 epochs, as we find that this is sufficiently indicative of performance if we train for longer.

Benchmark We benchmark the performance by comparing it to a student network trained on a random concept and evaluated on (1) the concept data and (2) the random data. A random concept is a vector with the same shape as the latent dimension and sampled from a standard normal distribution. The vector is then re-scaled by a factor of $1/n$ where n is the number of hidden units in the layer. We use the random concept vector to find random prototypes. The randomly sampled prototypes are still informative as they are paired with AZ's policy.

8. Graph analysis

We build a directed graph between AZ's concept vectors and human-labelled concept vectors to improve our understanding of the new, unlabelled concepts. In our work, we found a set of concept vectors $\mathbb{V}$ (see §2). Assume we have two different concept vectors $\mathbf{v}_{c,l}, \mathbf{v}_{k,l} \in \mathbb{V}$, where $c \neq k$. Our idea is simple: if concepts $\mathbf{v}_{c,l}$ and $\mathbf{v}_{k,l}$ are related, they will arise in the same chess positions. Let $s_{c,i,l}$ denote whether concept $\mathbf{v}_{c,l}$ is present in a chess position i using the latent representation $\mathbf{z}_{i,l}$ in layer l. For each concept c, we estimate the regression model (similar to (17))

$$s_{c,i,l} = \sum_{l \in 19,23} \sum_{k \in \mathbb{V} \setminus c} \beta_{k,l} s_{k,i,l} + \lambda \|\beta_{k,l}\|^2 \quad \text{for all} \quad i : \mathbf{x}_i \in \mathbb{X}, \quad [12]$$

where $\beta_{k,l}$ is the regression coefficient and $\mathbb{X}$ is a set of positions (we will elaborate on this later). If $\beta_{k,l}$ is significant at a 5% level, we add a directed edge from $\mathbf{v}_{k,l}$ to $\mathbf{v}_{c,l}$. We use a regression as a concept $\mathbf{v}_{c,l}$ may be important for $\mathbf{v}_{k,l}$ but not vice-versa.

The next question is: how do we define $s_{c,i,l}$? Following the ideas in used to discover concepts, we assume that product $\mathbf{v}_{c,l}^{\top}\mathbf{z}_{i,l}$ is higher for $\mathbf{z}_{i,l}$, corresponding to positions that contain concept c. Therefore, for static concepts, we measure the concept presence as

$$s^{\mathrm{static}}_{c,i,l} = \mathbf{v}_{c,l}^{\top}\mathbf{z}_{i,l} \ , \quad [13]$$

where $\mathbf{z}_{i,l} \in \mathbb{Z}^s_l$, and $\mathbb{Z}^s_l$ is a set of latent representations in layer l corresponding to a set of chess positions $\mathbb{X}^s$. We sample 2,000 positions from human games to create $\mathbb{X}^s$ as static concepts are found using human-labelled positions.

For dynamics concepts, we estimate the concept relevance as

$$s^{\mathrm{dynamic}}_{c,i,l} = \sum_{\mathbf{z}_{i,l,t} \in \mathbb{Z}_{i,l,\leq T}} \mathbf{v}_{c,l}^{\top}\mathbf{z}_{i,l,t} \quad [14]$$

where $\mathbf{z}_{i,l,t} \in \mathbb{Z}_{i,l,\leq T}$ are the latent representations corresponding to the AZ's chosen MCTS rollout for a starting chess position $\mathbf{x}_i$ and maximum depth T, and $x_i \in \mathbb{X}^d$. We sample 2,000 positions from AZ's games to construct $\mathbb{X}^d$, as the dynamic concepts are predominantly found in AZ's games.

As we want to analyze the relationship between static and dynamic concepts, we estimate $s^{\mathrm{static}}_{c,i,l}$ and $s^{\mathrm{dynamic}}_{c,i,l}$ for each $\mathbf{v}_{c,l}$.

In our analysis, we only keep human concepts of high quality, i.e., that have a concept constraint score of higher than 0.90. Further, for our analysis to be stable, we removed highly correlated variables, i.e. variables that had a correlation coefficient higher than 0.99 (18–20) dropping 580 concepts (out of 1,371).

Graph Summary. The graph is dense – the graph contains 29% $(180,609/625,681)$ of the possible directed edges. As the graph is very dense, we do not provide the entire graph. Instead, we provide parts of the graph in §1 when presenting examples of AZ's concepts.

Graph Verification. To verify the graph, we run an experiment to test whether two concepts with an edge contain related knowledge. If a model learns a concept c, this should improve the model's performance on another related concept c_e more than on an unrelated concept c_n. For a concept c, let $\mathbb{C}_e$ denote the set of concepts with an edge and $\mathbb{C}_n$ denote the set of concepts with no edge in the graph. Following the teachability procedure in §2, we train a student model using prototypes of concept c. Next, using Equation 5, we evaluated student's performance on:

- concepts with an edge $\mathbb{C}_e$; we denote the performance by T_{c,c_e}
- concepts without an edge (to $\mathbf{v}_{c,l}$) $\mathbb{C}_n$; we denote the performance by T_{c,c_n}.

If the graph correctly captures the relationships between concepts, then we expect that a model trained on c performs better on $\mathbb{C}_e$ than $\mathbb{C}_n$, i.e. $T_{c,c_e} > T_{c,c_n}$.

However, we must consider that the concepts in $\mathbb{C}_e$ may be inherently easier to learn than those in $\mathbb{C}_n$. To account for this, we train a model on a random set of data (as in §2), which we use as a benchmark. We estimate the performance of this model on $\mathbb{C}_e$ and $\mathbb{C}_n$, which we denote by T_{r,c_e} and T_{r,c_n}, respectively.

If our graph accurately captures the underlying relationships, we expect that $T_{c,c_e} - T_{r,c_e} > T_{c,c_n} - T_{r,c_n}$. Figure S24 shows training curves for two concepts each of which with 5 related concepts and 5 unrelated concepts.

Fig. S24. Teachability score on related and unrelated concepts

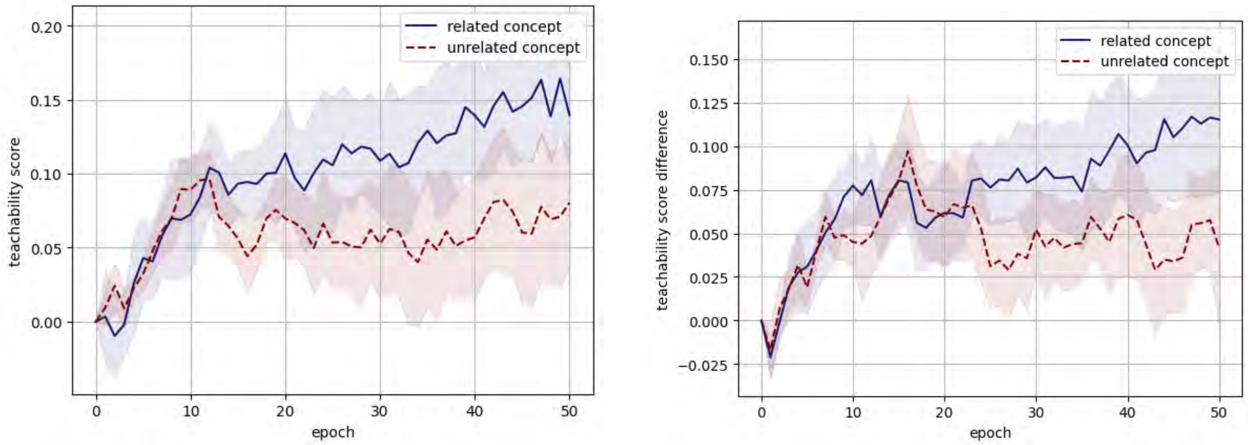

We find that the performance on related concepts is significantly better than unrelated concepts at a 5% significance level. This suggests that the graph structure may accurately capture the relationship between concepts.

9. Human experiments

Recruitment. We recruited four chess players based on their Elo rating. All participants hold the grandmaster title, and currently/previously the World Champion title; one of our participants is rated 2600-2700, and three are rated 2700-2800.

Experiment Setup Human evaluation with grandmasters follows three phases, similar to teachability (§2):

- **Phase 1: Measuring baseline performance.** Each grandmaster provides solutions for a set of provided puzzles corresponding to a set of concepts. This phase determines the baseline performance: the number of puzzles in which the chess grandmaster gets the continuation correct *before* the learning phase.
- **Phase 2: Learning from AZ's calculations.** The same puzzles as in Phase 1 are shown to chess grandmasters alongside the associated AZ's suggested top line based on MCTS calculations for each puzzle. This serves as the simplest way of teaching.
- **Phase 3: Measuring final performance.** Grandmasters are tasked with providing solutions for a test set of unseen puzzles sampled from the same concepts they have seen in Phase 1. We compute the grandmasters' accuracy on the puzzle test set and compare it to their performance on the puzzle training set in Phase 1 to measure whether their performance changes.

The train-test split of concept puzzles is random.

Experiment Instructions. Each grandmaster was asked to spend two hours on Phase 1, one hour on Phase 2 and two hours on Phase 3. We ask the grandmasters to provide (1) the move they would play or their ranked candidate moves and (2) a thought record – the idea is to capture any thoughts about the chess position. The grandmasters were sent the chess positions to solve at home, in their own time. We explained that the chess positions could vary in nature. The chess positions could be better, equal or worse for the player to move. Similarly, the continuation may require calculation or finding a general plan.

Evaluation. We evaluated how often the grandmasters find the move selected by AZ. Note that if grandmasters made the right move but incorrect reasoning appeared in their free-form comments, we counted this as an incorrect answer.

Prototype Filtering. To ensure the quality of the prototype selection, we filter them according to the following criteria:

- **Quality of the value estimate.** We ensure that the AZ value estimate is close to the correct assessment of the prototype by running self-play and computing the expected score. If the expected score and the value estimate are in concordance, the prototype chess position is kept, otherwise, it is discarded from consideration for the human study.
- **Chess position complexity.** For the concepts to be sufficiently complex to be of interest to the top grandmasters, we use prototypes where the policies of the 512K step checkpoint and fully trained models disagree on their top move. The 512K checkpoint model is 75 Elo points weaker than the final model, and therefore, if the policies differ, AZ learned the continuation during a late stage of training when it was already strong.
- **Solution complexity.** We manually remove *trivial* chess positions where the solution is theoretically known (e.g., present as an entry in pre-computed tablebases such as the Syzygy tablebase (21)). Tablebases are sets of chess positions where the ground truth evaluation (outcome with ideal play) is known.
- **Reliability.** We reject chess positions where AZ's limited compute budget may lead to an unreliable chess position evaluation (i.e., where we observe abrupt changes in the predicted outcome). Therefore, we require that the evaluation stays approximately consistent (i.e., the predicted outcome (win/loss/draw) does not change) throughout the provided lines.

We did not filter based on the difference in the value or the policy probability mass of the optimal move compared to other moves. The reasons are that (1) AZ's value estimate is noisy, and (2) either filter could remove potentially interesting chess positions. For example, requiring a small entropy and large value estimate difference (between moves) would result in predominantly tactical chess positions, thereby omitting interesting strategic puzzles.

While there was some overlap between study participants in terms of puzzles shown, different participants were shown different concepts.

AZ's calculations. In the second stage of the human experiment, we provide part of the MCTS statistics. We ran MCTS without a depth limit for a maximum of $10,000$ simulations. We pruned the MCTS tree to avoid providing too many lines, or lines that were insufficiently explored. We provided the main line (most frequently visited), second and third moves, ranked according to visits. We did this for depth $t \leq 2$.

Factors that affect performance

- **Variability in difficulty and quality.** We filter prototypes (as described in §2) to ensure quality and complexity. However, the difficulty and quality of puzzles players received may vary across puzzles.
- **Variability in teachability.** While we filter based on teachability (as described in §2), the teachability metric is based on teaching the concept to another AI agent, which may be inherently different from teaching humans.
- **Overthinking.** We observed that grandmasters often mention AZ's move in Phase 3 in their free-form comments but ultimately did not choose the move (which was not counted as 'correct'). We speculate that this may be because players are more familiar with existing strategies in their decision process, despite having learnt the concept.

References

1. N Fiekas, python-chess (2023).
2. D King, *How to Win at Chess.* (Everyman Chess), (2000).
3. R Brunia, C van Wijgerden, *Steps Method.* (Self-published), (2021).
4. C Hansen, *Back to Basics: Chess Openings.* (Russel Enterprises), (2021).
5. FIDE, Fide handbook c.02 (2019) [Online; accessed 13-October-2023].
6. J Schrittwieser, et al., Mastering Atari, Go, chess and shogi by planning with a learned model (2019).
7. T McGrath, et al., Acquisition of chess knowledge in alphazero. *Proc. Natl. Acad. Sci.* **119**, e2206625119 (2022).
8. Wikipedia contributors, Chess strategy (2023) [Online; accessed 13-October-2023].
9. Wikipedia contributors, Chess tactic (2023) [Online; accessed 13-October-2023].
10. Stockfish Community, Stockfish Chess (https://stockfishchess.org/) (2018) Accessed: 23 July 2023.
11. D Corbit, S Natarajan, F Mosca, Strategic test suite (2014).
12. LiChess, Encyclopedia of chess openings (2023) [Online; accessed 1-May-2023].
13. ChessBase, Chessbase Mega Database (database.chessbase.com) (2021).
14. S Diamond, S Boyd, CVXPY: A Python-embedded modeling language for convex optimization. *J. Mach. Learn. Res.* **17**, 1–5 (2016).
15. A Agrawal, R Verschueren, S Diamond, S Boyd, A rewriting system for convex optimization problems. *J. Control. Decis.* **5**, 42–60 (2018).
16. DP Kingma, J Ba, Adam: A method for stochastic optimization (2017).
17. N Meinshausen, P Bühlmann, High-dimensional graphs and variable selection with the lasso. *The Annals Stat.* **34** (2006).
18. PJ Robinson, Fitting equations to data: Computer analysis of multifactor data for scientists and engineers. *JMR, J. Mark. Res. (pre-1986)* **11**, 346 (1974).
19. RD Snee, Some aspects of nonorthogonal data analysis: Part i. developing prediction equations. *J. Qual. Technol.* **5**, 67–79 (1973).
20. RD Snee, DW Marquardt, Comment: Collinearity diagnostics depend on the domain of prediction, the model, and the data. *The Am. Stat.* **38**, 83–87 (1984).
21. Bojun Guo, Syzygy tablebase (2023) [Online; accessed 23-July-2023].
