# Supplementary material for: Bridging the human–AI knowledge gap through concept discovery and transfer in AlphaZero
Source: Proc Natl Acad Sci U S A. 2025 Mar 26;122(13):e2406675122. doi: 10.1073/pnas.2406675122 (PMC12002201; doi:10.1073/pnas.2406675122)
Supplement: Supplementary file 2 — Dataset S01 (RTF) [file pnas.2406675122.sd01.rtf]

[StudyName "Appendix"][ChapterName "Position 1"][FEN "r1bqr1k1/pp3ppp/5n2/3pb3/8/1PN1P3/PB1QBPPP/R4RK1 b - - 2 13"]{ Player 1, Concept 2, Phase 1 }13... Be6 *[StudyName "Appendix"][ChapterName "Position 2"][FEN "r1bq1rk1/pp2bppp/2np1n2/2p1p3/2P5/1PN1PNP1/P1QP1PBP/R1B2RK1 b - - 0 9"]{ Concept 2, Player 1, Phase 1 }9... Re8 *[StudyName "Appendix"][ChapterName "Position 3"][FEN "rn1q1rk1/pb2ppbp/3p1np1/2pP4/1pP1PB2/P2B1N2/1PQ2PPP/RN2K2R w KQ - 1 10"]{ Concept 2, Player 1, Phase 1 }10. O-O *[StudyName "Appendix"][ChapterName "Position 4"][FEN "rn2k2r/3pppbp/b4np1/q1pP4/5P2/2N5/PP1BP1PP/R2QKBNR w KQkq - 3 9"]{ Concept 2, Player 1, Phase 1 }9. e4 *[StudyName "Appendix"][ChapterName "Position 5"][FEN "rn1qkbnr/pp3ppp/2p1p3/3p4/2P3b1/1P2PN1P/P2P1PP1/RNBQKB1R b KQkq - 0 5"]{ Concept 3, Player 1, Phase 1 }5... Bf5 *[StudyName "Appendix"][ChapterName "Position 6"][FEN "1rbq1rk1/2p2ppp/p1np1n2/1pb1p3/2P1P3/2NP2PP/PP2NPBK/1RBQ1R2 b - - 4 11"]{ Player 1, Concept 3, Phase 1 }11... h6 *[StudyName "Appendix"][ChapterName "Position 7"][FEN "r1r2nk1/p1b1q1pp/1pp1pn2/2Pp1p2/PP1P4/3P2P1/2QN1PBP/1RB2RK1 w - - 1 16"]{ Player 1, concept 3, Phase 1Player 3, Concept 9, Phase 1 }16. Nf3 *[StudyName "Appendix"][ChapterName "Position 8"][FEN "1k1r1r2/ppb2ppp/2n5/3p3P/3P4/3BB1P1/PP3P2/1K1R3R b - - 2 20"]{ Player 1, Concept 3, Phase 1 }20... f5 *[StudyName "Appendix"][ChapterName "Position 9"][FEN "r1bqk2r/ppp2pbp/3p1np1/3Pp3/2PnP3/P1N2N1P/1P3PP1/R1BQKB1R w KQkq - 1 9"]{ Concept 6, Player 1, Phase 1 }9. Bg5 *[StudyName "Appendix"][ChapterName "Position 10"][FEN "R7/1pNq1r2/1P1p2nk/2nP1ppp/4p3/4P1P1/5PBP/1Q4K1 w - - 8 31"]{ Player 1, Concept 6, Phase 1 }31. Qa1 *[StudyName "Appendix"][ChapterName "Position 11"][FEN "1k1r3r/1p1n1p2/p1p1pnp1/q1Pp4/3P1PPP/1PN5/P1Q1B3/1K1R3R w - - 0 21"]{ Player 1, Concept 6, Phase 1Player 2, Concept 1, Phase 1 }21. Qd2 *[StudyName "Appendix"][ChapterName "Position 12"][FEN "r2q1rk1/1p1n2p1/2pbp2p/p2p1p2/P1PP4/1P2P1P1/1B1N1PKP/R2QR3 b - - 0 16"]{ Player 1, Concept 6, Phase 1 }16... Qe7 *[StudyName "Appendix"][ChapterName "Position 13"][FEN "q4rk1/3nppbp/b2p1np1/1NpP4/P3P3/3BBN2/2Q2PPP/5RK1 b - - 4 16"]{ Player 1, concept 2, phase 2 }16... Rb8 *[StudyName "Appendix"][ChapterName "Position 14"][FEN "r1b1k2r/1ppnqp2/p2p1npp/3Pp3/2P1P1P1/2N1QN1P/PPB2P2/1K1R3R w kq - 0 17"]{ Player 1, concept 2, phase 2Player 3, concept 8, phase 2 }17. Ne1 *[StudyName "Appendix"][ChapterName "Position 15"][FEN "r1bqkb1r/ppp2p2/4p3/3pPpNp/3P1P2/2P1Q3/PP1N2PP/R4RK1 b kq - 1 15"]{ Player 1, concept 2, phase 2 }15... Be7 *[StudyName "Appendix"][ChapterName "Position 16"][FEN "r4rk1/3nppbp/b2p1np1/q1pP4/8/2N2NP1/PPQ1PPBP/R1B2RK1 w - - 7 12"]{ Player 1, concept 2, phase 2 }12. Bd2 (12. Rd1) *[StudyName "Appendix"][ChapterName "Position 17"][FEN "r2q1rk1/pp1n1pp1/2pbpn1p/3p4/2PP2b1/1P3NP1/PB1NPPBP/R2QR1K1 b - - 1 10"]{ Player 1, Concept 3, Phase 2Player 3, Concept 7, Phase 1 }10... Bf5 *[StudyName "Appendix"][ChapterName "Position 18"][FEN "r2q1rk1/1b1nppbp/pp3np1/2pp2B1/P2P4/1QP1PN1P/1P1NBPP1/R4RK1 b - - 1 11"]{ Player 1, Concept 3, Phase 2 }11... Re8 *[StudyName "Appendix"][ChapterName "Position 19"][FEN "r1bqr1k1/ppp1pnbp/2np2p1/1B1N1p2/3P3B/2P1P2N/PP3PPP/R2QK2R w KQ - 1 9"]{ Player 1, Concept 3, Phase 2Player 3, Concept 8, Phase 1 }9. Ndf4 *[StudyName "Appendix"][ChapterName "Position 20"][FEN "r1bq1rk1/1pp1npbp/3p2p1/p2Pp3/4P3/2NP2P1/PP3PBP/1RBQ1RK1 b - - 0 13"]{ Player 1, Concept 3, Phase 2Player 3, Concept 9, Phase 2 }13... c6 *[StudyName "Appendix"][ChapterName "Position 21"][FEN "1r2r1k1/1b2qpp1/p2p1n1p/2p1p3/2PbP3/1P1Q2PP/P2BNPBK/4RR2 b - - 1 22"]{ Player 1, Concept 6, Phase 2Player 2, Concept 4, Phase. 1 }22... Bc6 *[StudyName "Appendix"][ChapterName "Position 22"][FEN "k1r3r1/pb6/1p2Nb2/3p1Pp1/1PqN1Q1p/P2R4/6PP/3R2K1 w - g6 0 28"]{ Player 1, Concept 6, Phase 2Player 2, Concept 4, Phase 2 }28. Qd2 *[StudyName "Appendix"][ChapterName "Position 23"][FEN "1k2rr2/1pb2p1R/p1n3p1/3p4/3P4/4B1P1/PPB2P2/1K1R4 w - - 2 24"]{ Player 1, Concept 6, Phase 2Player 2, Concept 1, Phase 2 }24. Bb3 *[StudyName "Appendix"][ChapterName "Position 24"][FEN "1r4k1/1rn1ppbp/q2p1np1/2pP4/P3P3/1PN1B1P1/2Q1NPKP/1R1R4 b - - 4 18"]{ Player 1, Concept 6, Phase 2 }18... Rb6 *[StudyName "Appendix"][ChapterName "Position 25"][FEN "r4rk1/pp1b2pp/2nqpn2/3p4/1PP5/P2B1N2/5PPP/RN1QR1K1 w - - 1 19"]{ Player 2, Concept 5, Phase 1 }19. Nbd2 *[StudyName "Appendix"][ChapterName "Position 26"][FEN "r1b3k1/1pB2pbp/2p3p1/p1n5/P1P5/2N3P1/1PK1PPBP/7R b - - 6 17"]{ Player 2, Concept 4, Phase 1 }17... Bxc3 *[StudyName "Appendix"][ChapterName "Position 27"][FEN "r2q1rk1/pb2bppp/1p2pn2/2p1P3/2NP1n2/1P3N2/PB3PPP/R2QRBK1 b - - 0 17"]{ Player 2, Concept 4, Phase 1 }17... Ng4 *[StudyName "Appendix"][ChapterName "Position 28"][FEN "r2qr1k1/1bp2ppp/p3pb2/1p2N3/1B1P2Qn/1P1B4/P1P2PPP/3RR1K1 w - - 1 18"]{ Player 2, Concept 5, Phase 1 }18. f3 *[StudyName "Appendix"][ChapterName "Position 29"][FEN "8/1p4p1/4p1Np/P1r5/2k4P/1n4P1/6K1/1R6 w - - 1 54"]54. Re1 *[StudyName "Appendix"][ChapterName "Position 30"][FEN "8/5p1k/7p/5p2/5P2/8/r6r/2R2KQ1 w - - 32 65"]{ Player 2, concept 1, phase 1 }65. Rd1 *[StudyName "Appendix"][ChapterName "Position 31"][FEN "4k3/p5p1/1p2P2p/1P3P1P/PnB2P2/2p1K3/8/8 w - - 2 43"]{ Player 2, Concept 1, Phase 1 }43. Bb3 *[StudyName "Appendix"][ChapterName "Position 32"][FEN "rn1qkbnr/pp3ppp/2p1p3/3p4/2P3b1/1P2PN1P/P2P1PP1/RNBQKB1R b KQkq - 0 5"]{ Player 2, Concept 4, Phase 1 }5... Bf5 *[StudyName "Appendix"][ChapterName "Position 33"][FEN "r3kb1r/pppq1ppp/2n2n2/Q3p3/2b5/2N2NP1/PP2PPBP/R1B2RK1 w kq - 4 11"]{ Player 2, Concept 5, Phase 1 }11. Qa4 *[StudyName "Appendix"][ChapterName "Position 34"][FEN "4r3/2R3p1/2pBkq2/1pPpn2b/1P3Q2/5PKP/8/3B4 b - - 3 48"]{ Player 2, Concept 1, Phase 1 }48... g5 *[StudyName "Appendix"][ChapterName "Position 35"][FEN "8/p4pk1/1pR2p2/6pp/P3P3/1P3QPP/5P1K/q3r3 b - - 20 43"]{ Player 2, Concept 1, Phase 2 }43... Qd4 *[StudyName "Appendix"][ChapterName "Position 36"][FEN "4kb2/3n3r/1pqp2pp/p2Npp2/2P1b2Q/4B3/PP4PP/2RR2K1 w - - 0 22"]{ Player 2, Concept 5, Phase 2 }22. Qe1 *[StudyName "Appendix"][ChapterName "Position 37"][FEN "2r2r1k/3bqp1p/3ppp2/pp6/4PP2/PB1Q4/1PP3PP/2KR3R b - - 0 19"]{ Player 2, Concept 5, Phase 2 }19... Rg8 *[StudyName "Appendix"][ChapterName "Position 38"][FEN "r1bqk2r/pp1p2bp/2p3nn/5pp1/1PPPp3/PQN3P1/1B2PPBP/R3K1NR w Kkq - 0 12"]{ Player 2, Concept 4, Phase 2 }12. d5 *[StudyName "Appendix"][ChapterName "Position 39"][FEN "2k2b1r/pp4p1/2p1p2p/3rBq2/2QP3P/8/PPP2P2/2KRR3 w - - 3 21"]{ Player 2, Concept 1, Phase 2 }21. Qe2 *[StudyName "Appendix"][ChapterName "Position 40"][FEN "8/1p1r3b/p1p4p/P1PprpkP/1P1R2pR/3BP1P1/3K1P2/8 b - - 9 53"]{ Player 2, Concept 1, Phase 2 }53... Re8 *[StudyName "Appendix"][ChapterName "Position 41"][FEN "r1bqr1k1/pp3ppp/5n2/3pb3/8/1PN1P3/PB1QBPPP/R4RK1 b - - 2 13"]{ Player 2, Concept 4, Phase 2 }13... Bf5 *[StudyName "Appendix"][ChapterName "Position 42"][FEN "rn2kb1r/pp2pppp/2p2n2/q4b2/3PN3/5N2/PPPB1PPP/R2QKB1R b KQkq - 1 7"]{ Player 2, Concept 5, Phase 2 }7... Qd5 *[StudyName "Appendix"][ChapterName "Position 43"][FEN "rn1qk2r/1bp1bppp/1p2pn2/p2p4/2P5/P1N1PNP1/1P1P1PBP/R1BQ1RK1 b kq - 0 8"]{ Player 2, Concept 4, Phase 2 }8... dxc4 *[StudyName "Appendix"][ChapterName "Position 44"][FEN "r2qkb1r/pp3ppp/2n1p3/1B1n1b2/3P4/2N2N2/PP3PPP/R1BQK2R w KQkq - 0 9"]{ Player 2, Concept 5, Phase 2 }9. Qa4 *[StudyName "Appendix"][ChapterName "Position 45"][FEN "8/3qk1p1/8/8/p2p3P/5QP1/5P1K/8 w - - 4 63"]{ Player 3, Concept 7, Phase 1 }63. Qe4+ *[StudyName "Appendix"][ChapterName "Position 46"][FEN "1r1b1r2/pp1b4/2p1p1kp/N1Pp2p1/1P1PP3/5BP1/P4PK1/2R4R b - - 5 27"]{ Player 3, Concept 8, Phase 1 }27... Kg7 *[StudyName "Appendix"][ChapterName "Position 47"][FEN "r2r2k1/4qppp/2n2n2/2pp4/5B2/P3PN2/1P2QPPP/R4RK1 b - - 0 15"]{ Player 3, Concept 9, Phase 1 }15... Ne4 *[StudyName "Appendix"][ChapterName "Position 48"][FEN "4r3/7p/1P3ppk/3Q4/5P2/2q1B2P/5P1K/8 w - - 3 44"]{ Player 3, Concept 9, Phase 1 }44. b7 *[StudyName "Appendix"][ChapterName "Position 49"][FEN "8/8/R4p1k/4p1pP/3n4/6QK/3q4/8 b - - 2 69"]{ Player 3, Concept 8, Phase 1 }69... Qe2 *[StudyName "Appendix"][ChapterName "Position 50"][FEN "2rr2k1/R2nqpp1/1p2p2p/1Pbn4/2Np4/1P4P1/1B2PPBP/1Q1R2K1 w - - 0 20"]{ Player 3, Concept 7, Phase 1 }20. Bxd4 *[StudyName "Appendix"][ChapterName "Position 51"][FEN "5n2/4b1k1/2b1p1pp/2p1p3/1pP1N2P/1P2B2Q/3N1PPK/3q4 b - - 8 32"]{ Player 3, Concept 9, Phase 1 }32... Qe2 *[StudyName "Appendix"][ChapterName "Position 52"][FEN "r5k1/1b2qpp1/p1prp2p/1pR5/1P1PBP2/4P2P/3Q2PK/R7 w - - 3 33"]{ Player 3, Concept 8, Phase 1 }33. g4 *[StudyName "Appendix"][ChapterName "Position 53"][FEN "R7/1pNq1r2/1P1p2nk/2nP1ppp/4p3/4P1P1/5PBP/1Q4K1 w - - 8 31"]{ Player 3, Concept 7, Phase 1 }31. Qa1 *[StudyName "Appendix"][ChapterName "Position 54"][FEN "8/5p1k/7p/5p2/5P2/7r/1r6/2R2KQ1 b - - 27 62"]{ Player 3, concept 7, Phase 2 }62... Rhh2 *[StudyName "Appendix"][ChapterName "Position 55"][FEN "2b1r3/p4qk1/2Q1p2p/1R4p1/8/PB6/KPP5/8 b - - 4 34"]{ Player 3, Concept 9, Phase 2 }34... Qe7 *[StudyName "Appendix"][ChapterName "Position 56"][FEN "3qkb1r/1p3pp1/2p1p1p1/r7/2p3P1/P1Q1P2P/3P1PB1/R3K2R w KQk - 0 18"]{ Player 3, Concept 8, Phase 2 }18. a4 *[StudyName "Appendix"][ChapterName "Position 57"][FEN "8/3R1pk1/p5p1/K1Q4p/8/5q1P/Pb6/8 w - - 7 38"]{ Player 3, Concept 7, Phase 2 }38. Qd5 *[StudyName "Appendix"][ChapterName "Position 58"][FEN "2rqbrk1/1p3pb1/1n2p1p1/1P1p2P1/1QnP1B1p/2N1P3/5PBP/2RRN1K1 b - - 1 24"]{ Player 3, Concept 8 , Phase 2 }24... f6 *[StudyName "Appendix"][ChapterName "Position 59"][FEN "4q3/4rpk1/p1p3p1/Pn1Pp2p/4P2P/3Q2P1/N4P2/1R4K1 w - - 0 34"]{ Player 3, Concept 9, Phase 2 }34. Nb4 *[StudyName "Appendix"][ChapterName "Position 60"][FEN "1rbr1k2/5p2/2p2qp1/p1N4n/4Pp2/1B3P2/PP5R/K5QR w - - 2 37"]{ Player 3, Concept 7, Phase 2 }37. Qc1 *[StudyName "Appendix"][ChapterName "Position 61"][FEN "2r5/7p/5ppk/1PBQ4/5P2/7P/5P1K/4q3 w - - 0 42"]{ Player 3, Concept 7, Phase 2 }42. b6 *[StudyName "Appendix"][ChapterName "Position 62"][FEN "r2rk3/1pp1qpp1/p3p2p/1bPpPP2/3Q2P1/2RBP3/PP4P1/4K2R b K - 0 20"]{ Player 3, Concept 8, Phase 2 }20... Bxd3 *[StudyName "Appendix"][ChapterName "Position 63"][FEN "1rn1r2k/6pp/2Rp3b/3qpp2/1B6/2Q1PN1P/5PP1/2R3K1 b - - 2 30"]{ Player 3, Concept 9, Phase 2 }30... g6 *[StudyName "Appendix"][ChapterName "Position 64"][FEN "r3kb1r/pppq1ppp/2n2n2/Q3p3/2b5/2N2NP1/PP2PPBP/R1B2RK1 w kq - 4 11"]{ Player 4, Concept 10, Phase 1 }11. Qa4 Be6 12. Ng5 *[StudyName "Appendix"][ChapterName "Position 65"][FEN "r1b1k2r/pp1pppbp/2n2np1/q7/3N4/2N1B1P1/PPP1PPBP/R2QK2R w KQkq - 3 8"]{ Player 4, Concept 10, Phase 1 }8. Nb3 Qd8 9. a4 *[StudyName "Appendix"][ChapterName "Position 66"][FEN "r1bq1rk1/p2nbpp1/2p1pn1p/1p6/2BP3B/2N1PN2/PP3PPP/R2Q1RK1 w - - 0 11"]{ Player 4, Concept 10, Phase 1 }11. Bd3 *[StudyName "Appendix"][ChapterName "Position 67"][FEN "rn1qkb1r/pp1b2pp/4pn2/1B1P4/8/2P5/P2N2PP/R1BQK1NR w KQkq - 2 10"]{ Player 4, Concept 10, Phase 1 }10. Bxd7+ *[StudyName "Appendix"][ChapterName "Position 68"][FEN "r1bqk2r/pp1p2bp/2p3nn/5pp1/1PPPp3/PQN3P1/1B2PPBP/R3K1NR w Kkq - 0 12"]{ Player 4, Concept 11, Phase 1 }12. f3 *[StudyName "Appendix"][ChapterName "Position 69"][FEN "8/2p1kp2/6p1/3n4/1p3N2/4nPP1/PNP4P/2K5 b - - 2 39"]{ Player 4, Concept 12, Phase 1 }39... Nc3 *[StudyName "Appendix"][ChapterName "Position 70"][FEN "2r1qr1k/3nb3/bpp1p3/p2p1ppp/2PP4/1PBN2P1/P1Q1PPBP/2R2RK1 w - - 0 21"]{ Player 3, Concept 11, Phase 1 }21. Qd2 *[StudyName "Appendix"][ChapterName "Position 71"][FEN "3R4/5p1k/2P4p/5p2/2b2P2/8/1r2r1BP/2R4K w - - 1 45"]{ Player 4, Concept 12, Phase 1 }45. c7 *[StudyName "Appendix"][ChapterName "Position 72"][FEN "3r4/6k1/2p3qp/1pPrPpp1/1P1P3P/4QRP1/3R3K/8 w - - 5 44"]{ Player 4, concept 12, Phase 1 }44. Rf1 *[StudyName "Appendix"][ChapterName "Position 73"][FEN "8/8/2Q5/6p1/1p1q4/7P/2P3PK/2k5 b - - 33 58"]{ Player 4, Concept 11, Phase 1 }58... g4 *[StudyName "Appendix"][ChapterName "Position 74"][FEN "1r3rk1/1ppqnbbp/p2p2p1/5p2/2PN1P2/1P2P1P1/PB1Q2BP/R4RK1 w - - 1 18"]{ Player 4, Concept 13, Phase 1 }18. Rad1 *[StudyName "Appendix"][ChapterName "Position 75"][FEN "7k/3R4/1p3rpp/p3Q3/6PP/P3Pq2/1P6/6K1 w - - 37 72"]{ Player 4, Concept 11, Phase 1 }72. Rf7 *[StudyName "Appendix"][ChapterName "Position 76"][FEN "1r6/2pbk3/2n1p3/1pPp1p2/1P1P1P2/4PK1B/R6P/4B3 w - - 5 42"]{ Player 4, Concept 13, Phase 1 }42. Bf1 *[StudyName "Appendix"][ChapterName "Position 77"][FEN "2r3k1/1br2pp1/pnpqp2p/1p2N3/3P4/4PB1P/1PRQ1PP1/2R3K1 w - - 8 25"]{ Player 4, Concept 13, Phase 1 }25. Qa5 *[StudyName "Appendix"][ChapterName "Position 78"][FEN "6k1/p4ppb/3N4/3P1P2/P2p4/1P3Q1P/6K1/4q3 b - - 0 37"]{ Player 4, Concept 12, Phase 1 }37... Qe5 *[StudyName "Appendix"][ChapterName "Position 79"][FEN "r4rk1/p2qppbp/1p1p1np1/8/2P5/1PNQ2P1/P2BPPKP/3R1R2 w - - 0 15"]{ Player 4, Concept 13, Phase 1 }15. Bg5 *[StudyName "Appendix"][ChapterName "Position 80"][FEN "r5k1/3rqpb1/b2ppnpp/PN6/3Pn3/4QN1P/5PPB/R1R2BK1 w - - 4 27"]{ Player 4, Concept 10, Phase 2 }27. Rc6 *[StudyName "Appendix"][ChapterName "Position 81"][FEN "r4k2/1R3p2/3P2p1/p3P2p/1p1K3P/6P1/5P2/8 b - - 0 47"]{ Player 4, Concept 12, Phase 2 }47... a4 *[StudyName "Appendix"][ChapterName "Position 82"][FEN "r2qkbr1/pppb1p1p/2nnp3/3p2p1/3PP3/2N1BP2/PPPQ2PP/2KR1BNR b q - 6 10"]{ Player 2, Concept 10, Phase 2 }10... Na5 *[StudyName "Appendix"][ChapterName "Position 83"][FEN "r7/2r1bkp1/q4p1P/1p2pQp1/8/1P2B3/P2R1P2/1K1R4 w - - 0 1"]{ Player 4, Concept 11, Phase 2 }1. Qh7 *[StudyName "Appendix"][ChapterName "Position 84"][FEN "5k2/8/p1p1pp1p/N1P3p1/8/7R/P2rr2P/2R4K w - - 3 34"]{ Player 4, Concept 12, Phase 2 }34. Nxc6 *[StudyName "Appendix"][ChapterName "Position 85"][FEN "1q2r3/1prn1p1k/p2p1np1/P2P3p/NPB1P3/5P1P/3Q1P2/2R3RK w - - 1 26"]{ Player 4, Concept 13, Phase 2 }26. Bd3 *[StudyName "Appendix"][ChapterName "Position 86"][FEN "4R3/6kp/1n3rp1/pNp5/P3N3/1P2b2P/6PK/8 b - - 4 38"]{ Player 4, Concept 12, Phase 2 }38... Rf1 *[StudyName "Appendix"][ChapterName "Position 87"][FEN "3r2k1/7p/2r1RQpB/p2p4/P1p5/3n3P/6PK/1q6 b - - 0 39"]{ Player 4, Concept 12, Phase 2 }39... Qb8+ *[StudyName "Appendix"][ChapterName "Position 88"][FEN "5k1r/1pN1ppb1/1p1p2pn/7p/8/5Q2/5PPP/5K2 w - - 1 23"]{ Player 4, Concept 11, Phase 2 }23. Qxb7 *[StudyName "Appendix"][ChapterName "Position 89"][FEN "r1bqkbnr/3p1ppp/pp2p3/2n5/4P3/P1N2P2/1PP3PP/R1BQKBNR w KQkq - 0 7"]{ Player 4, Concept 10, Phase 2 }7. b4 *[StudyName "Appendix"][ChapterName "Position 90"][FEN "r2q2k1/1br2pp1/p1p1p2p/1pR5/1P1P4/4PB1P/3Q1PP1/2R3K1 w - - 1 29"]{ Player 4, Concept 13, Phase 2 }29. Qa2 *[StudyName "Appendix"][ChapterName "Position 91"][FEN "5bk1/pp1q1rp1/n1pp3p/5r2/2PP4/1Q3NB1/PP4PP/R3R1K1 w - - 2 22"]{ Player 4, Concept 13, Phase 2 }22. Rad1 *[StudyName "Appendix"][ChapterName "Position 92"][FEN "rnbq1rk1/pp1pppbp/5np1/P7/2PN4/3BP3/1P1B1PPP/RN1Q1RK1 b - - 2 10"]{ Player 4, Concept 10, Phase 2 }10... d5 *[StudyName "Appendix"][ChapterName "Position 93"][FEN "r1bqk2r/1ppn1pbp/p2p2p1/3Pp3/2P1P3/P1NBBQ1P/1P3PP1/R3K2R w KQkq - 0 12"]{ Player 4, Concept 13, Phase 2 }12. Qe2 *[StudyName "Appendix"][ChapterName "Position 94"][FEN "8/1r3p1k/3p2pp/4n3/1Pp1P1Pq/R1P1Q2P/P3B2r/K2R4 w - - 1 49"]{ Player 4, Concept 11, Phase 2 }49. Bf1 *[StudyName "Appendix"][ChapterName "Position 95"][FEN "3r1bk1/4np1p/q3pnp1/p1BpN3/P2P1PP1/1P3B2/4Q2P/5RK1 b - - 2 28"]28... Qa8 { Player 4, Concept 11, Phase 2 } *
